# Establishing Heat Alert Thresholds for the Varied Climatic Regions of British Columbia, Canada

**DOI:** 10.3390/ijerph15092048

**Published:** 2018-09-19

**Authors:** Kathleen E. McLean, Rebecca Stranberg, Melissa MacDonald, Gregory R. A. Richardson, Tom Kosatsky, Sarah B. Henderson

**Affiliations:** 1 British Columbia Centre for Disease Control, 655 West 12th Ave, Vancouver, BC V5Z 4R4, Canada; tom.kosatsky@bccdc.ca (T.K.); sarah.henderson@bccdc.ca (S.B.H.); 2 Climate Change and Innovation Bureau, Healthy Environments and Consumer Safety Branch, Health Canada, 269 Laurier Avenue West, Ottawa, ON K1A 0K9, Canada; rebecca.stranberg@canada.ca (R.S.); gregory.richardson@canada.ca (G.R.A.R.); 3 National Health and Air Quality Program, Meteorological Service of Canada, Environment and Climate Change Canada, 45 Alderney Dr, Dartmouth, NS B2Y 2N6, Canada; melissa.macdonald@canada.ca

**Keywords:** extreme heat, temperature, mortality, public health surveillance, heat alert and response system, climate change, early warning systems

## Abstract

Following an extreme heat event in 2009, a Heat Alert and Response System (HARS) was implemented for the greater Vancouver area of British Columbia (BC), Canada. This system has provided a framework for guiding public health interventions and assessing population response and adaptation to extreme heat in greater Vancouver, but no other parts of BC were covered by HARS. The objective of this study was to identify evidence-based heat alert thresholds for the Southwest, Southeast, Northwest, and Northeast regions to facilitate the introduction of HARS across BC. This was done based on a national approach that considers high temperatures on two consecutive days and the intervening overnight low, referred to as the high-low-high approach. Daily forecast and observed air temperatures and daily mortality counts for May through September of 2004 through 2016 were obtained. For each date (day_t_), day_t−2_ forecasts were used to assign high temperatures for day_t_ and day_t+1_ and the overnight low. A range of high-low-high threshold combinations was assessed for each region by finding associations with daily mortality using time-series models and other considerations. The following thresholds were established: 29-16-29 °C in the Southwest; 35-18-35 °C in the Southeast; 28-13-28 °C in the Northwest; and 29-14-29 °C in the Northeast. Heat alert thresholds for all regions in BC provide health authorities with information on dangerously hot temperature conditions and inform the activation of protective public health interventions.

## 1. Introduction

Exposure to extreme heat has direct and indirect health effects. Heat stress can directly induce illnesses such as heat edema, rash, cramps, syncope, exhaustion, and heat stroke [1]. Heat stress can indirectly increase the likelihood of severe adverse health events including cardiovascular mortality [2], stroke, and renal colic [3]. There is also increased risk of adverse maternal [4] and birth outcomes [5], including gestational diabetes [4], sudden infant death syndrome [5], term placental abruption [6], and early delivery [7]. Use of antipsychotics, antidepressants, diuretics [8], and illicit substances such as cocaine [9] can increase the likelihood of experiencing heat-related illness or experiencing adverse health events because these drugs interfere with natural thermoregulatory mechanisms [1]. This spectrum of health outcomes is influenced by the risk and protective factors within each population, and the intensity, duration, and timing of the heat event.

Extreme heat is a leading cause of illness and death from weather-related hazards in Canada. For example, there were an estimated 114 excess deaths during a 5-day event in greater Vancouver, British Columbia (BC) in the summer of 2009 [10]. Similarly, there were an estimated 106 excess deaths during the 3-day event in Montréal, Quebec in the summer of 2010 [11]. In addition, mean estimates for the Canadian cities of Montréal, Toronto, Ottawa, and Windsor were 121, 120, 41, and 37 excess annual heat-related deaths, respectively, between 1954 to 2000 [12]. Heat exposure has also been associated with increased ambulance dispatches [13] and emergency room visits [14]. For example, a study in Toronto found an increase (95% confidence interval) of 32% (24%, 41%) in ambulance dispatches for heat-related illnesses associated with every 1 °C increase in mean temperature [13]. Another study in rural southern Ontario found that the average rate of emergency room visits was 11% (7%, 15%) higher during heat events compared with average summer temperatures [14].

Maximum temperatures during a heat event are strongly associated with the magnitude of the observed health outcomes. The temperature thresholds at which Canadian populations exhibit increased morbidity and mortality vary by location across the country. For example, mortality curves generated for 21 Canadian cities showed an increase in relative risk above baseline at mean temperatures as low as 20 °C (Calgary, Alberta) and as high as 27 °C (Windsor, Ontario) [15]. Within BC, a similar study found increased risk over maximum apparent temperatures as low as 14 °C in the north, and as high as 22 °C in the south [16]. This variability is due to several factors, including: different adaptive capacities, such as access to air conditioners [17]; population and demographic differences; historical meteorological events, especially those hot enough to drive adaptation activities; and, most importantly, historical climate. To account for all of these factors, heat alert thresholds would be expected to differ by region [18].

Environment and Climate Change Canada (ECCC) is responsible for issuing timely weather forecasts, warnings, and alerts across Canada, including heat alerts. Prior to 2015, ECCC issued heat alerts using a single national criterion based on the Canadian humidex, without consideration of different climates or population responses. At present, ECCC is modernizing the national heat alert program to incorporate regional climatology, health evidence, heat event duration, and overnight temperatures. Under this updated program, heat alerts are issued based on forecast high temperatures for two consecutive days and the intervening overnight low (referred to herein as the high-low-high approach). In some jurisdictions, ECCC has partnered with provincial public health agencies and Health Canada to complete the analyses necessary to identify evidence-based high-low-high thresholds. In other regions, thresholds are currently based on guidance from the World Health Organization (WHO) using the 95th percentiles of daily high and low temperatures [19].

The BC Centre for Disease Control (BCCDC) collaborated with ECCC, Health Canada, and BC health authorities to establish high-low-high alert thresholds across all regions of BC. As of 2017, the only operational Heat Alert and Response System (HARS) in BC was restricted to the greater Vancouver area [10]. Within this HARS, a heat alert was issued on day_t_ for day_t+1_ when the average of the day_t_ 14:00 observed temperature and day_t+1_ forecast high temperature equaled or exceeded 34 °C at Abbotsford airport or 29 °C at Vancouver airport [20]. These thresholds were used from 2011 to 2017. Although no heat alerts were issued during this period, temperatures approached the threshold values on multiple occasions. Whenever this occurred, the BCCDC contextualized the temperature data with real-time data on registered deaths and an evaluation of results from a mortality nowcasting model and shared this information with health authority partners.

At the end of every summer, the BCCDC assessed the operation of the greater Vancouver HARS through retrospective evaluation of forecast temperatures, observed temperatures, and observed mortality to ensure that no important events were missed. This assessment process identified a period of prolonged heat in late June and early July 2015 that affected greater Vancouver but was not identified in real-time. The same episode also affected other regions of BC that did not have established HARS. Previous work has shown that populations in the coastal and northern regions of BC are susceptible to heat impacts despite the temperate climate of these regions [16], further highlighting the need for more comprehensive HARS in the province.

One of the first steps in establishing HARS is to determine appropriate heat alert thresholds for different regions with varying climates and vulnerabilities, and a number of different approaches have been used worldwide. Synoptic classification systems identify air-mass categories using several meteorological factors and then assess excess mortality within each category [19,21]. Other systems model the relationship between mortality and maximum temperatures, minimum temperatures, or apparent temperatures, using single-day values or multi-day averages [22,23]. Many systems have different alert levels (i.e., heat warning versus heat emergency) with thresholds for each level reflecting a certain percentage increase in morbidity or mortality, which varies by jurisdiction [19,21]. In Canada, the new high-low-high approach being used by ECCC required that we use methods tailored to this approach. Here we describe the process of establishing the high-low-high thresholds for four regions of BC. For each region, we identified thresholds that were (1) reliably associated with increased population mortality, and (2) unlikely to cause warning fatigue. We also discuss how such thresholds should be used for ongoing assessment of health vulnerabilities and climate change adaptation in the context of public health surveillance.

## 2. Materials and Methods

The R statistical computing environment version 3.4.2 (28 September 2017) (R Foundation for Statistical Computing, Vienna, Austria) was used for all data management, analysis, and visualization [24]. Sample code and data for the main threshold analyses are included in the Supplementary Materials.

### 2.1. Study Context

**Figure 1:**
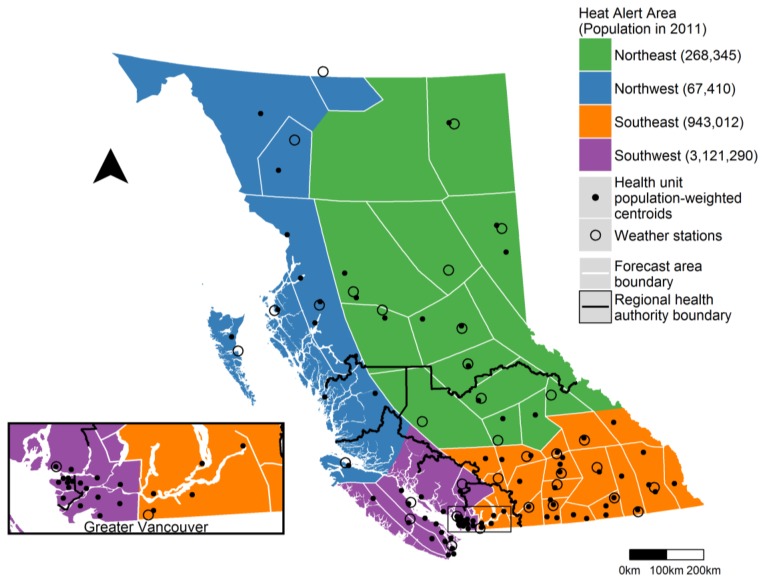
Map of Heat Alert Areas, forecast areas, weather stations, health unit population-weighted centroids, and regional health authority boundaries in British Columbia (BC), Canada.

The province of BC, which covers a land area of 944,735 km^2^, is located on the west coast of Canada. The estimated 2017 provincial population was 4.84 million, of which 2.78 million lived in the greater Vancouver area on the southwestern coast (Figure 1). In general, the climate along the entire BC coast is temperate throughout the year, while the inland climate is more extreme with colder winters and hotter summers. Winter conditions are typically coldest in the north, especially in the Boreal region. Summer conditions are typically hottest throughout the southern interior, especially in the dry plateau between the Coast Mountain and Rocky Mountain ranges.

### 2.2. Heat Alert Areas

In collaboration with ECCC, the BCCDC grouped the 53 provincial forecast regions into four climatologically similar Heat Alert Areas (HAAs). We then assigned each of the 89 provincial health units to one of the HAAs using the spatial distribution of the 2011 census population (Figure 1). First, we assigned the populations of all 7582 dissemination areas in BC (each with 400–700 residents) to their geographic centers. Next, we used all dissemination areas within the boundary of each health unit to calculate its population-weighted center. Finally, each health unit was assigned to the HAA that contained its population-weighted center. Populations within each HAA ranged from 67,410 in the Northwest to 3,121,290 in the Southwest, where the latter includes most of the densely populated greater Vancouver area.

### 2.3. Temperature Forecasts and Observations

We obtained three sets of daily high and low temperature forecasts for May through September of 2004 through 2016 for 35 ECCC forecast areas (Figure 1). Official public forecasts, including temperature, are issued three times daily by ECCC at approximately 05:00, 11:00, and 16:00 local time. Operational meteorologists use computer models, expert knowledge of local weather, climatology, and nowcasting to provide temperature forecasts for each area. In forecast areas with local effects contributing to large temperature differences across the area, exceptions are used to describe varied conditions. For example, summer forecasts for the City of Vancouver are often described as “high 22 °C except 28 °C inland” to account for the cooler temperatures experienced in coastal locations compared with inland locations away from sea breezes. We did not consider temperature exceptions in these analyses, and thus would have used the forecast high of 22 °C from the example above. We also obtained daily observations of high (maximum) and low (minimum) temperatures for one weather station in each forecast area over the same period (Figure 1). All forecast and observed temperatures were received as integer values in degrees Celsius (°C). For each date of the study period (day_t_), we used the day_t−2_ afternoon forecast to assign the high-low-high forecast temperatures for day_t_ and day_t+1_. For each forecast area, we then computed the Pearson correlation coefficient for the forecast (on day_t−2_) and observed (on day_t_) temperatures and the slope of the relationship between them.

### 2.4. Mortality

Information on all-cause mortality was obtained from the BC Vital Statistics Agency, which provides the BCCDC with a daily feed of registered deaths for its surveillance activities. These anonymous data include the date of death, primary cause of death (coded according to the 10th revision of the International Classification of Diseases), age, sex, health unit, and residential 6-digit postal code for each decedent. Based on our previous work indicating that heat events are associated with increases in both accidental and non-accidental deaths [10], our analyses used daily counts of deaths from all causes. All deaths in each HAA were aggregated on a daily basis, and a running 3-day sum of the daily counts (including day_t−1_, day_t_, and day_t+1_) was used in the analyses. This allowed us to amplify the cumulative effects of heat, particularly in the less populous HAAs where daily death counts are low.

### 2.5. Time-Series Modelling

We tested a range of high-low-high threshold combinations for each HAA, chosen from the distributions of summertime temperatures observed in each area. For each date (day_t_), we assigned a heat alert category based on the forecast temperatures. This category was equal to the sum of the total degrees by which the daytime highs and the overnight lows deviated below the candidate high-low-high thresholds. If a forecast was greater than or equal to its corresponding threshold, it would contribute 0 °C to the total. If a forecast was 1 °C lower than its threshold, it would contribute 1 °C to the total, and so on. The total sum of these differences was used to assign dates to heat alert categories as follows: Category 0 = 0 °C; Category 1 = 1 °C; Category 2 = 2 °C; and Category 3 = 3 °C or more (Table 1). This approach was taken to evaluate the effects of scenarios when forecasts nearly reached the threshold values and to account, in part, for the variability in forecast values within forecast areas.

We estimated the effect of forecast temperatures on mortality by constructing time-series models with the running 3-day sum of mortality as the response variable and the heat alert category as the explanatory variable. We focused on forecast temperatures for these analyses because heat alerts will always be generated using such uncertain data. We used generalized linear models with a natural cubic spline having six degrees of freedom to account for long-term temporal trends. The resulting effect estimates were the relative rates of mortality associated with Category 0, Category 1, and Category 2 heat, all compared with Category 3. The analyses were run for each of the 35 forecast areas, where heat alert categories for the area were regressed against all deaths in the HAA to which it belonged. Finally, we combined the model results for forecast areas in the same HAA using random effects meta-analysis from the R meta package [25].

### 2.6. Identification of Heat Alert Thresholds

To evaluate the high-low-high combinations, we generally considered the following features as desirable in the following order: (1) minimization of warning fatigue, such that health authorities and the general public would retain confidence in the heat alerting system; (2) threshold values at or over the 95th percentile of the distribution, as per WHO guidelines [19]; (3) at least a 5% increase in all-cause mortality; (4) an increase in risk across Category 0, Category 1, and Category 2 heat; and (5) consistency with neighboring Canadian jurisdictions (i.e., Alberta, Yukon, and the Northwest Territories). For all high-low-high combinations, we assessed the potential for warning fatigue by identifying the number of times a heat alert would have been issued under those thresholds using both observed and forecast temperatures. Sets of days that met or exceeded the thresholds with a one-day gap between them were counted as a single warning because that is how ECCC would operate in practice. Based on these analyses, the BCCDC recommended thresholds for each HAA to ECCC, which then finalized the values following internal and external operational review.

### 2.7. Sub-Analyses

We conducted sub-analyses for age at death, place of death, and within-season variability using the finalized heat alert thresholds. For these analyses, we used heat alert categories based on observed temperatures, and combined them into heat events (Categories 0–2) and other summer days (Category 3). We focused on observed temperatures for these analyses because we wanted to understand the actual effects of hot days. In all other ways, the sub-analyses were the same as the analyses done for threshold identification using forecast temperatures. For age at death, we constructed models separately for deaths at all ages and for deaths in those aged 65–75 years based on previous work indicating this age range being at higher risk [26]. For place of death, we constructed models separately for deaths that occurred in all locations, out of hospital (including deaths at residential care facilities), and out of care (deaths at home or the community). To examine within-season variability, we constructed models separately for deaths that occurred during May–September (entire season), May–July (early season), and July–September (late season).

## 3. Results

### 3.1. Observed and Forecasted Temperatures

Observed summertime daily lows from 2004 to 2016 ranged from −14.5 °C to 26.6 °C across BC, while observed daily highs ranged from 0.5 °C to 42 °C. The 95th percentiles of observed low temperatures were generally highest in the Southwest (15 °C) and Southeast (16 °C) HAAs, and lowest in the Northeast (13 °C) and Northwest (14 °C) HAAs. The 95th percentiles of observed high temperatures were generally lowest in the Northwest HAA (25 °C), similar in the Northeast and Southwest HAAs (30 °C), and highest in the Southeast HAA (34 °C). The forecast low and high temperatures followed the same pattern as the observed temperatures.

In all 35 forecast areas, forecast low temperatures were positively correlated with observed low temperatures (Figure S1) and forecast high temperatures were positively correlated with observed high temperatures (Figure S2). For overnight lows the Pearson correlation coefficients by forecast area ranged from 0.72 to 0.91 (Figure S1), and for daytime highs the correlation coefficients ranged from 0.78 to 0.92 (Figure S2). For the linear relationship between observed (dependent variable) and forecast (independent variable) temperatures, the mean slope was 0.93 for both daily lows and daily highs. Forecast lows over-predicted observed lows in 89% of forecast areas (Figure S1), and forecast highs over-predicted observed highs in most forecast areas (97%) (Figure S2).

### 3.2. Heat Alert Thresholds

**Figure 2:**
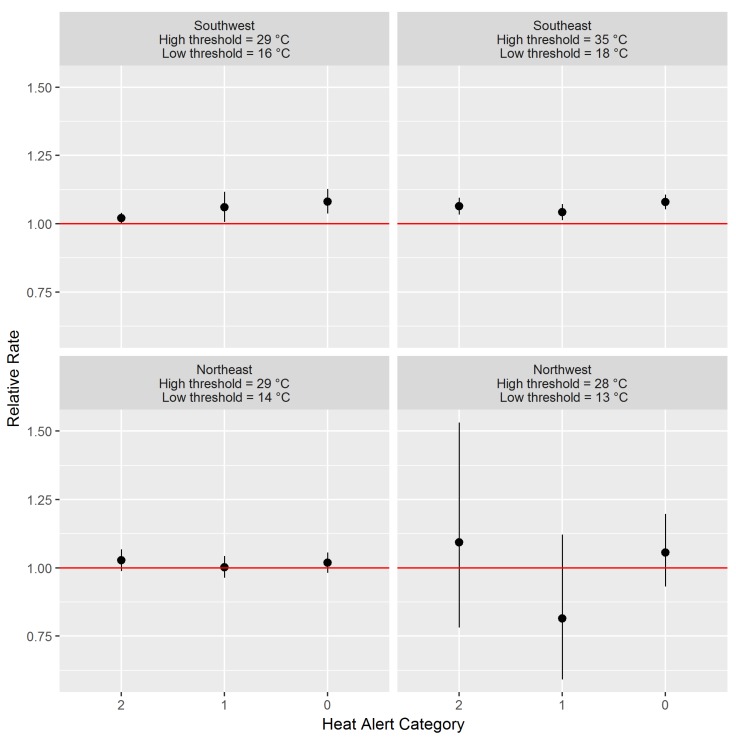
Relative rates and 95% confidence intervals for mortality during Category 0, Category 1, and Category 2 heat compared with Category 3 heat using the finalized high-low-high heat alert thresholds in each Heat Alert Area (HAA).

We established the following high-low-high heat alert thresholds: 29-16-29 °C in the Southwest; 35-18-35 °C in the Southeast; 29-14-29 °C in the Northeast; and 28-13-28 °C in the Northwest (Figure 2, Figures S4–S7). In the Southwest HAA the relative rate of mortality (95% confidence interval) for Category 0 compared with Category 3 was 1.08 (1.04, 1.13) (Figure 2). In comparison, Category 1 was 1.06 (1.01, 1.12) and Category 2 was 1.02 (1.00, 1.04) (Figure 2). There were other high-low-high combinations that followed the same pattern in the Southwest HAA (Figure S4), but 29-16-29 °C was most consistent with the criteria described above. In the Southeast HAA the relative rates of mortality for Categories 0, 1, and 2 were 1.08 (1.05, 1.11), 1.04 (1.01, 1.07), and 1.06 (1.03, 1.09), respectively (Figure 2). There were significant associations for all high-low-high combinations tested for the Southeast HAA (Figure S5), and the 35-18-35 °C values were based largely on minimizing warning fatigue. In the Northeast and Northwest HAAs, none of the relative mortality rates for any of the categories were significant (Figure 2). In the Northeast, all values were above 1.00 and below 1.03. In the Northwest, the value for Category 1 was less than 1.00 while the values for Categories 0 and 2 were above 1.00 but below 1.10. The 29-14-29 °C and 28-13-28 °C thresholds selected for these HAAs were based on both the 95th percentile values of the observed temperatures and continuity with warning criteria from neighboring jurisdictions.

### 3.3. Retrospective Heat Alerts Using Forecasted and Observed Temperatures

The average number of heat alerts per year (2004–2016) based on forecast temperatures ranged from zero in multiple forecast areas to four in Clinton (Northeast HAA) (Table 2). In 74% of forecast areas the average number of annual heat alerts based on observed temperatures was lower than those based on forecast temperatures, suggesting a greater likelihood of false positives (an alert not being needed when it was issued) in these areas. In 17% of forecast areas, the average number of annual heat alerts based on observed temperatures was higher than those based on forecast temperatures. This suggests a greater likelihood of false negatives (an alert being missed when it was needed) in Vancouver, Victoria, Kamloops, Penticton, Dease Lake, and Sandspit (Table 2).

**Figure 3:**
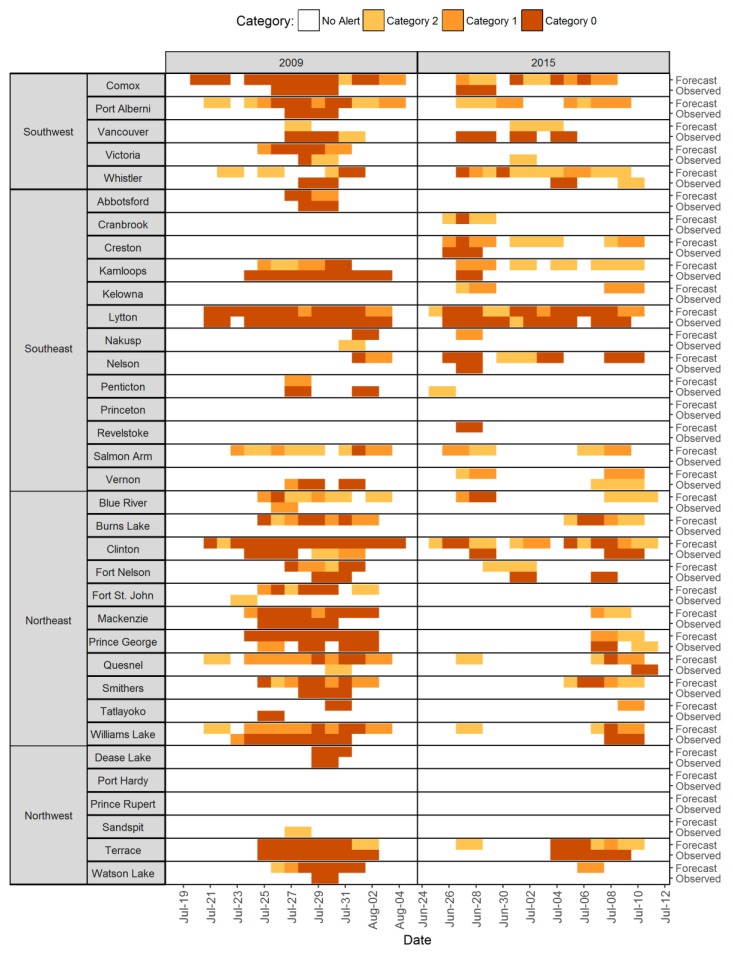
Retrospective heat alerts during two extreme heat events that affected BC in 2009 and 2015 based on forecasted and observed temperatures and the finalized heat alert thresholds.

We also looked at retrospective heat alerts during two extreme heat events that affected all of BC in 2009 and 2015 using both forecast and observed temperatures classified into the three heat categories (Figure 3). From 19 July to 4 August 2009, 25 of the 35 (71%) forecast areas had days with observed temperatures near to or meeting the threshold values. In 21 of those areas, the observed temperatures coincided with periods of forecast heat, while in four areas the observed heat was not predicted. There were also three areas for which heat was forecast but not observed. The 2015 event was less extreme than the 2009 event. Between 25 June and 11 July 2015, only 16 of the 35 (46%) forecast areas had at least one period of observed heat according to the heat alert thresholds. In 14 of those locations the observations coincided with periods of forecast heat. There were 12 forecast areas for which heat was predicted but not observed, which is a greater number of false positive warnings than during the 2009 event (Figure 3).

### 3.4. Sub-Analyses

**Figure 4:**
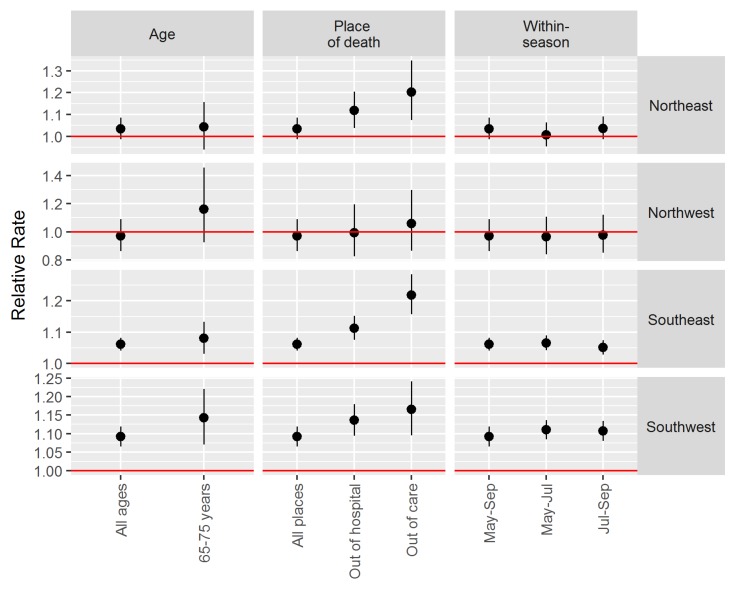
Relative rates and 95% confidence intervals for mortality during heat events (Categories 0–2) compared with other summer days (Category 3) by age at death, place of death, and within-season variability using the finalized high-low-high heat alert thresholds.

The relative rate of mortality during observed heat events (Categories 0–2) compared with all other summer days (Category 3) was higher for those aged 65–75 years than for deaths at all ages in the Southwest and Northwest HAAs (Figure 4). There was no apparent difference in rates between these age groups in the Southeast and Northeast HAAs, and confidence intervals overlapped in all cases. There was a consistent trend across HAAs for the sub-analysis on place of death (Figure 4), with the highest risk observed for those deaths occurring at home or in the community (out of care). This trend was weakest in the Northwest where hot weather is rare, and strongest in the Southeast where extremely hot weather is common. There were consistent effects for the entire season compared with the early and late summer periods (Figure 4).

## 4. Discussion

We identified high-low-high heat alert thresholds for four regions in BC as a necessary step in establishing HARS province-wide. We considered multiple factors in selecting the thresholds including minimization of warning fatigue, distributions of forecast and observed temperatures, evidence-based associations with daily mortality, and consistency with neighboring Canadian jurisdictions. We are confident that the high-low-high thresholds for the Southwest and Southeast HAAs will accurately and flexibly capture periods of increased risk of overall mortality. In the Northeast and Northwest HAAs, however, we did not observe any consistent or significant increases in overall mortality during heat events identified using the high-low-high approach. Northern BC is sparsely populated and hot weather is rare, which makes it challenging to detect an acute temperature-mortality effect using these methods. As such, we placed more emphasis on other factors, including the 95th percentiles of daily high and low observed temperatures, and consistency with neighboring jurisdictions. For example, the Northeast threshold of 29-14-29 °C is one degree below the 95th percentile of observed maximum daily temperatures (30 °C) and one degree above the 95th percentile of observed minimum daily temperatures (13 °C), but it is the same as the threshold for the Northern Prairie region of the neighboring province of Alberta.

Identifying the final heat alert thresholds was a more collaborative and iterative process among stakeholders than we can succinctly describe here. In brief, the BCCDC proposed an initial set of thresholds to ECCC based on the reported analyses and results. The thresholds were then modified slightly after an operational review by ECCC, and presented to the five regional health authorities, all of which have some area in at least two of the HAAs (Figure 1). This led to further modifications, particularly for the Southwest region where the original proposal was for 28-15-28 °C. Because the densely populated greater Vancouver area spans the Southwest and Southeast HAAs and is administered by two different health authorities, we had to ensure that the two sets of thresholds identified similar alert periods in the historic data. Once complete, ECCC took a number of steps to operationalize the finalized thresholds by: (1) preparing standard operating procedures for forecasters; (2) preparing standardized impact and call to action statements that are available to forecasters for heat health messaging; and (3) establishing a communications strategy to ensure timely notification of public health authorities when extreme heat is expected.

Overall, minimization of warning fatigue was the most important consideration in finalizing the heat alert thresholds. Warning fatigue occurs when people become desensitized to warnings after hearing recurring messages about an event that did not materialize, thereby reducing vigilance and preparation in future [27]. Factors such as trust and credibility, over-warning, false alarms, skepticism, and helplessness all contribute to warning fatigue, which must be managed with carefully designed systems and risk communication strategies [27]. Too many heat alerts in a particular region could result in warning fatigue, especially given the uncertainty of temperature forecasts. In BC we found that forecasts consistently over-predicted the observed high and low temperatures (Figures S1 and S2). It follows that there were more heat alerts per year based on forecast temperatures than on observed temperatures (Table 2, Figure 3), with the exception of some forecast areas including Vancouver, Victoria, Kamloops, and Penticton. These four areas are densely populated and located in HAAs with significant increases in mortality when temperatures meet or exceed the heat alert thresholds, and as such these results highlight the need for ongoing evaluation of the high-low-high thresholds established here.

The sub-analyses provided valuable insight into the utility of the final heat alert thresholds, particularly in the northern HAAs. First, the analysis on within-season variability suggested that thresholds should remain the same throughout the summer. Second, the analysis by age group found that risk was generally higher in those aged 65–75 years than risk for all ages, even in the Northwest HAA where the relative rate of mortality for all ages was less than 1.0. This is consistent with our prior work in the greater Vancouver area [10,26], and continues to suggest that people 65–75 years of age are a higher risk group in BC. Equal risk between age groups in the Northeast HAA may be due to significantly lower life expectancy in this region [28]. Finally, the analysis on location of death showed clear and consistent patterns across all four HAAs. One hallmark of extreme heat events is an increased number of deaths occurring at home and in the community [26,29,30]. As such, it was important for our high-low-high thresholds to reflect this risk, even in HAAs where the overall association with mortality was largely null. Risk of mortality at home or in the community was clearly and significantly increased in the Northeast, Southwest, and Southeast HAAs, and elevated in the Northwest HAA.

One limitation of this work was its dependence on mortality as the indicator of population health effects from extreme heat. There are studies of other indicators available for other Canadian jurisdictions, including emergency room visits [14] and ambulance dispatches [13]; however, we did not have such data available for these analyses. Future work to establish heat alert thresholds could be strengthened by considering the combined effects of multiple outcomes that are known to be associated with heat to ensure that identified thresholds are appropriately predictive of morbidity and mortality. Another limitation involves the large differences in population sizes between the HAAs, which simply reflects the reality of BC and many other places in Canada. While it may have improved the analyses to have smaller HAAs with more evenly sized populations, it would have been too challenging for ECCC to operationalize.

Based on previous experience in the greater Vancouver area, operational heat alert thresholds and HARS provide a framework for routine and ongoing assessment of population response to extreme heat. Best practice requires evaluation of the heat alert thresholds and HARS at the end of every summer to ensure that they remain appropriately protective for the upcoming year [31]. The thresholds presented here will first be assessed after the summer of 2018 using analyses similar to those we have described. For each HAA, the BCCDC plans to: (1) characterize any heat alerts that occurred during the summer in terms of forecast and observed temperatures and daily mortality; (2) identify false positive (forecast but not observed) and false negative (observed but not forecast) heat alerts; (3) identify anomalies in daily mortality and deaths out of care, and assess the temperatures before and during those anomalies; (4) evaluate whether lower thresholds would capture any important events that were missed; and (5) evaluate whether higher thresholds would reduce false positives and warning fatigue. This type of ongoing and systematic evaluation process is critical to ensure heat alert thresholds and HARS are effective. It can also play an important role in helping public health authorities to track and understand the evolving relationships between temperature and population health in a changing climate.

## 5. Conclusions

Identification of heat alert thresholds covering all of BC is a necessary step for establishing HARS beyond greater Vancouver such that populations can be better protected from extreme heat. Under the new high-low-high criteria being used by ECCC, we established very different alerting thresholds ranging from 28-13-28 °C to 35-18-35 °C for the four large geographic regions of BC, even though the overall climate of the province is temperate. We also found that the thresholds indicated higher risk of out-of-hospital mortality, which is a hallmark of extreme hot weather events. These findings highlight the value of data-driven methods for establishing heat alert thresholds and associated HARS. The methods and processes applied in BC provide a concrete example that can inform researchers, policy-makers, and public health practitioners conducting similar exercises in other jurisdictions. They also provide a framework for ongoing assessment of health vulnerabilities and climate change adaptation in the context of public health surveillance, as will be conducted in BC over the coming years.

## Figures and Tables

**Table 1:** Example showing how different hypothetical scenarios would be categorized into heat alert categories 0, 1, 2, or 3 based on a high-low-high heat alert threshold of 30-15-30 °C.

Scenarios	Day_t_ High (Deviation below Threshold)	Overnight Low (Deviation below Threshold)	Day_t+1_ High (Deviation below Threshold)	Category (Sum of Deviation below Threshold, Maximum of 3)
Example threshold values	30	15	30	-
Scenario 1	31 (0)	16 (0)	34 (0)	0
Scenario 2	30 (0)	15 (0)	30 (0)	0
Scenario 3	29 (1)	16 (0)	30 (0)	1
Scenario 4	32 (0)	14 (1)	32 (0)	1
Scenario 5	31 (0)	17 (0)	28 (2)	2
Scenario 6	32 (0)	14 (1)	29 (1)	2
Scenario 7	29 (1)	14 (1)	29 (1)	3
Scenario 8	28 (2)	16 (0)	29 (1)	3
Scenario 9	30 (0)	15 (0)	27 (3)	3
Scenario 10	29 (1)	14 (1)	28 (2)	3

**Table 2:** Average number of heat alerts per year based on forecasted and observed temperatures by forecast area from 2004 to 2016 using the finalized high-low-high heat alert thresholds.

Heat Alert Area	Forecast Area	Average Number of Heat Alerts Per Year Based on Forecasted Temperatures	Average Number of Heat Alerts Per Year Based on Observed Temperatures
Southwest	Comox	2	1
	Port Alberni	2	0
	Vancouver	1	2
	Victoria	0	1
	Whistler	2	1
Southeast	Abbotsford	1	0
	Cranbrook	2	0
	Creston	2	1
	Kamloops	1	2
	Kelowna	1	0
	Lytton	3	2
	Nakusp	1	0
	Nelson	2	0
	Penticton	0	2
	Princeton	1	0
	Revelstoke	1	0
	Salmon Arm	2	0
	Vernon	1	1
Northeast	Blue River	2	1
	Burns Lake	2	0
	Clinton	4	1
	Fort Nelson	2	1
	Fort St. John	2	1
	Mackenzie	2	0
	Prince George	2	1
	Quesnel	3	1
	Smithers	2	0
	Tatlayoko	1	0
	Williams Lake	3	1
Northwest	Dease Lake	0	1
	Terrace	3	3
	Watson Lake	2	0
	Port Hardy	1	0
	Prince Rupert	0	0
	Sandspit	0	1

## Supplementary Materials

The following are available online at http://www.mdpi.com/1660-4601/15/9/2048/s1, Sample code and data for the main threshold analyses, Figure S1: Daily low temperature forecasts (independent variable) and observations (dependent variable) from 2004 to 2016 in 35 forecast areas in British Columbia, Canada, with the Pearson correlation coefficient and slope of the relationship between them, Figure S2: Daily high temperature forecasts (independent variable) and observations (dependent variable) from 2004 to 2016 in 35 forecast areas in British Columbia, Canada, with the Pearson correlation coefficient and slope of the relationship between them, Figure S3: Relative rates and 95% confidence intervals for mortality during Category 0, Category 1, and Category 2 heat compared with Category 3 heat for combinations of high-low-high heat alert thresholds in the Southwest Heat Alert Area, Figure S4: Relative rates and 95% confidence intervals for mortality during Category 0, Category 1, and Category 2 heat compared with Category 3 heat for combinations of high-low-high heat alert thresholds in the Southeast Heat Alert Area, Figure S5: Relative rates and 95% confidence intervals for mortality during Category 0, Category 1, and Category 2 heat compared with Category 3 heat for combinations of high-low-high heat alert thresholds in the Northeast Heat Alert Area, Figure S6: Relative rates and 95% confidence intervals for mortality during Category 0, Category 1, and Category 2 heat compared with Category 3 heat for combinations of high-low-high heat alert thresholds in the Northwest Heat Alert Area, Figure S7: Retrospective heat alerts based on forecast and observed temperatures in the Southwest Health Alert Area from 2004 to 2016, Figure S8: Retrospective heat alerts based on forecast and observed temperatures in the Southeast Health Alert Area from 2004 to 2016, Figure S9: Retrospective heat alerts based on forecast and observed temperatures in the Northeast Health Alert Area from 2004 to 2016, Figure S10: Retrospective heat alerts based on forecast and observed temperatures in the Northwest Health Alert Area from 2004 to 2016.
