# Supplementary material for: Establishing Heat Alert Thresholds for the Varied Climatic Regions of British Columbia, Canada
Source: Int J Environ Res Public Health. 2018 Sep 19;15(9):2048. doi: 10.3390/ijerph15092048 (PMC6163932; doi:10.3390/ijerph15092048)
Supplement: Supplementary file 1 [file ijerph-15-02048-s001.zip › ijerph-343571-Supplementary materials-proofreading/ijerph-343571-Supplementary materials-proofreading/S10_HeatAlertsPerYear_Northwest.pdf]

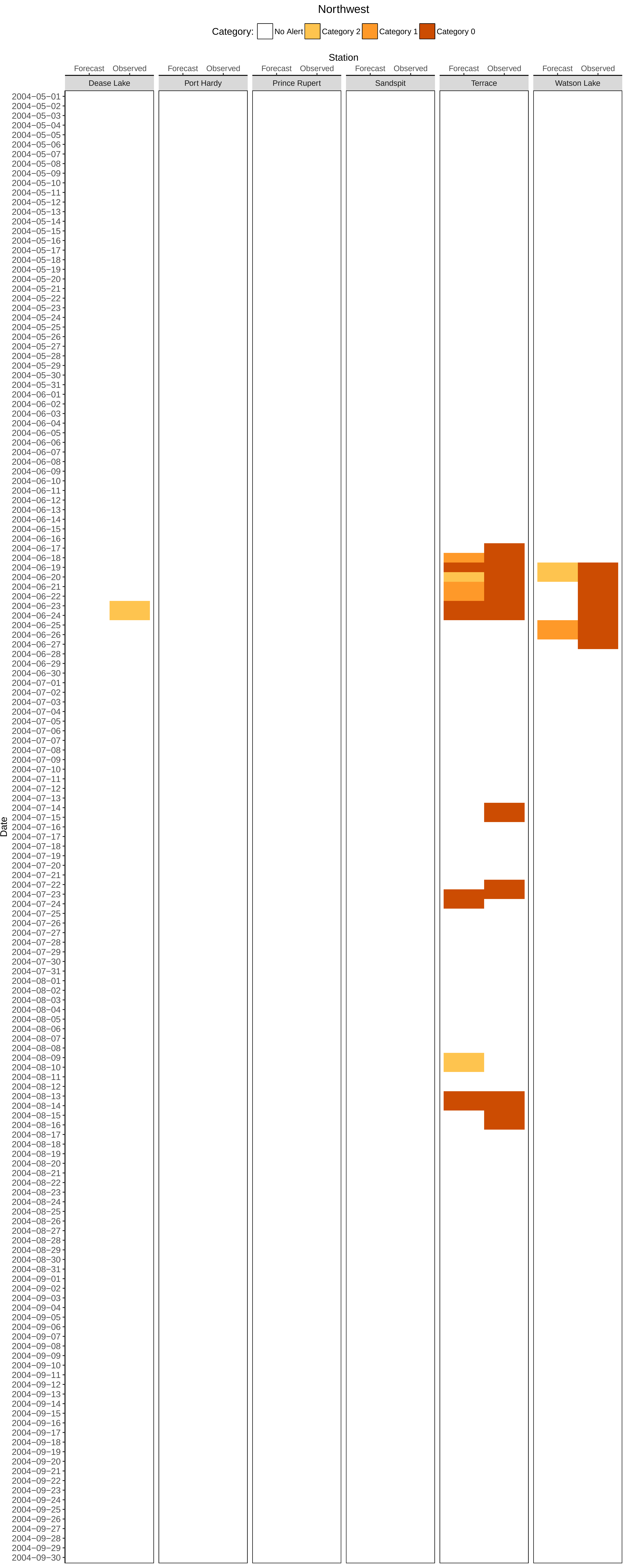
Northwest
Category:
No Alert
Category 2
Category 1
Category 0
Station
Forecast
Observed
Dease Lake
Port Hardy
Prince Rupert
Sandspit
Terrace
Watson Lake
Date
2004−05−01
2004−05−02
2004−05−03
2004−05−04
2004−05−05
2004−05−06
2004−05−07
2004−05−08
2004−05−09
2004−05−10
2004−05−11
2004−05−12
2004−05−13
2004−05−14
2004−05−15
2004−05−16
2004−05−17
2004−05−18
2004−05−19
2004−05−20
2004−05−21
2004−05−22
2004−05−23
2004−05−24
2004−05−25
2004−05−26
2004−05−27
2004−05−28
2004−05−29
2004−05−30
2004−05−31
2004−06−01
2004−06−02
2004−06−03
2004−06−04
2004−06−05
2004−06−06
2004−06−07
2004−06−08
2004−06−09
2004−06−10
2004−06−11
2004−06−12
2004−06−13
2004−06−14
2004−06−15
2004−06−16
2004−06−17
2004−06−18
2004−06−19
2004−06−20
2004−06−21
2004−06−22
2004−06−23
2004−06−24
2004−06−25
2004−06−26
2004−06−27
2004−06−28
2004−06−29
2004−06−30
2004−07−01
2004−07−02
2004−07−03
2004−07−04
2004−07−05
2004−07−06
2004−07−07
2004−07−08
2004−07−09
2004−07−10
2004−07−11
2004−07−12
2004−07−13
2004−07−14
2004−07−15
2004−07−16
2004−07−17
2004−07−18
2004−07−19
2004−07−20
2004−07−21
2004−07−22
2004−07−23
2004−07−24
2004−07−25
2004−07−26
2004−07−27
2004−07−28
2004−07−29
2004−07−30
2004−07−31
2004−08−01
2004−08−02
2004−08−03
2004−08−04
2004−08−05
2004−08−06
2004−08−07
2004−08−08
2004−08−09
2004−08−10
2004−08−11
2004−08−12
2004−08−13
2004−08−14
2004−08−15
2004−08−16
2004−08−17
2004−08−18
2004−08−19
2004−08−20
2004−08−21
2004−08−22
2004−08−23
2004−08−24
2004−08−25
2004−08−26
2004−08−27
2004−08−28
2004−08−29
2004−08−30
2004−08−31
2004−09−01
2004−09−02
2004−09−03
2004−09−04
2004−09−05
2004−09−06
2004−09−07
2004−09−08
2004−09−09
2004−09−10
2004−09−11
2004−09−12
2004−09−13
2004−09−14
2004−09−15
2004−09−16
2004−09−17
2004−09−18
2004−09−19
2004−09−20
2004−09−21
2004−09−22
2004−09−23
2004−09−24
2004−09−25
2004−09−26
2004−09−27
2004−09−28
2004−09−29
2004−09−30

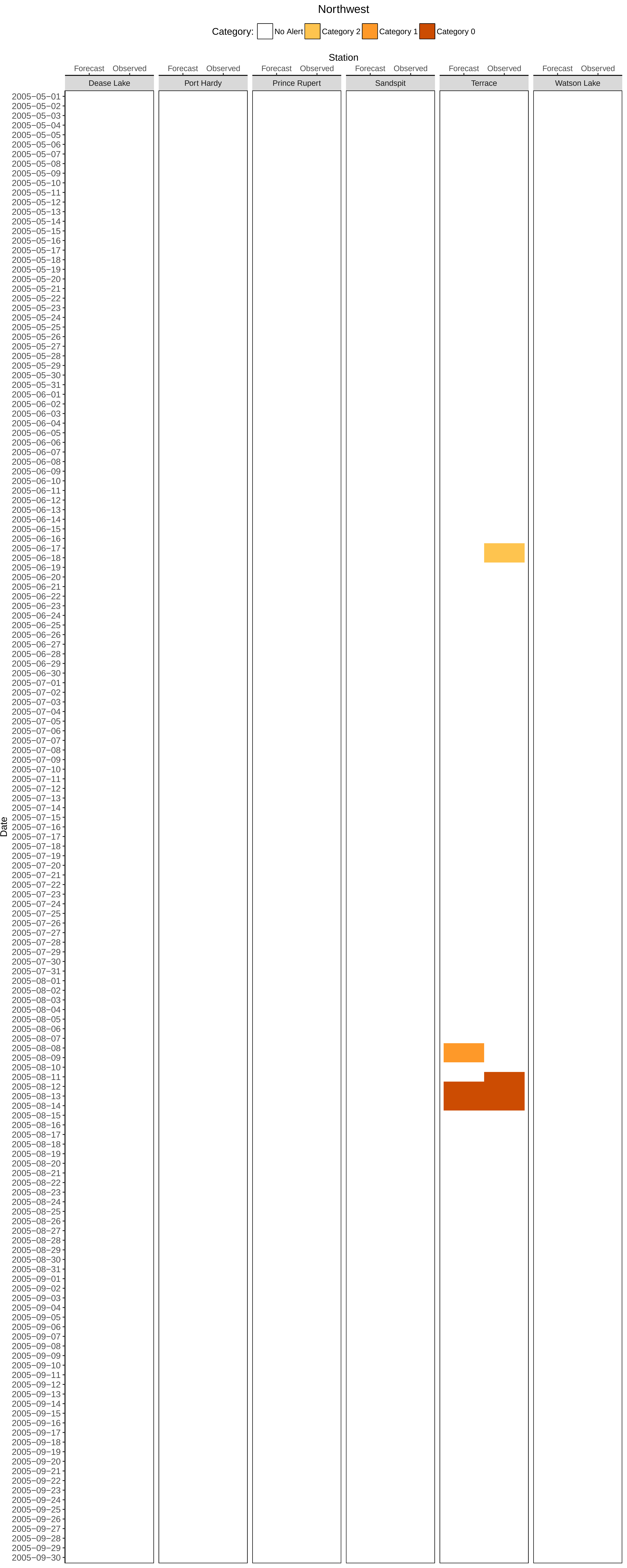
Northwest
Category:
No Alert
Category 2
Category 1
Category 0
Station
Forecast
Observed
Dease Lake
Port Hardy
Prince Rupert
Sandspit
Terrace
Watson Lake
Date
2005−05−01
2005−09−30

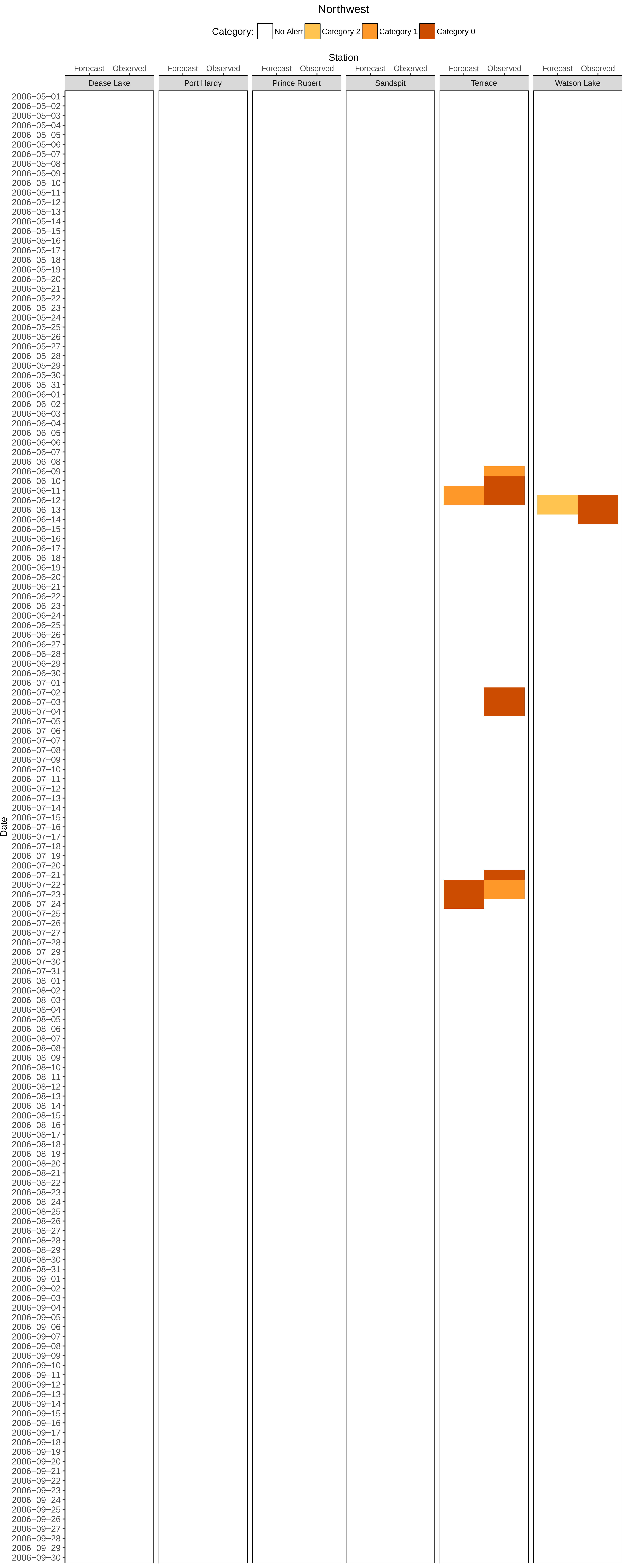

Northwest
Category:
No Alert
Category 2
Category 1
Category 0
Station
Forecast
Observed
Dease Lake
Port Hardy
Prince Rupert
Sandspit
Terrace
Watson Lake
Date
2006−05−01
2006−09−30

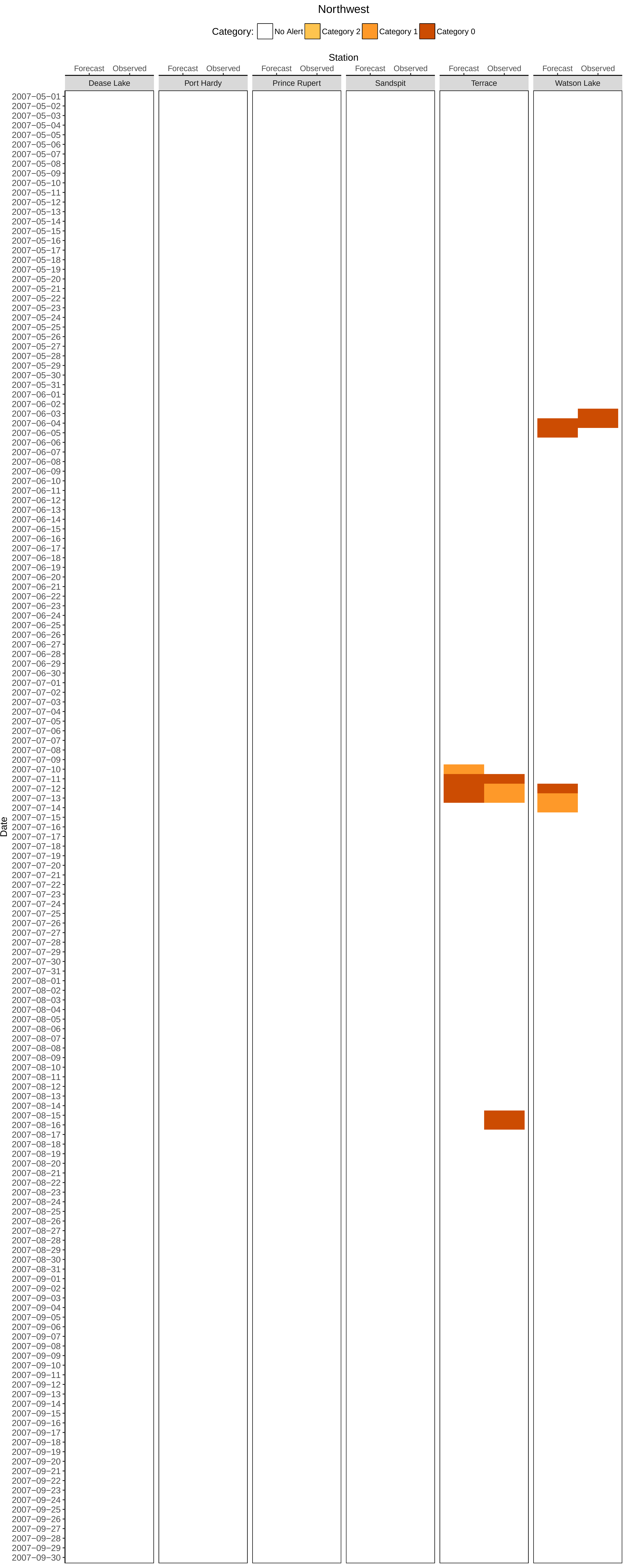
Northwest
Category:
No Alert
Category 2
Category 1
Category 0
Station
Forecast
Observed
Dease Lake
Port Hardy
Prince Rupert
Sandspit
Terrace
Watson Lake
Date
2007−05−01
2007−09−30

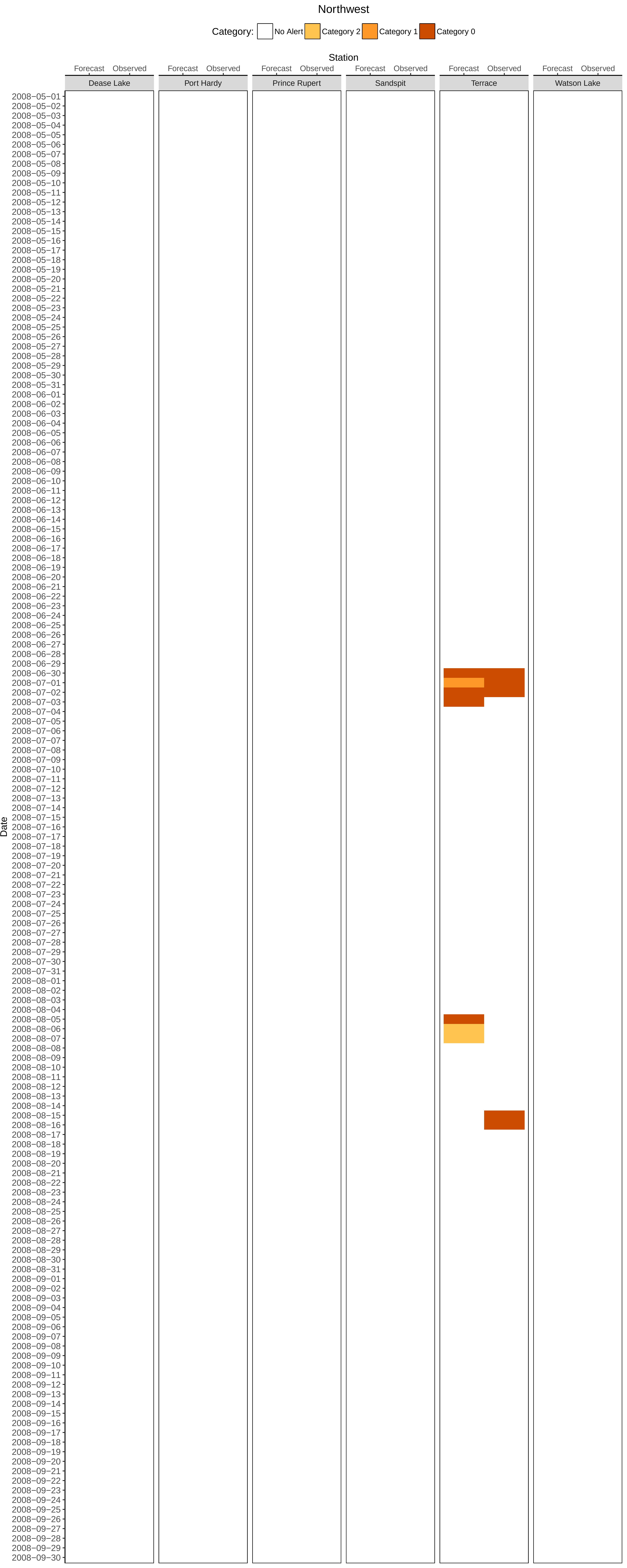
Northwest
Category:
No Alert
Category 2
Category 1
Category 0
Station
Forecast
Observed
Dease Lake
Port Hardy
Prince Rupert
Sandspit
Terrace
Watson Lake
Date
2008−05−01
2008−09−30

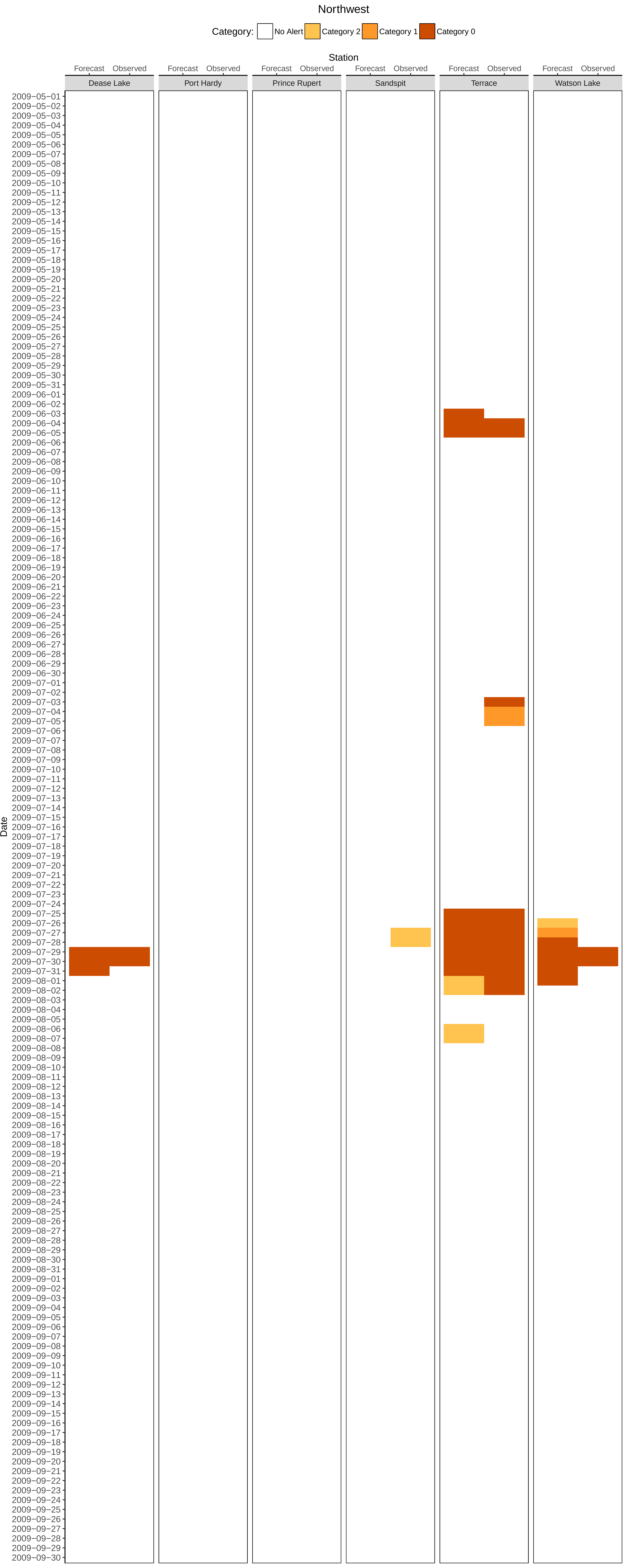
Northwest
Category:
No Alert
Category 2
Category 1
Category 0
Station
Forecast
Observed
Dease Lake
Port Hardy
Prince Rupert
Sandspit
Terrace
Watson Lake
Date
2009−05−01
2009−09−30

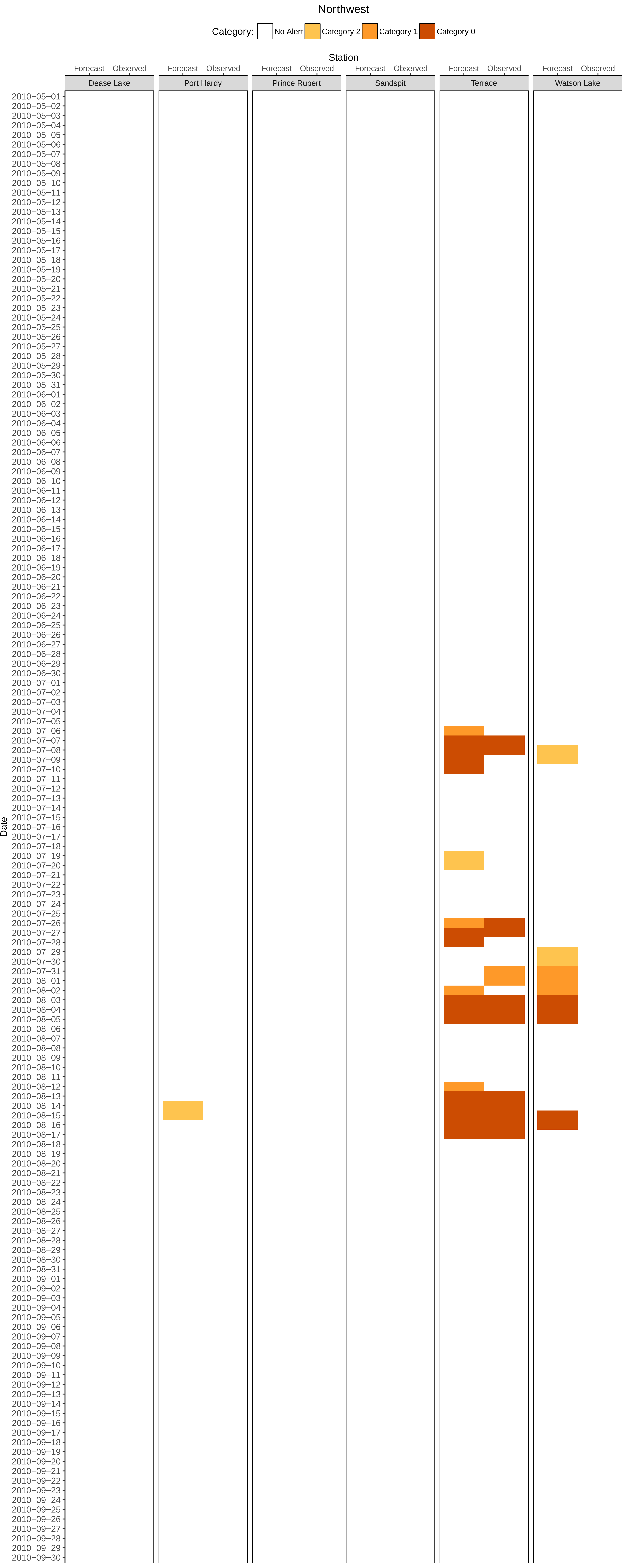

Northwest
Category: No Alert Category 2 Category 1 Category 0
Station
Forecast Observed
Dease Lake
Port Hardy
Prince Rupert
Sandspit
Terrace
Watson Lake
Date
2010−05−01 … 2010−09−30

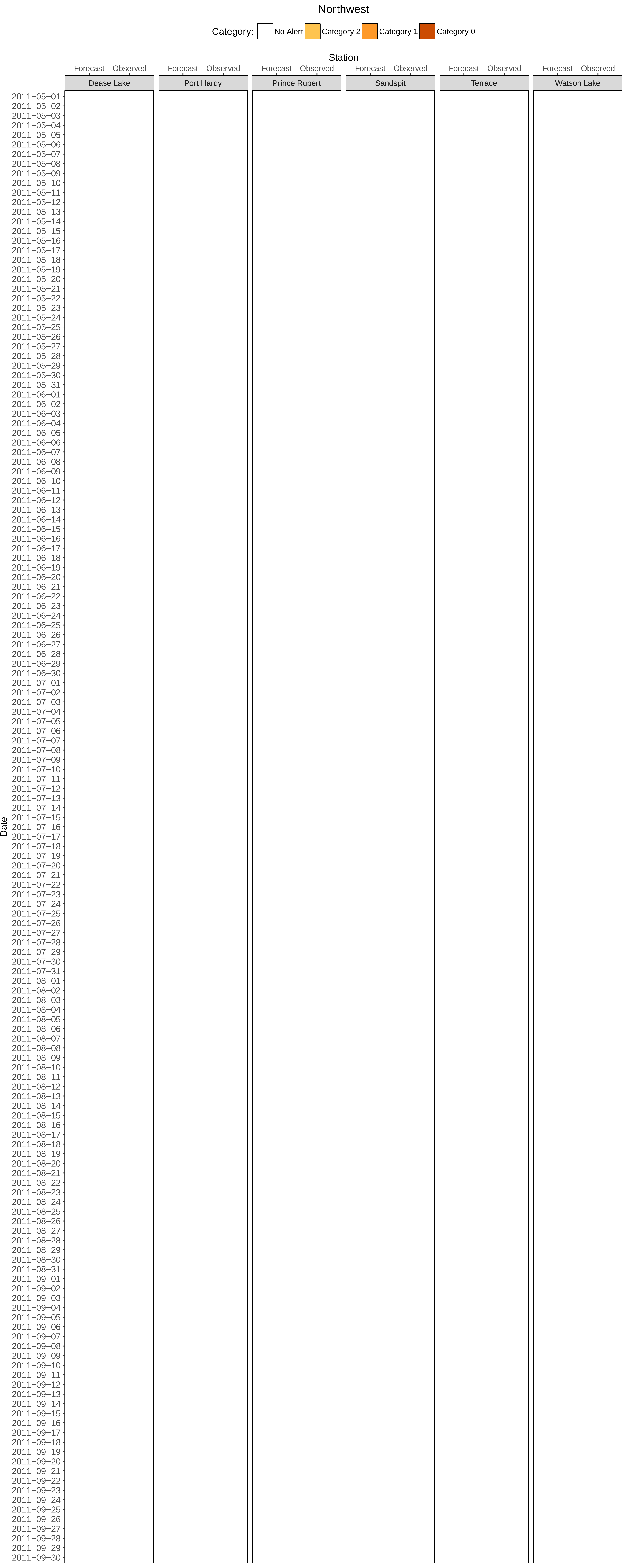
Northwest
Category:
No Alert
Category 2
Category 1
Category 0
Station
Forecast
Observed
Dease Lake
Port Hardy
Prince Rupert
Sandspit
Terrace
Watson Lake
Date
2011−05−01
2011−05−02
2011−05−03
2011−05−04
2011−05−05
2011−05−06
2011−05−07
2011−05−08
2011−05−09
2011−05−10
2011−05−11
2011−05−12
2011−05−13
2011−05−14
2011−05−15
2011−05−16
2011−05−17
2011−05−18
2011−05−19
2011−05−20
2011−05−21
2011−05−22
2011−05−23
2011−05−24
2011−05−25
2011−05−26
2011−05−27
2011−05−28
2011−05−29
2011−05−30
2011−05−31
2011−06−01
2011−06−02
2011−06−03
2011−06−04
2011−06−05
2011−06−06
2011−06−07
2011−06−08
2011−06−09
2011−06−10
2011−06−11
2011−06−12
2011−06−13
2011−06−14
2011−06−15
2011−06−16
2011−06−17
2011−06−18
2011−06−19
2011−06−20
2011−06−21
2011−06−22
2011−06−23
2011−06−24
2011−06−25
2011−06−26
2011−06−27
2011−06−28
2011−06−29
2011−06−30
2011−07−01
2011−07−02
2011−07−03
2011−07−04
2011−07−05
2011−07−06
2011−07−07
2011−07−08
2011−07−09
2011−07−10
2011−07−11
2011−07−12
2011−07−13
2011−07−14
2011−07−15
2011−07−16
2011−07−17
2011−07−18
2011−07−19
2011−07−20
2011−07−21
2011−07−22
2011−07−23
2011−07−24
2011−07−25
2011−07−26
2011−07−27
2011−07−28
2011−07−29
2011−07−30
2011−07−31
2011−08−01
2011−08−02
2011−08−03
2011−08−04
2011−08−05
2011−08−06
2011−08−07
2011−08−08
2011−08−09
2011−08−10
2011−08−11
2011−08−12
2011−08−13
2011−08−14
2011−08−15
2011−08−16
2011−08−17
2011−08−18
2011−08−19
2011−08−20
2011−08−21
2011−08−22
2011−08−23
2011−08−24
2011−08−25
2011−08−26
2011−08−27
2011−08−28
2011−08−29
2011−08−30
2011−08−31
2011−09−01
2011−09−02
2011−09−03
2011−09−04
2011−09−05
2011−09−06
2011−09−07
2011−09−08
2011−09−09
2011−09−10
2011−09−11
2011−09−12
2011−09−13
2011−09−14
2011−09−15
2011−09−16
2011−09−17
2011−09−18
2011−09−19
2011−09−20
2011−09−21
2011−09−22
2011−09−23
2011−09−24
2011−09−25
2011−09−26
2011−09−27
2011−09−28
2011−09−29
2011−09−30

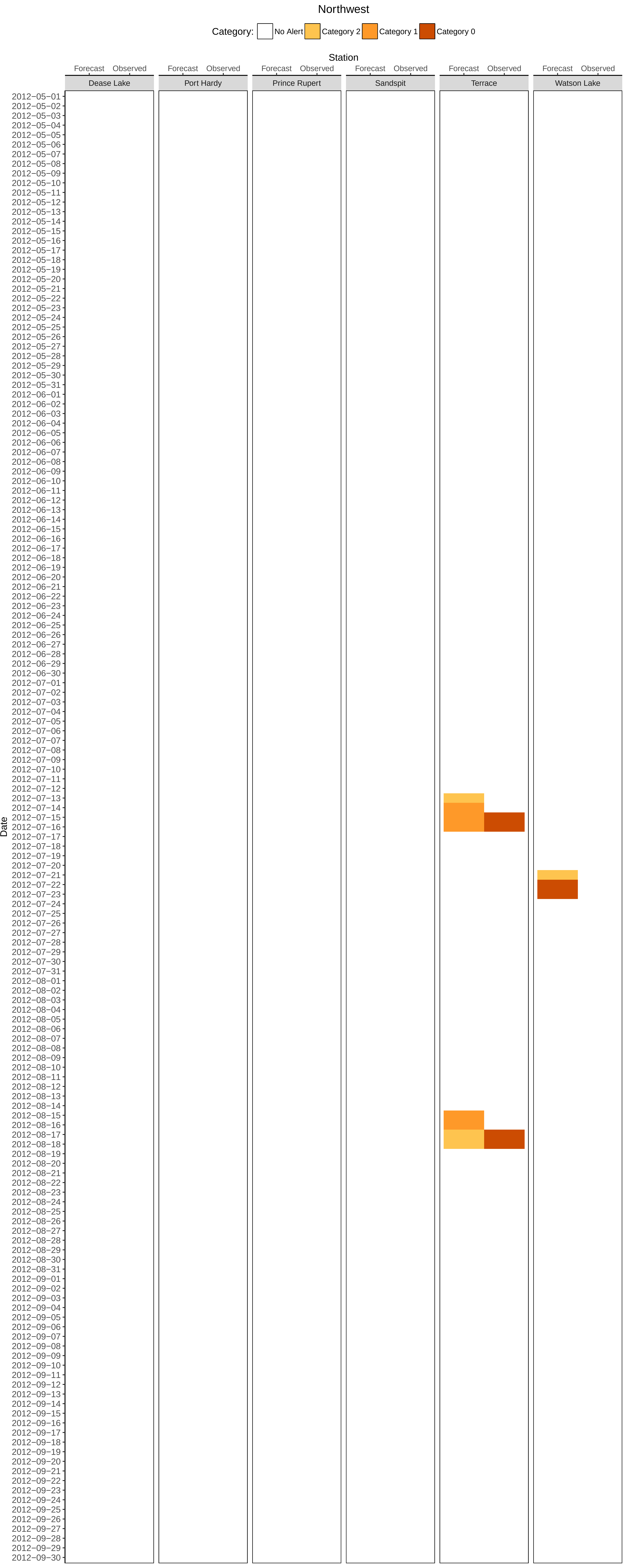

Northwest
Category: No Alert Category 2 Category 1 Category 0
Station
Forecast Observed
Dease Lake
Port Hardy
Prince Rupert
Sandspit
Terrace
Watson Lake
Date
2012-05-01
2012-09-30

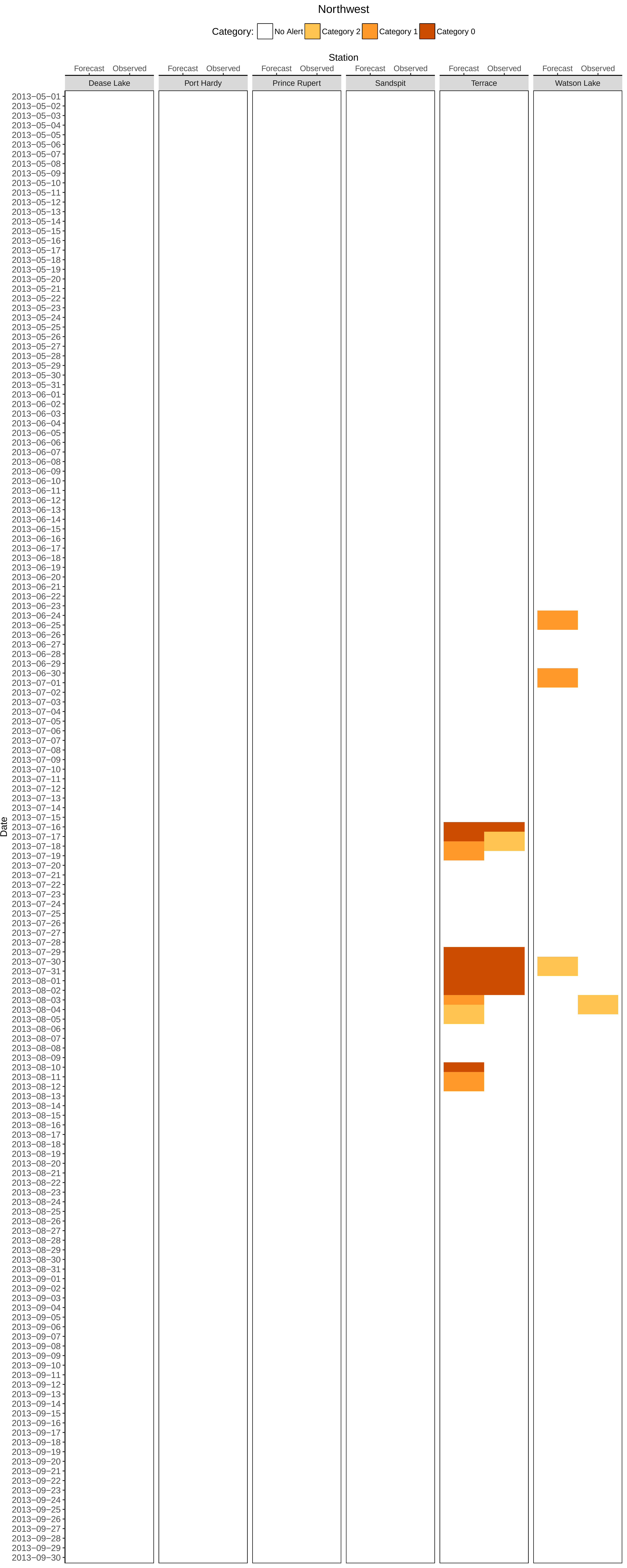

Northwest
Category:
No Alert
Category 2
Category 1
Category 0
Station
Forecast
Observed
Dease Lake
Port Hardy
Prince Rupert
Sandspit
Terrace
Watson Lake
Date
2013−05−01
2013−09−30

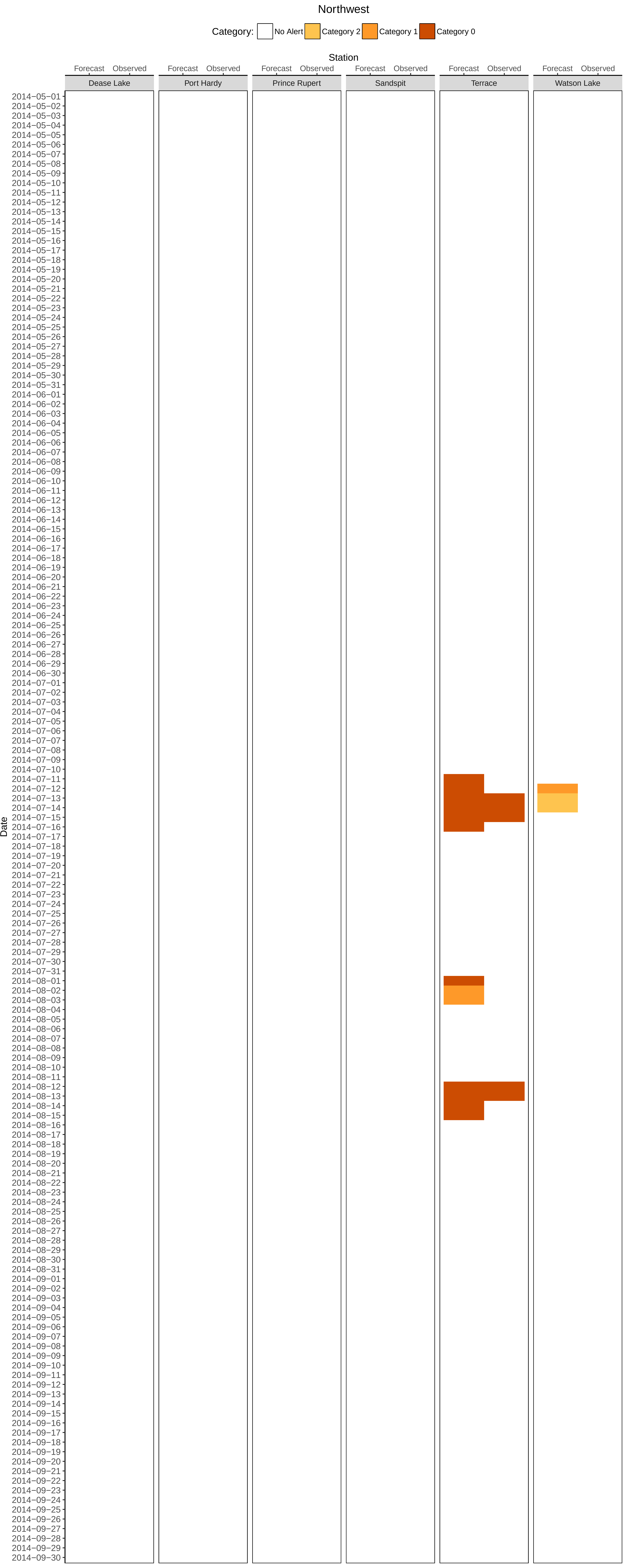
Northwest
Category:
No Alert
Category 2
Category 1
Category 0
Station
Forecast
Observed
Dease Lake
Port Hardy
Prince Rupert
Sandspit
Terrace
Watson Lake
Date
2014−05−01
2014−05−02
2014−05−03
2014−05−04
2014−05−05
2014−05−06
2014−05−07
2014−05−08
2014−05−09
2014−05−10
2014−05−11
2014−05−12
2014−05−13
2014−05−14
2014−05−15
2014−05−16
2014−05−17
2014−05−18
2014−05−19
2014−05−20
2014−05−21
2014−05−22
2014−05−23
2014−05−24
2014−05−25
2014−05−26
2014−05−27
2014−05−28
2014−05−29
2014−05−30
2014−05−31
2014−06−01
2014−06−02
2014−06−03
2014−06−04
2014−06−05
2014−06−06
2014−06−07
2014−06−08
2014−06−09
2014−06−10
2014−06−11
2014−06−12
2014−06−13
2014−06−14
2014−06−15
2014−06−16
2014−06−17
2014−06−18
2014−06−19
2014−06−20
2014−06−21
2014−06−22
2014−06−23
2014−06−24
2014−06−25
2014−06−26
2014−06−27
2014−06−28
2014−06−29
2014−06−30
2014−07−01
2014−07−02
2014−07−03
2014−07−04
2014−07−05
2014−07−06
2014−07−07
2014−07−08
2014−07−09
2014−07−10
2014−07−11
2014−07−12
2014−07−13
2014−07−14
2014−07−15
2014−07−16
2014−07−17
2014−07−18
2014−07−19
2014−07−20
2014−07−21
2014−07−22
2014−07−23
2014−07−24
2014−07−25
2014−07−26
2014−07−27
2014−07−28
2014−07−29
2014−07−30
2014−07−31
2014−08−01
2014−08−02
2014−08−03
2014−08−04
2014−08−05
2014−08−06
2014−08−07
2014−08−08
2014−08−09
2014−08−10
2014−08−11
2014−08−12
2014−08−13
2014−08−14
2014−08−15
2014−08−16
2014−08−17
2014−08−18
2014−08−19
2014−08−20
2014−08−21
2014−08−22
2014−08−23
2014−08−24
2014−08−25
2014−08−26
2014−08−27
2014−08−28
2014−08−29
2014−08−30
2014−08−31
2014−09−01
2014−09−02
2014−09−03
2014−09−04
2014−09−05
2014−09−06
2014−09−07
2014−09−08
2014−09−09
2014−09−10
2014−09−11
2014−09−12
2014−09−13
2014−09−14
2014−09−15
2014−09−16
2014−09−17
2014−09−18
2014−09−19
2014−09−20
2014−09−21
2014−09−22
2014−09−23
2014−09−24
2014−09−25
2014−09−26
2014−09−27
2014−09−28
2014−09−29
2014−09−30

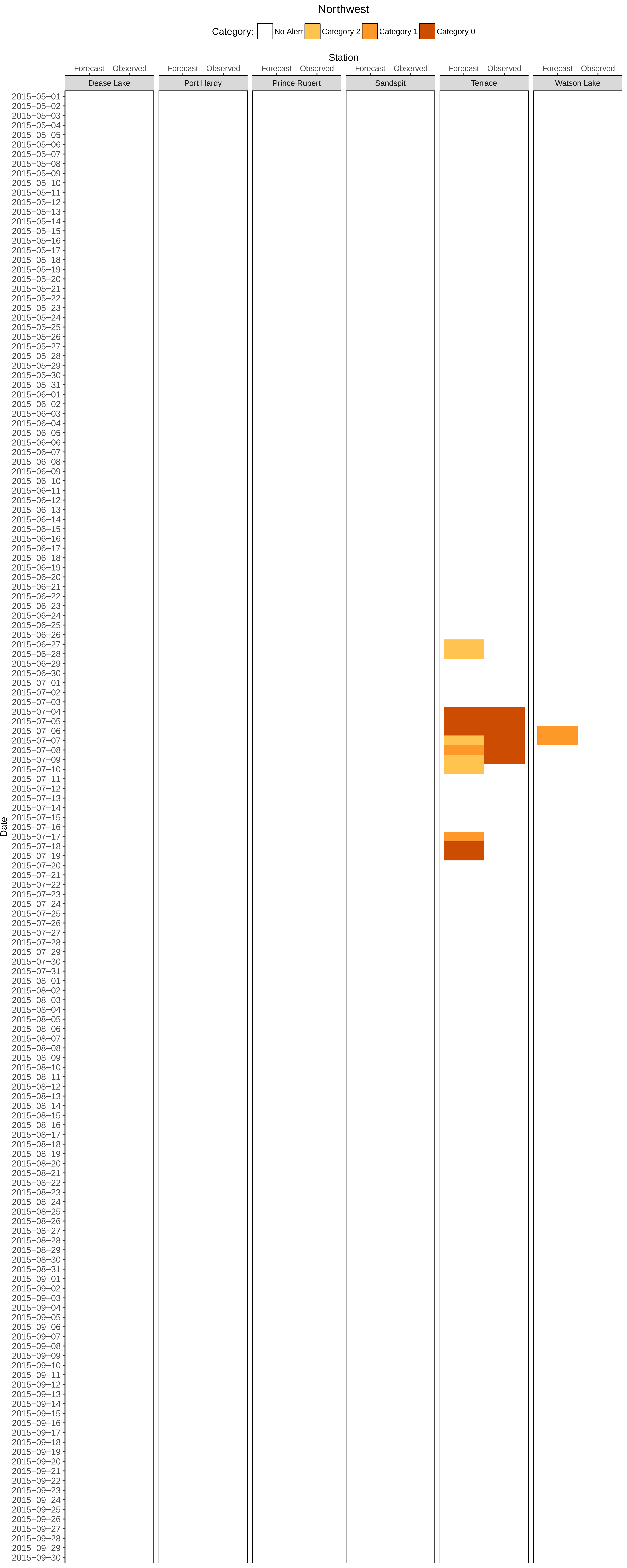

Northwest
Category: No Alert Category 2 Category 1 Category 0
Station
Forecast Observed
Dease Lake
Port Hardy
Prince Rupert
Sandspit
Terrace
Watson Lake
Date
2015−05−01
2015−09−30

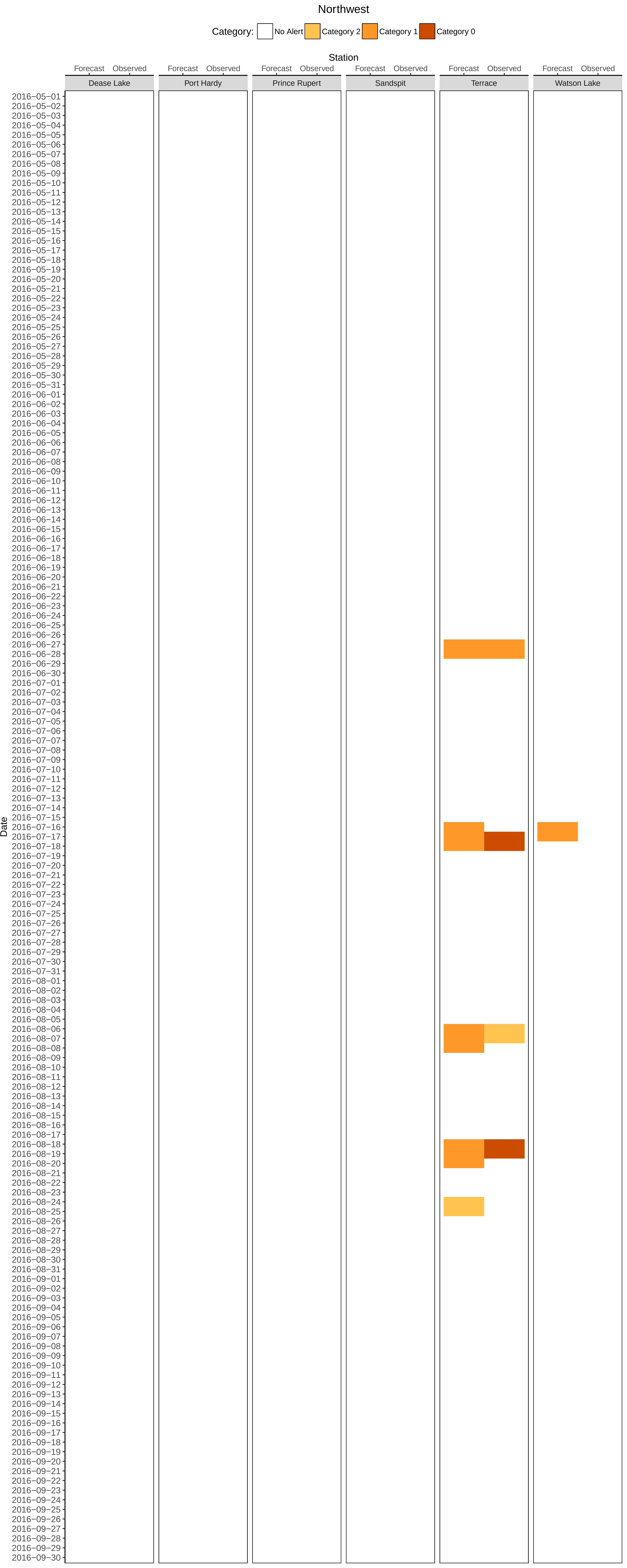

Northwest
Category:
No Alert
Category 2
Category 1
Category 0
Station
Forecast
Observed
Dease Lake
Port Hardy
Prince Rupert
Sandspit
Terrace
Watson Lake
Date
2016−05−01
2016−09−30
