# Supplementary material for: Establishing Heat Alert Thresholds for the Varied Climatic Regions of British Columbia, Canada
Source: Int J Environ Res Public Health. 2018 Sep 19;15(9):2048. doi: 10.3390/ijerph15092048 (PMC6163932; doi:10.3390/ijerph15092048)
Supplement: Supplementary file 1 [file ijerph-15-02048-s001.zip › ijerph-343571-Supplementary materials-proofreading/ijerph-343571-Supplementary materials-proofreading/S7_HeatAlertsPerYear_Southwest.pdf]

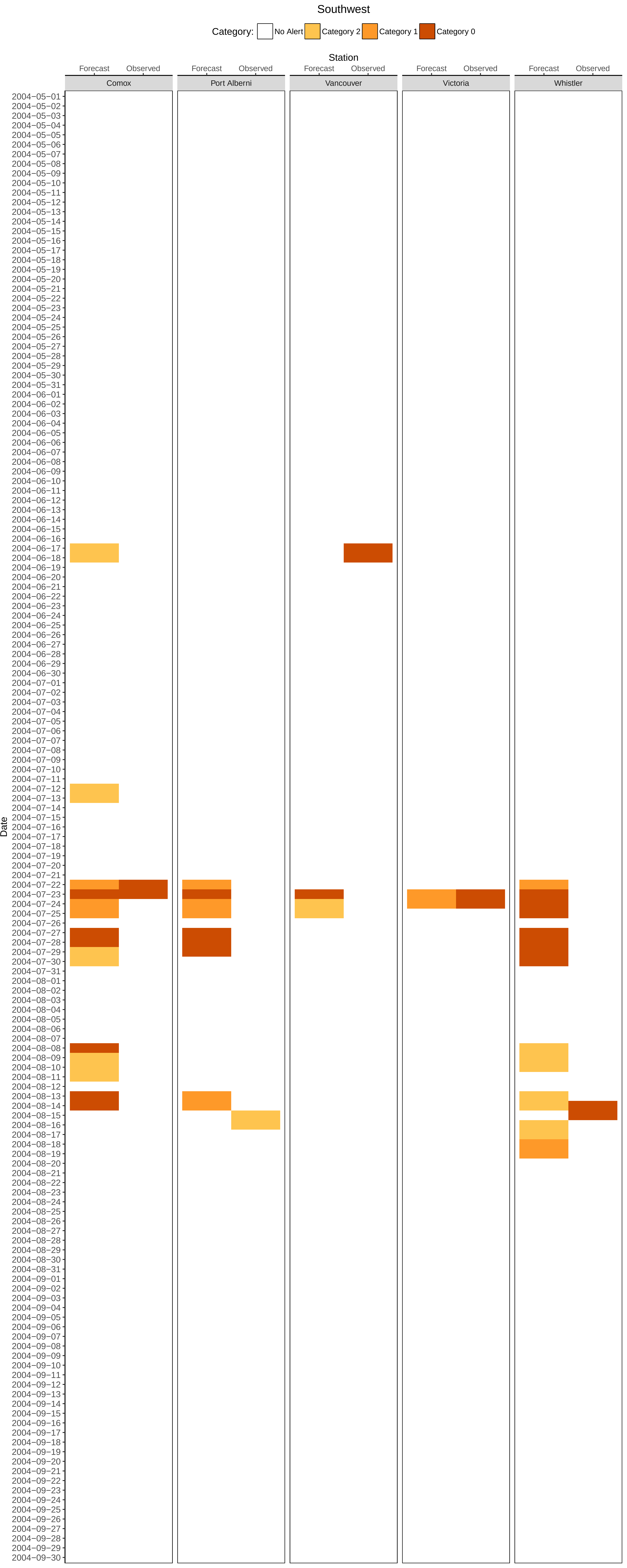
Southwest
Category: No Alert Category 2 Category 1 Category 0
Station
Forecast Observed
Comox
Port Alberni
Vancouver
Victoria
Whistler
Date
2004−05−01
2004−09−30

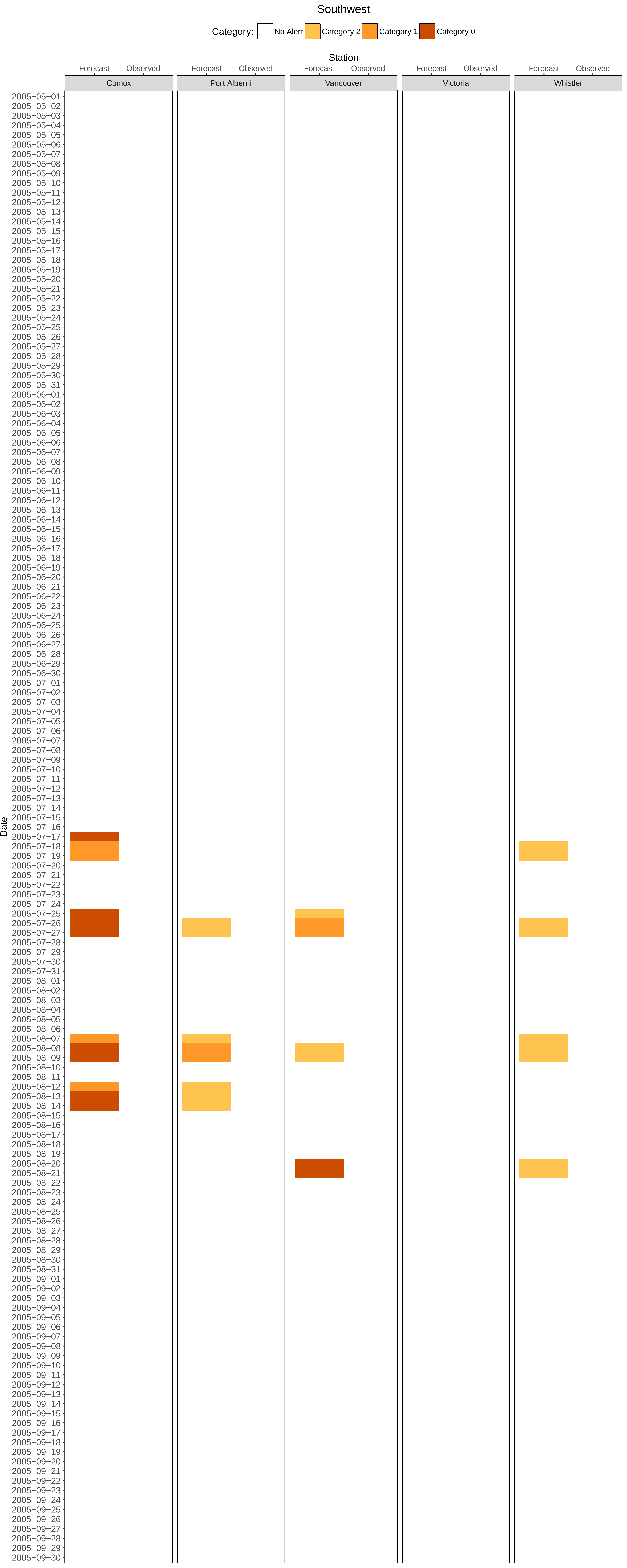
Southwest
Category:
No Alert
Category 2
Category 1
Category 0
Station
Forecast
Observed
Comox
Port Alberni
Vancouver
Victoria
Whistler
Date
2005−05−01
2005−05−02
2005−05−03
2005−05−04
2005−05−05
2005−05−06
2005−05−07
2005−05−08
2005−05−09
2005−05−10
2005−05−11
2005−05−12
2005−05−13
2005−05−14
2005−05−15
2005−05−16
2005−05−17
2005−05−18
2005−05−19
2005−05−20
2005−05−21
2005−05−22
2005−05−23
2005−05−24
2005−05−25
2005−05−26
2005−05−27
2005−05−28
2005−05−29
2005−05−30
2005−05−31
2005−06−01
2005−06−02
2005−06−03
2005−06−04
2005−06−05
2005−06−06
2005−06−07
2005−06−08
2005−06−09
2005−06−10
2005−06−11
2005−06−12
2005−06−13
2005−06−14
2005−06−15
2005−06−16
2005−06−17
2005−06−18
2005−06−19
2005−06−20
2005−06−21
2005−06−22
2005−06−23
2005−06−24
2005−06−25
2005−06−26
2005−06−27
2005−06−28
2005−06−29
2005−06−30
2005−07−01
2005−07−02
2005−07−03
2005−07−04
2005−07−05
2005−07−06
2005−07−07
2005−07−08
2005−07−09
2005−07−10
2005−07−11
2005−07−12
2005−07−13
2005−07−14
2005−07−15
2005−07−16
2005−07−17
2005−07−18
2005−07−19
2005−07−20
2005−07−21
2005−07−22
2005−07−23
2005−07−24
2005−07−25
2005−07−26
2005−07−27
2005−07−28
2005−07−29
2005−07−30
2005−07−31
2005−08−01
2005−08−02
2005−08−03
2005−08−04
2005−08−05
2005−08−06
2005−08−07
2005−08−08
2005−08−09
2005−08−10
2005−08−11
2005−08−12
2005−08−13
2005−08−14
2005−08−15
2005−08−16
2005−08−17
2005−08−18
2005−08−19
2005−08−20
2005−08−21
2005−08−22
2005−08−23
2005−08−24
2005−08−25
2005−08−26
2005−08−27
2005−08−28
2005−08−29
2005−08−30
2005−08−31
2005−09−01
2005−09−02
2005−09−03
2005−09−04
2005−09−05
2005−09−06
2005−09−07
2005−09−08
2005−09−09
2005−09−10
2005−09−11
2005−09−12
2005−09−13
2005−09−14
2005−09−15
2005−09−16
2005−09−17
2005−09−18
2005−09−19
2005−09−20
2005−09−21
2005−09−22
2005−09−23
2005−09−24
2005−09−25
2005−09−26
2005−09−27
2005−09−28
2005−09−29
2005−09−30

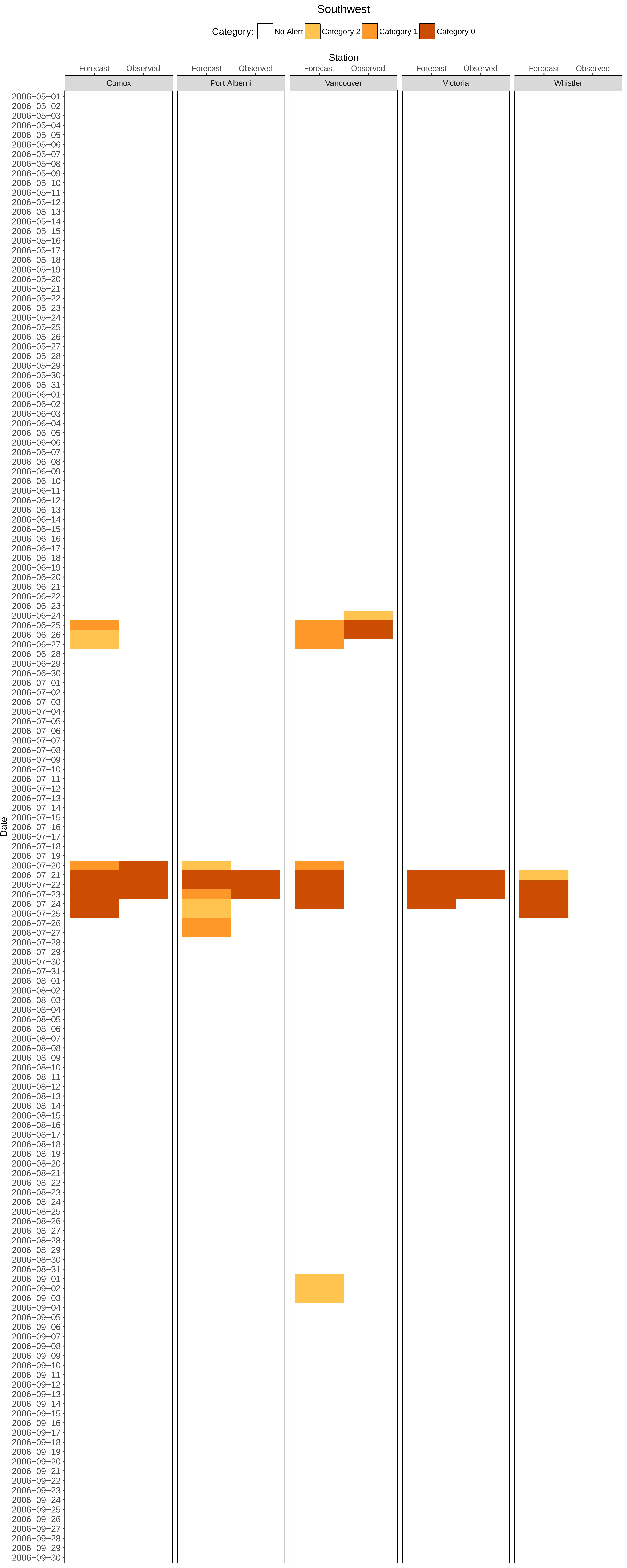
Southwest
Category: No Alert Category 2 Category 1 Category 0
Station
Forecast Observed
Comox
Port Alberni
Vancouver
Victoria
Whistler
Date
2006−05−01 … 2006−09−30

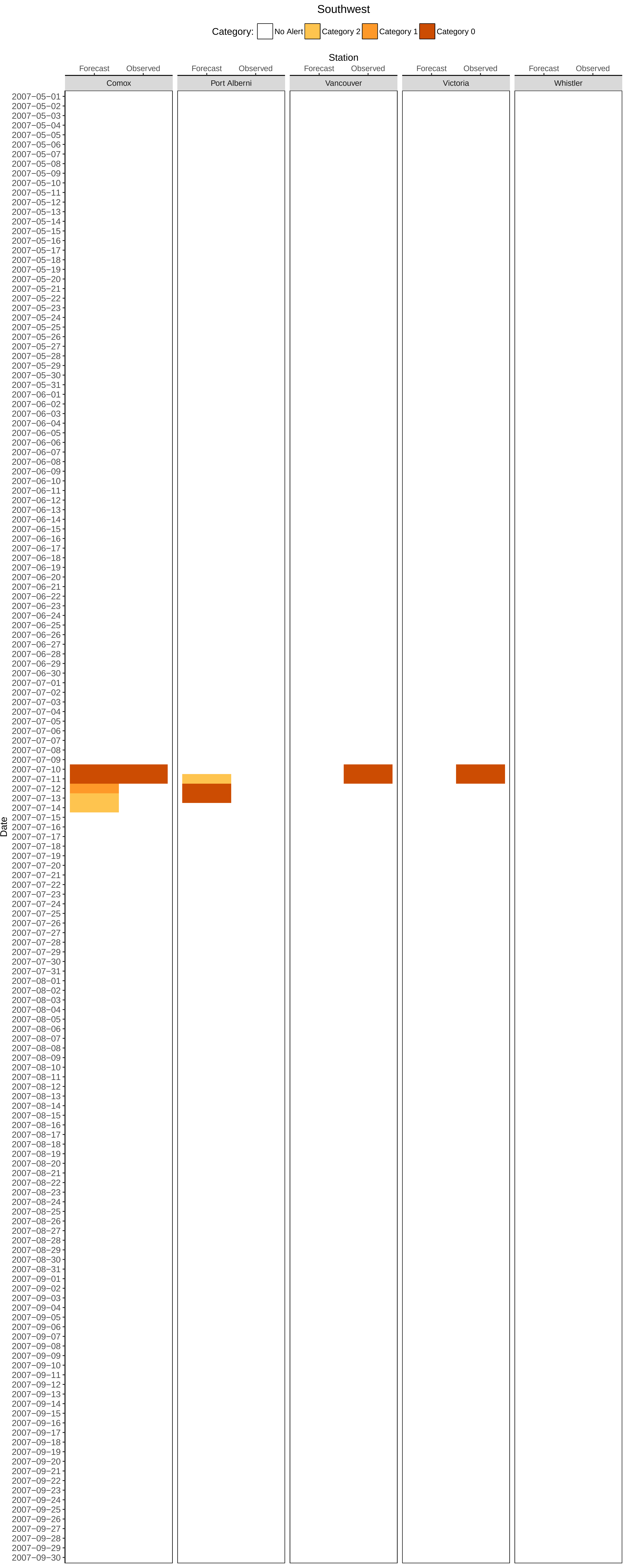
Southwest
Category:
No Alert
Category 2
Category 1
Category 0
Station
Forecast
Observed
Comox
Port Alberni
Vancouver
Victoria
Whistler
Date
2007-05-01
2007-09-30

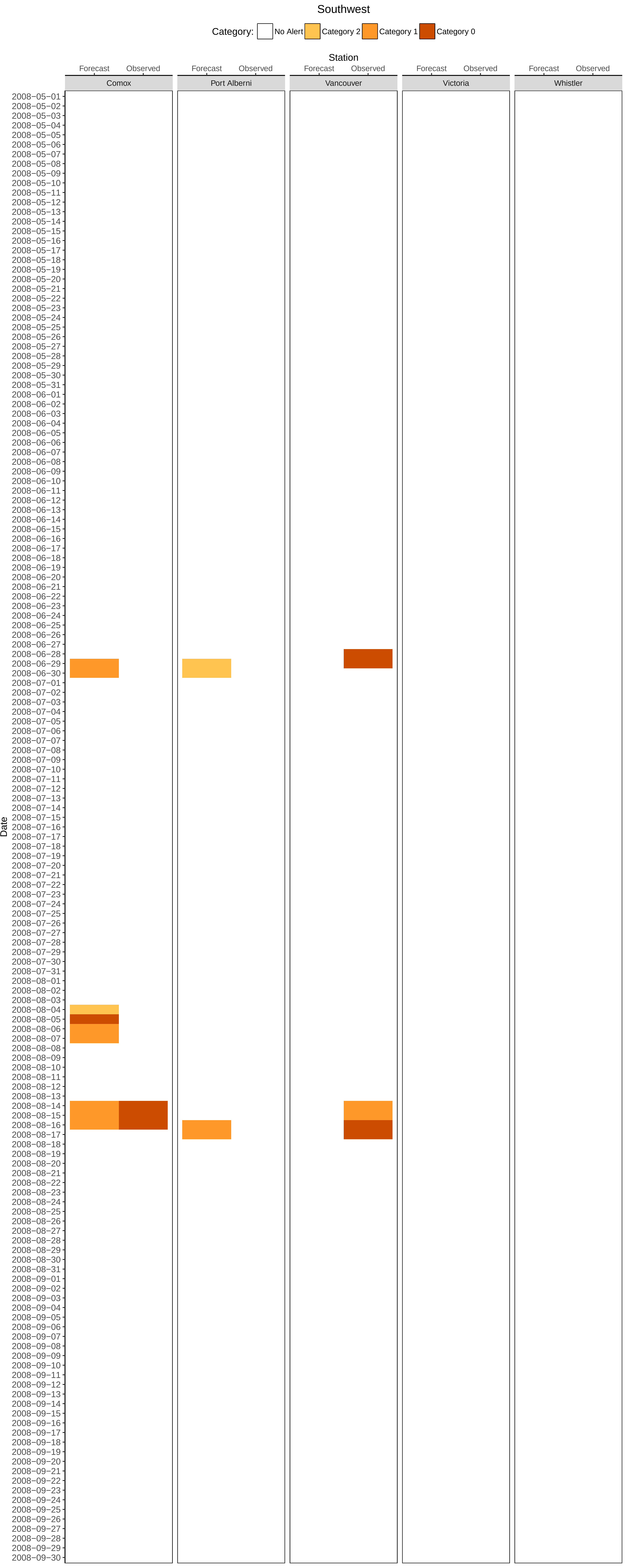
Southwest
Category: No Alert Category 2 Category 1 Category 0
Station
Forecast
Observed
Comox
Port Alberni
Vancouver
Victoria
Whistler
Date
2008−05−01
2008−09−30

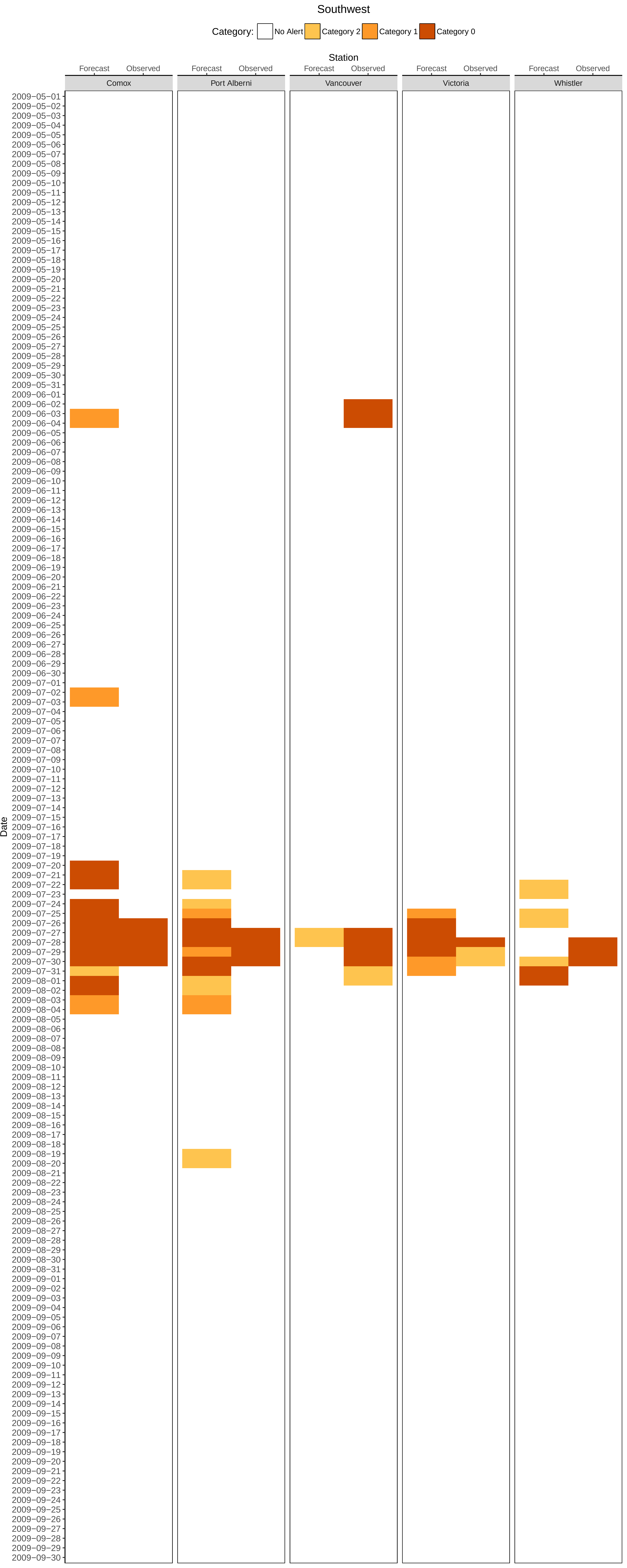
Southwest
Category: No Alert Category 2 Category 1 Category 0
Station
Forecast Observed
Comox
Port Alberni
Vancouver
Victoria
Whistler
Date
2009−05−01
2009−09−30

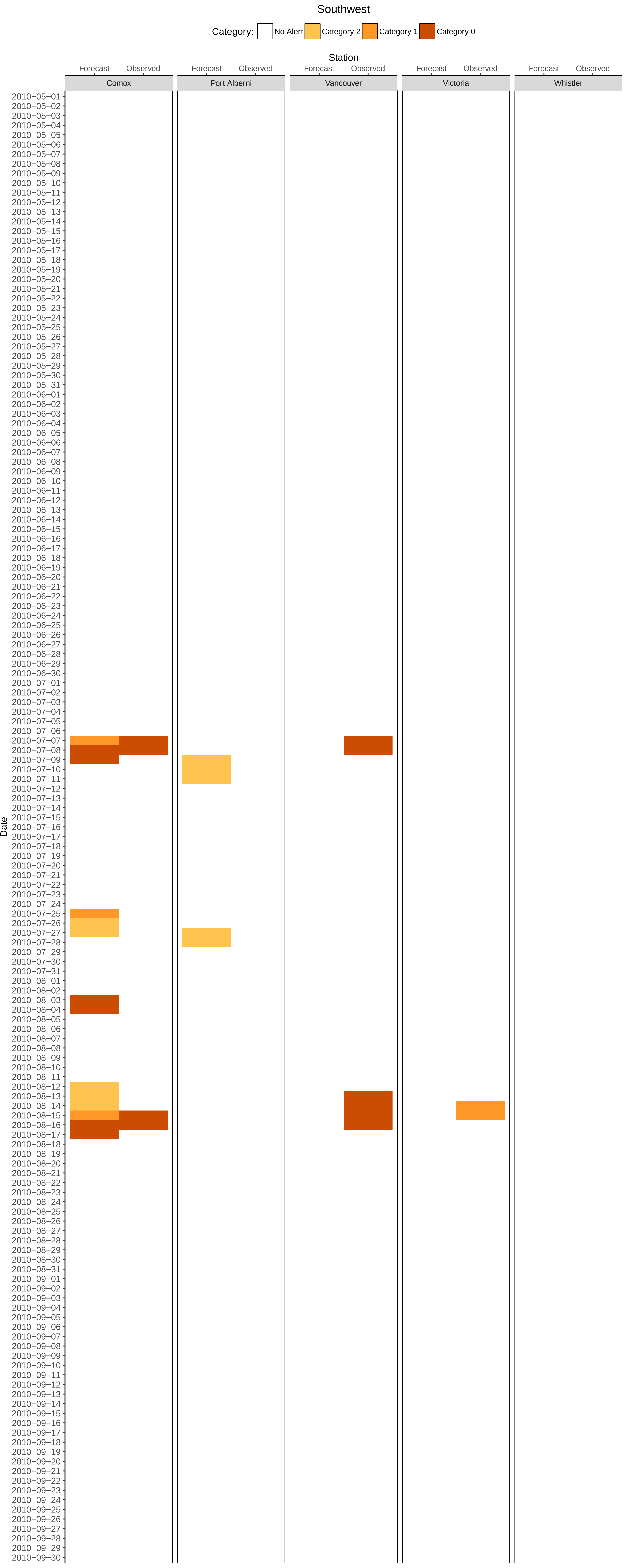
Southwest
Category: No Alert Category 2 Category 1 Category 0
Station
Forecast Observed
Comox
Port Alberni
Vancouver
Victoria
Whistler
Date
2010−05−01
2010−09−30

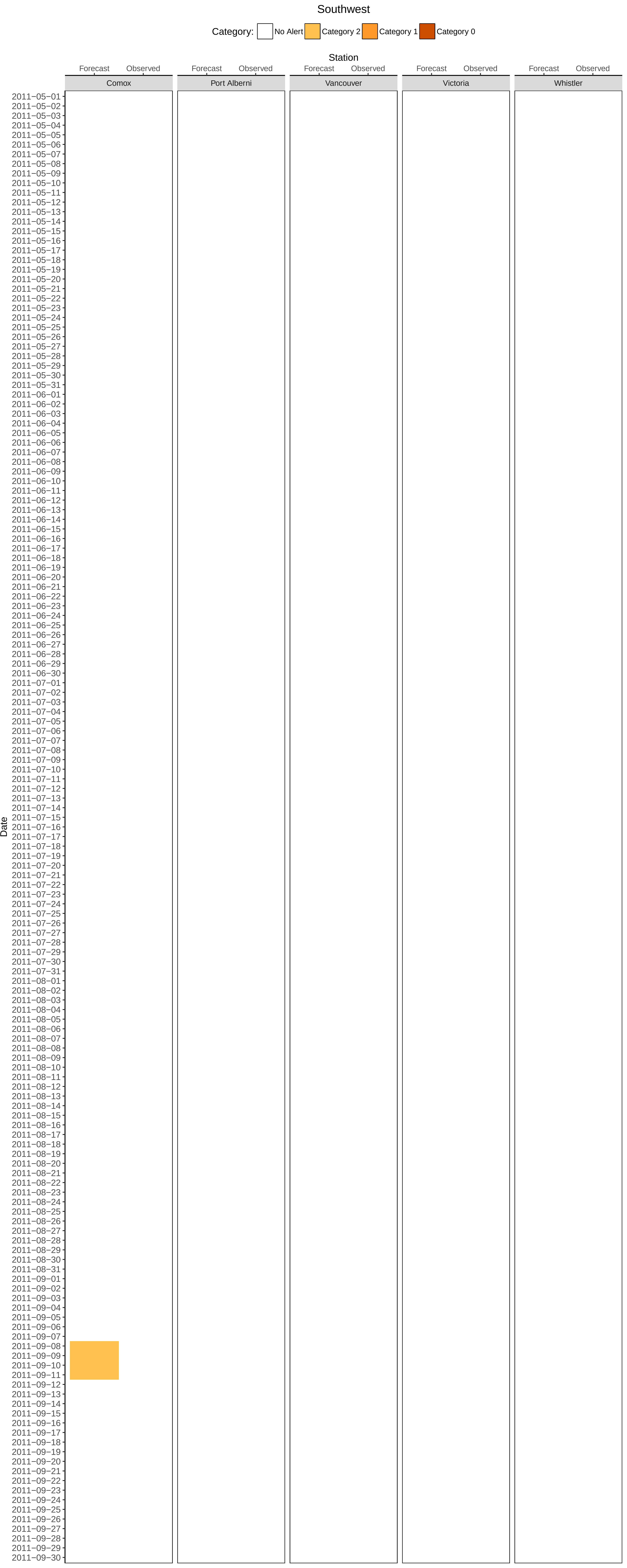

Southwest
Category: No Alert Category 2 Category 1 Category 0
Station
Forecast Observed
Comox
Port Alberni
Vancouver
Victoria
Whistler
Date
2011−05−01
2011−09−30

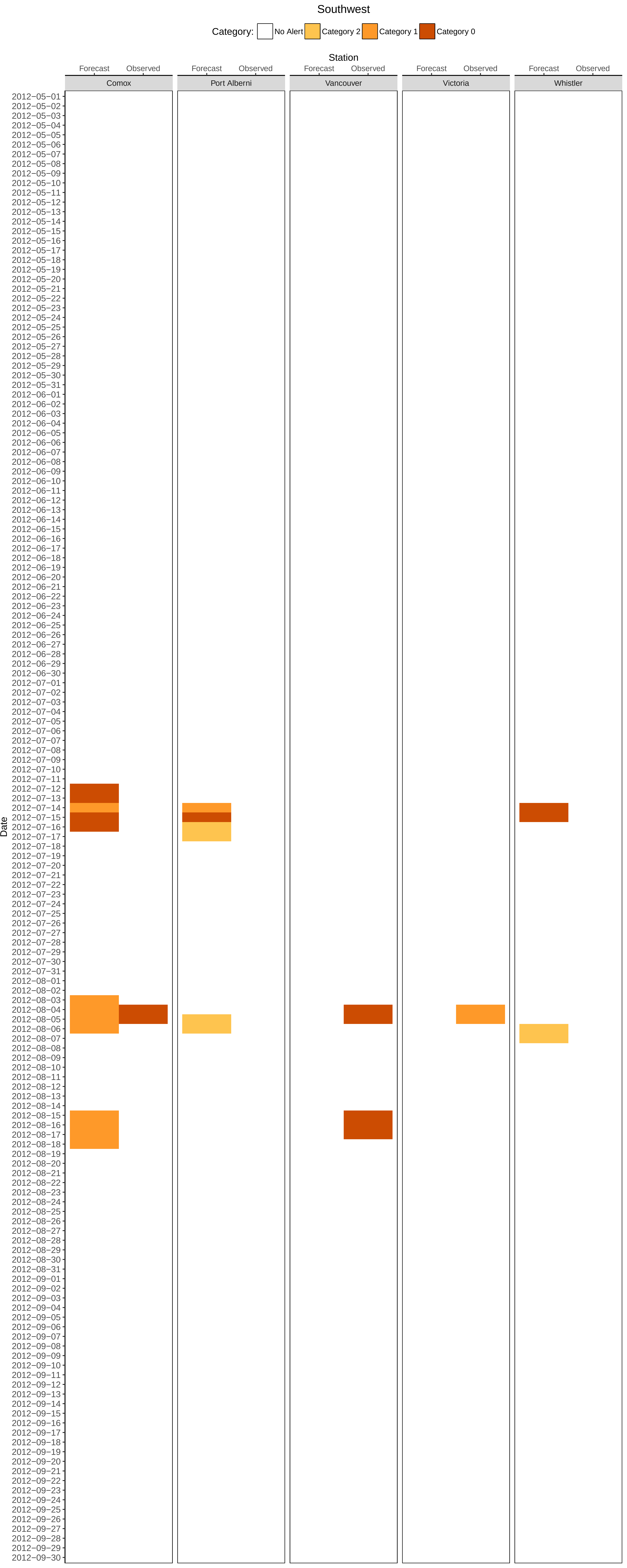
Southwest
Category:
No Alert
Category 2
Category 1
Category 0
Station
Forecast
Observed
Comox
Port Alberni
Vancouver
Victoria
Whistler
Date
2012−05−01
2012−09−30

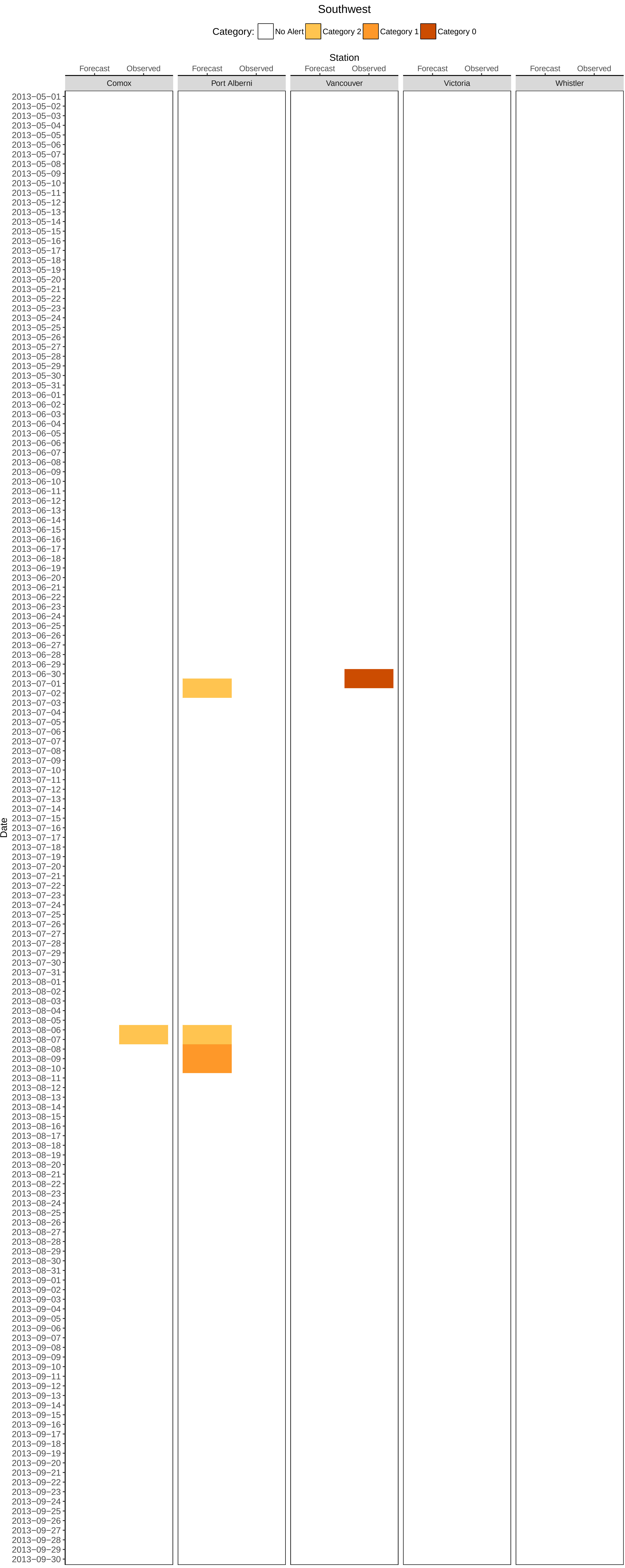
Southwest
Category:
No Alert
Category 2
Category 1
Category 0
Station
Forecast
Observed
Comox
Port Alberni
Vancouver
Victoria
Whistler
Date

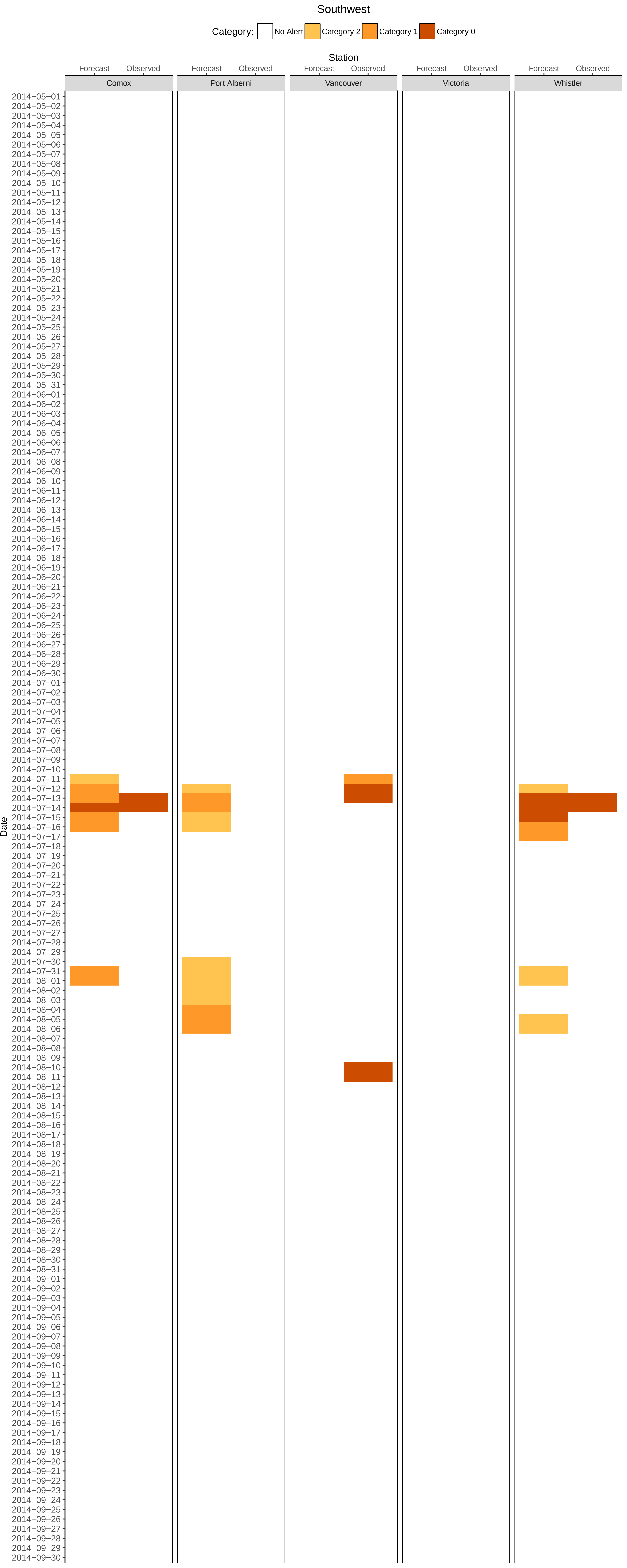
Southwest
Category: No Alert Category 2 Category 1 Category 0
Station
Forecast Observed
Comox
Port Alberni
Vancouver
Victoria
Whistler
Date
2014−05−01
2014−09−30

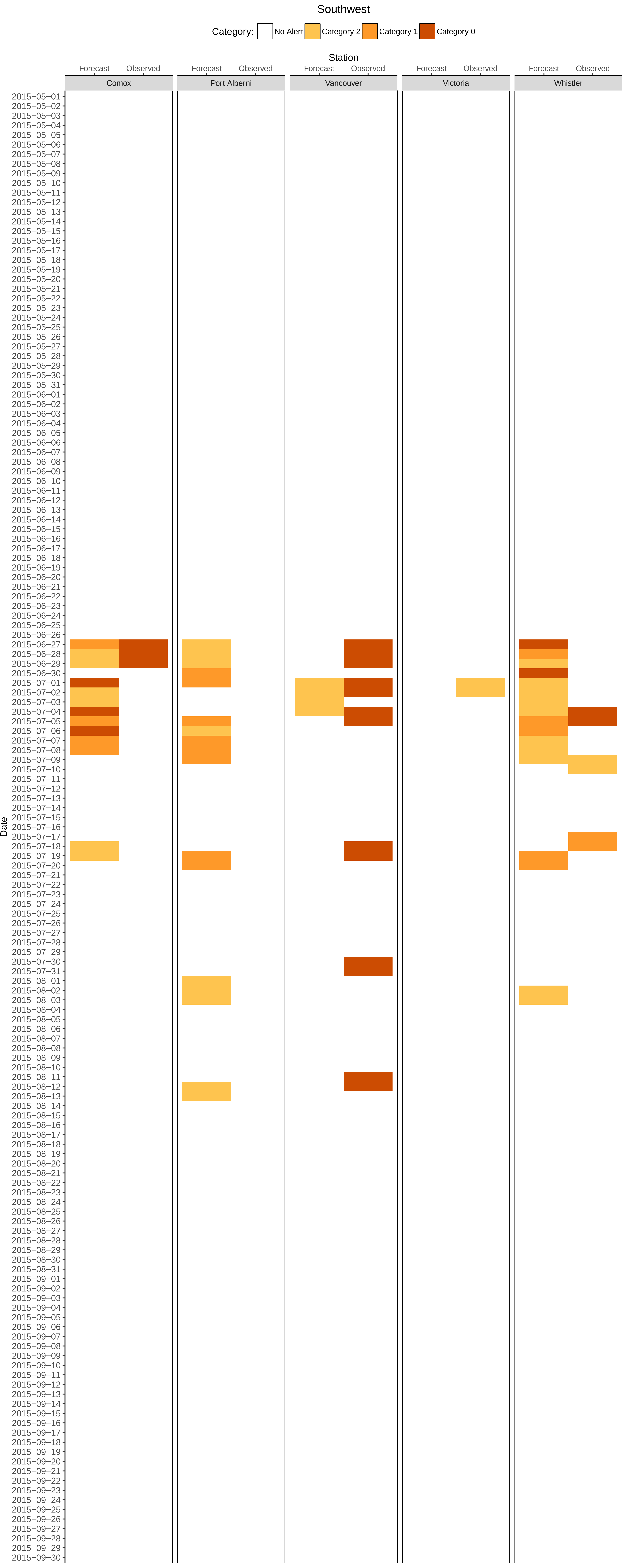

Southwest
Category: No Alert Category 2 Category 1 Category 0
Station
Forecast Observed
Comox
Port Alberni
Vancouver
Victoria
Whistler
Date
2015−05−01
2015−09−30

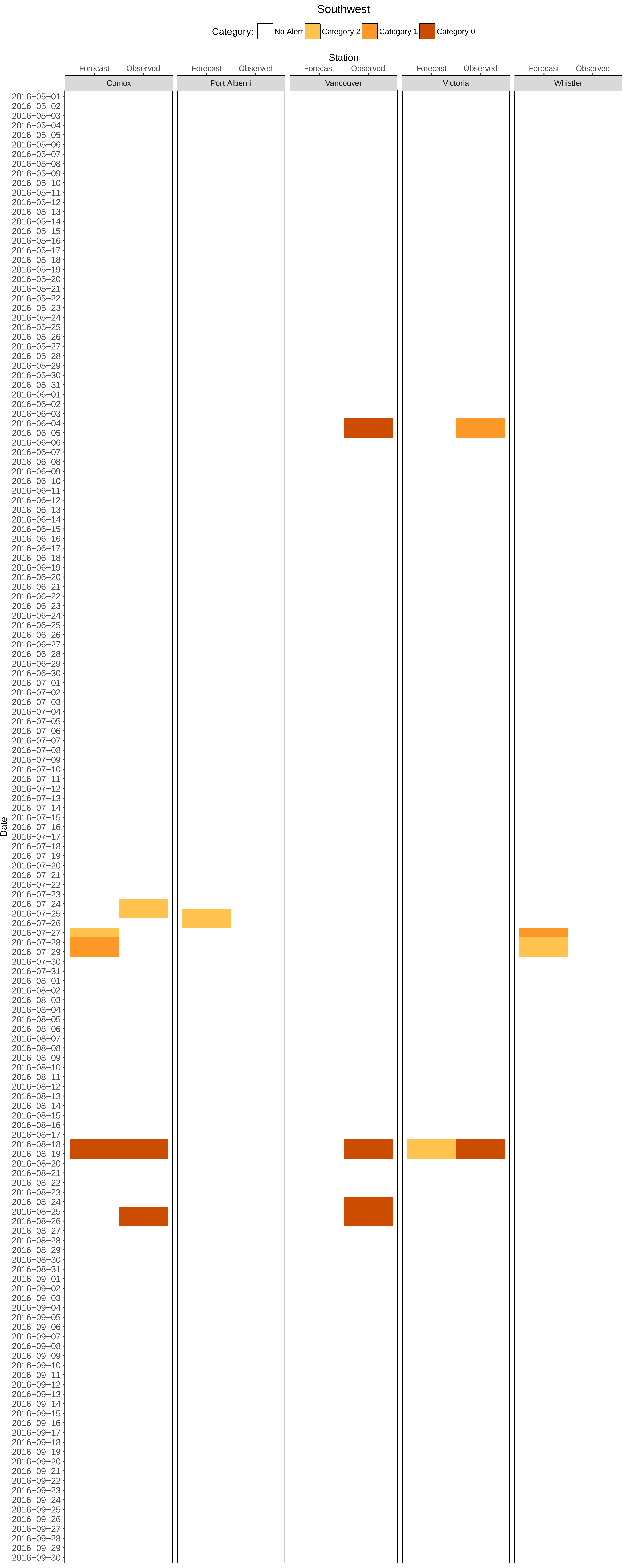

Southwest
Category: No Alert Category 2 Category 1 Category 0
Station
Forecast Observed
Comox
Port Alberni
Vancouver
Victoria
Whistler
Date
2016−05−01
2016−09−30
