# Supplementary material for: Establishing Heat Alert Thresholds for the Varied Climatic Regions of British Columbia, Canada
Source: Int J Environ Res Public Health. 2018 Sep 19;15(9):2048. doi: 10.3390/ijerph15092048 (PMC6163932; doi:10.3390/ijerph15092048)
Supplement: Supplementary file 1 [file ijerph-15-02048-s001.zip › ijerph-343571-Supplementary materials-proofreading/ijerph-343571-Supplementary materials-proofreading/S8_HeatAlertsPerYear_Southeast.pdf]

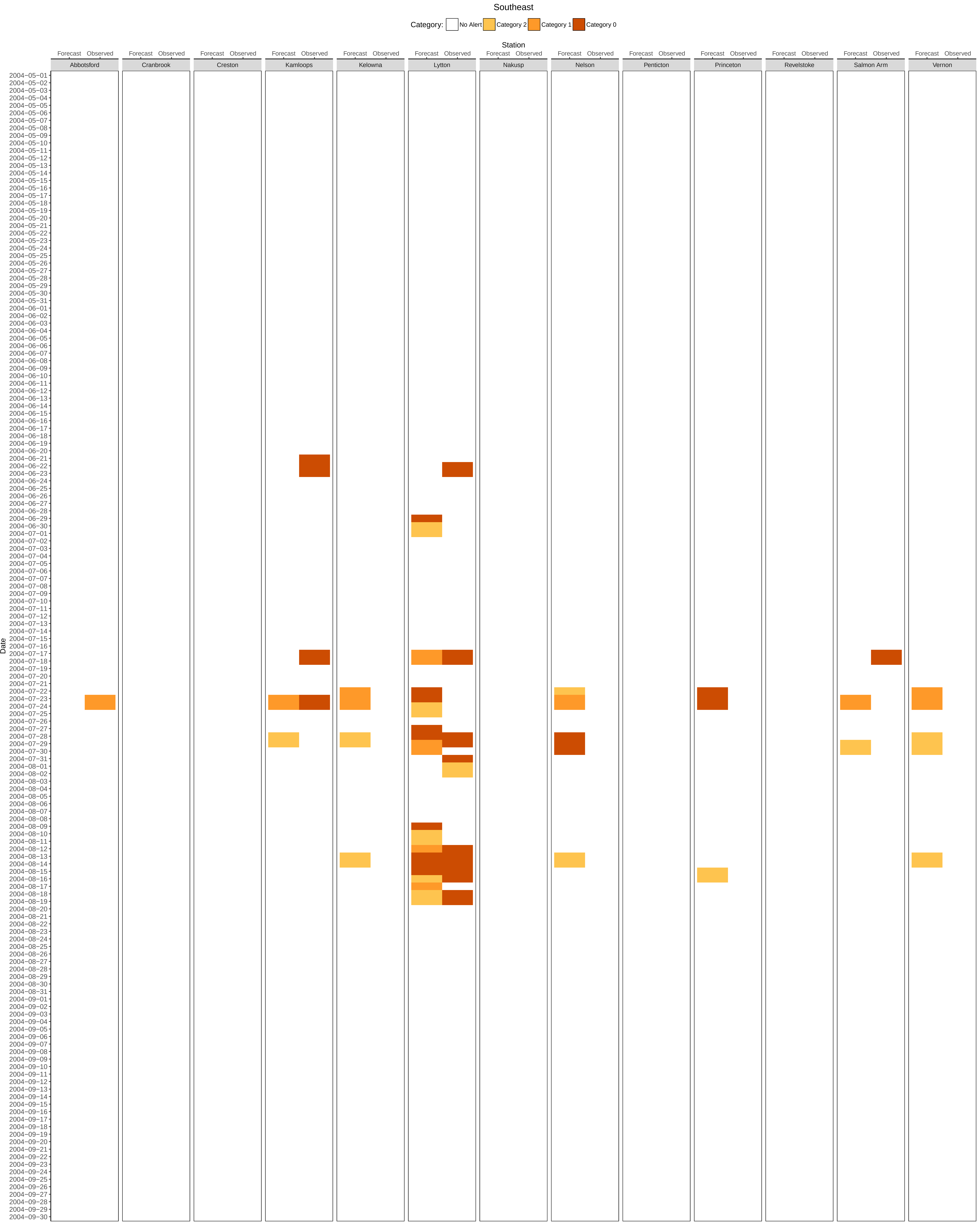

Southeast
Category: No Alert Category 2 Category 1 Category 0
Station
Forecast Observed
Abbotsford
Cranbrook
Creston
Kamloops
Kelowna
Lytton
Nakusp
Nelson
Penticton
Princeton
Revelstoke
Salmon Arm
Vernon
Date
2004−05−01
2004−09−30

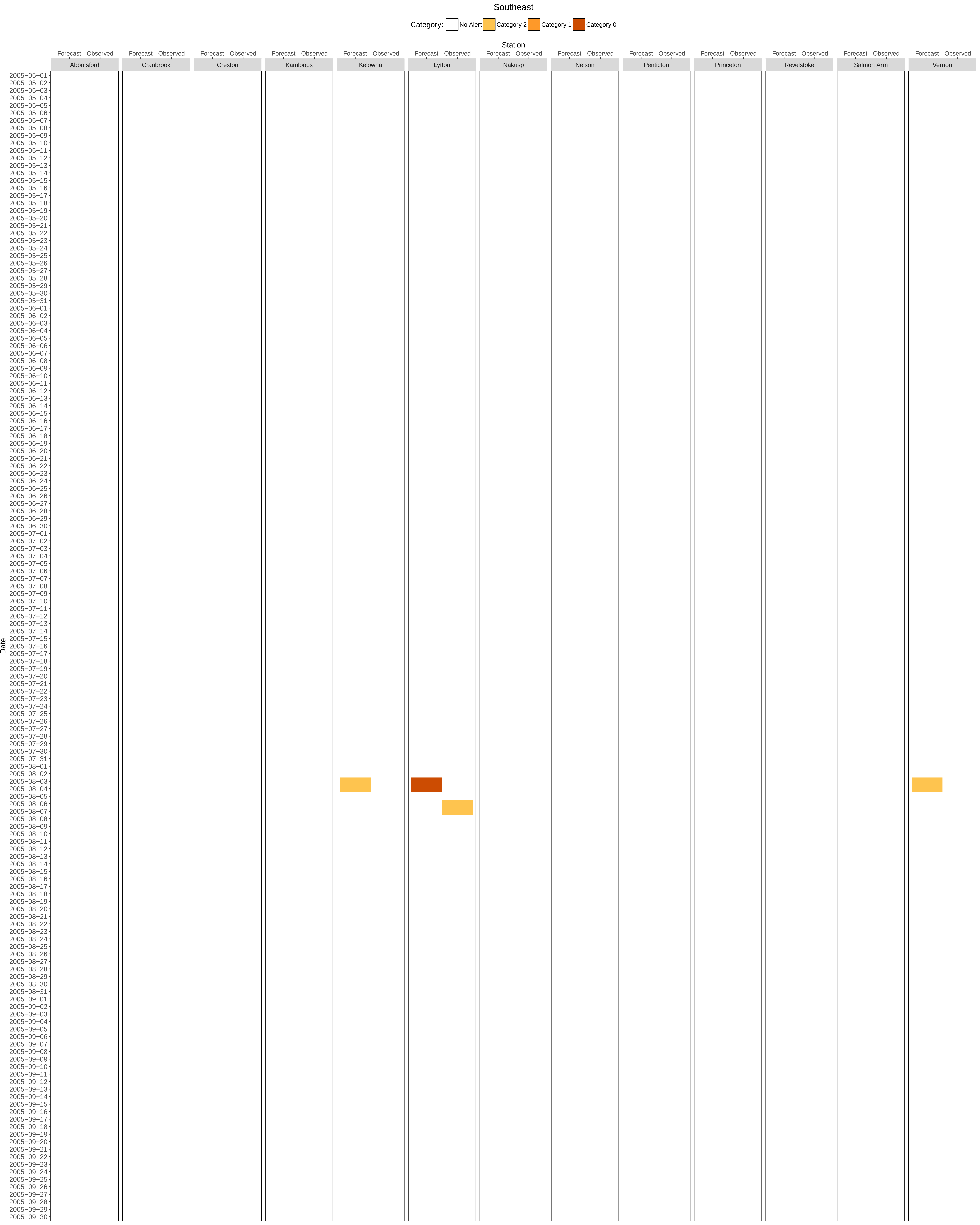
Southeast
Category:
No Alert
Category 2
Category 1
Category 0
Station
Forecast
Observed
Abbotsford
Cranbrook
Creston
Kamloops
Kelowna
Lytton
Nakusp
Nelson
Penticton
Princeton
Revelstoke
Salmon Arm
Vernon
Date
2005−05−01
2005−09−30

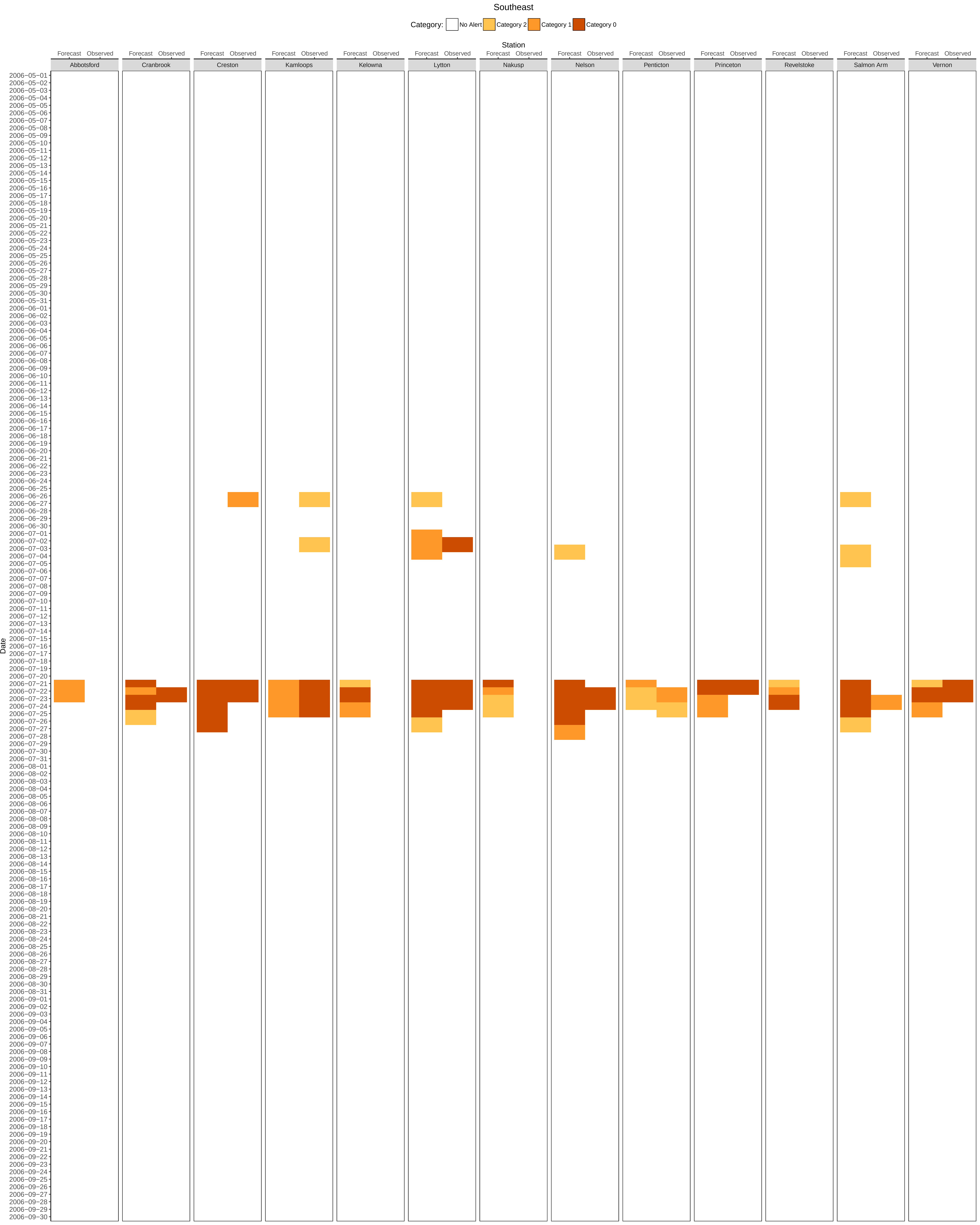

Southeast
Category:
No Alert
Category 2
Category 1
Category 0
Station
Forecast
Observed
Abbotsford
Cranbrook
Creston
Kamloops
Kelowna
Lytton
Nakusp
Nelson
Penticton
Princeton
Revelstoke
Salmon Arm
Vernon
Date
2006−05−01
2006−09−30

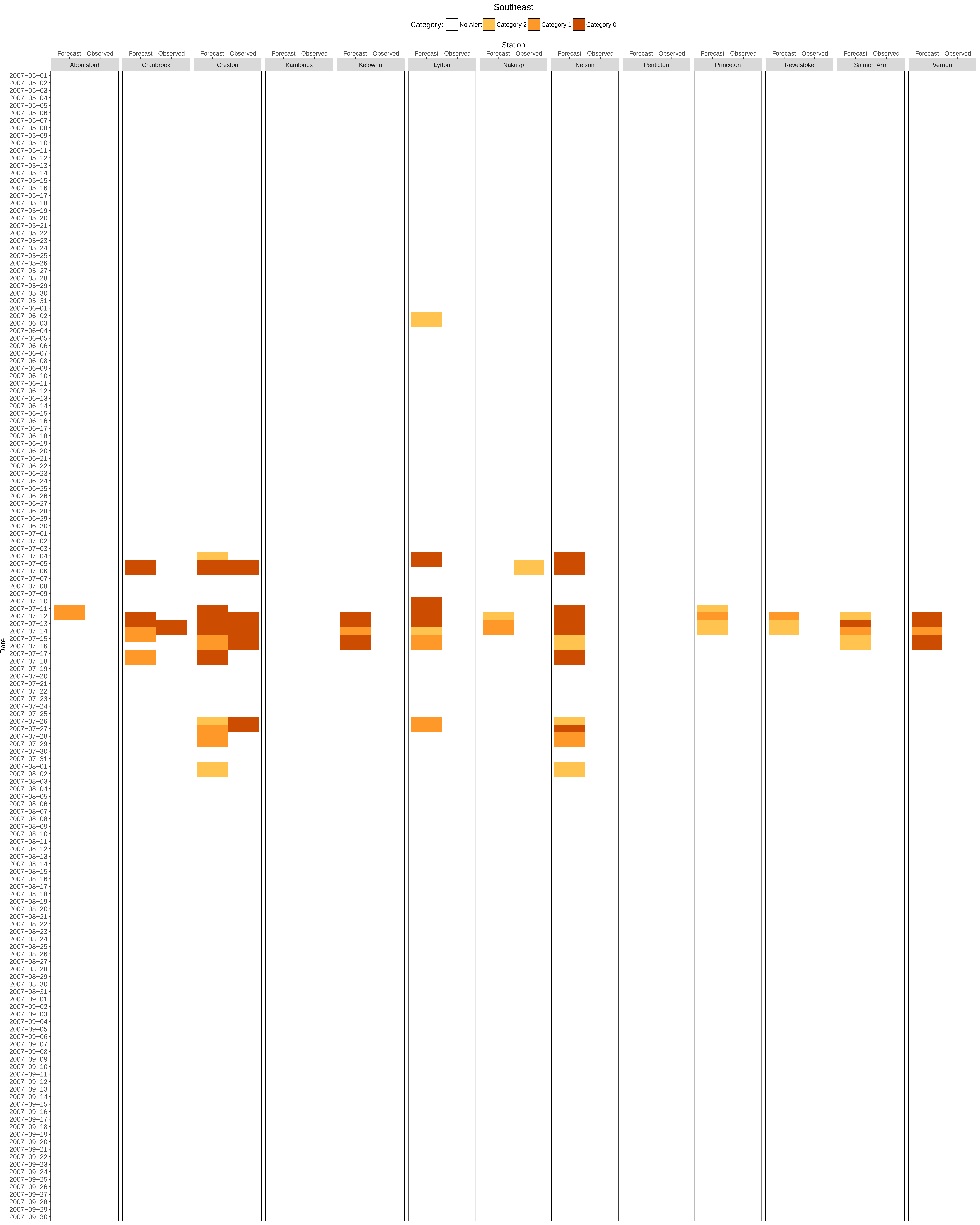
Southeast
Category: No Alert Category 2 Category 1 Category 0
Station
Forecast Observed
Abbotsford
Cranbrook
Creston
Kamloops
Kelowna
Lytton
Nakusp
Nelson
Penticton
Princeton
Revelstoke
Salmon Arm
Vernon
Date
2007−05−01
2007−09−30

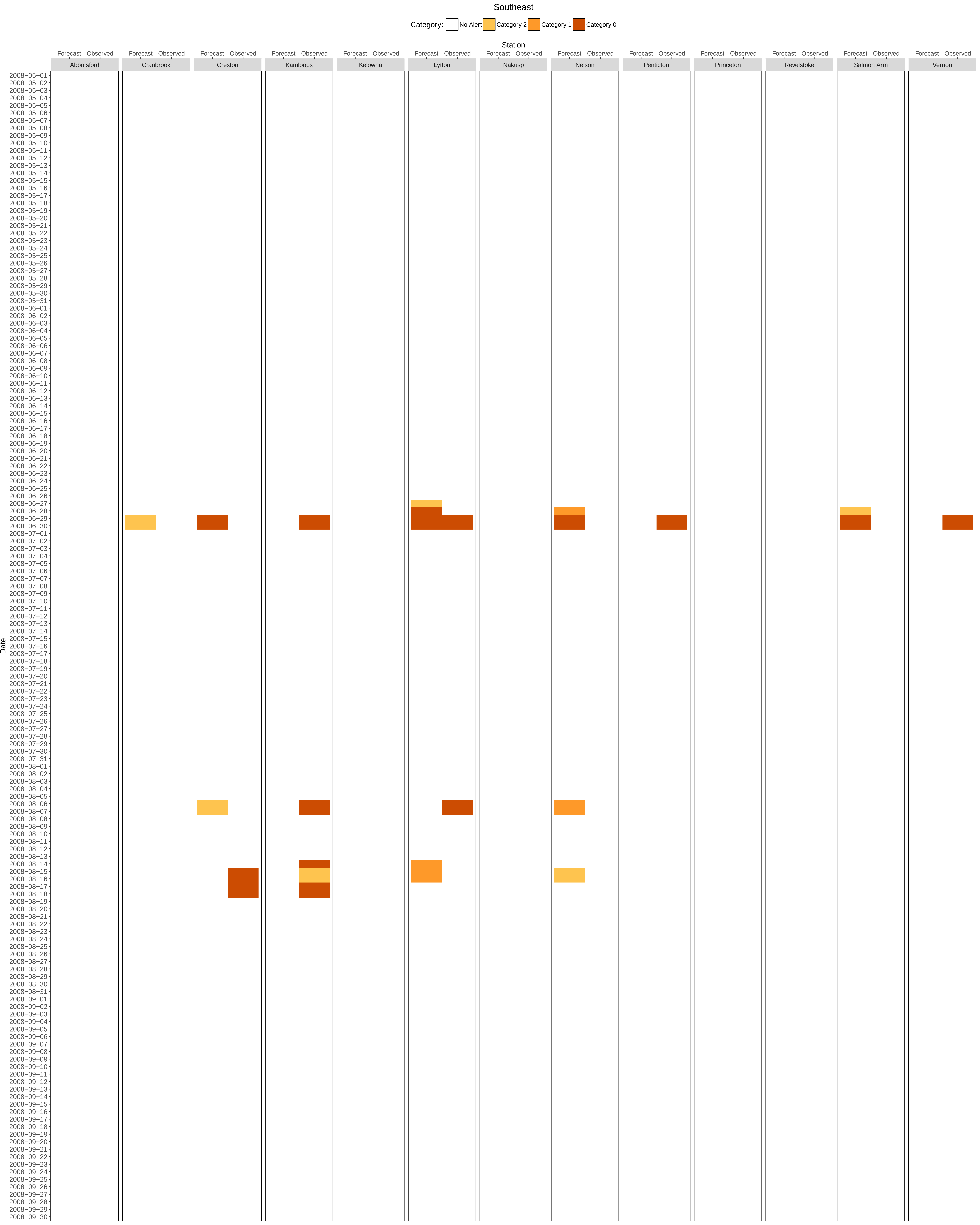

Southeast
Category: No Alert Category 2 Category 1 Category 0
Station
Forecast Observed
Abbotsford
Cranbrook
Creston
Kamloops
Kelowna
Lytton
Nakusp
Nelson
Penticton
Princeton
Revelstoke
Salmon Arm
Vernon
Date
2008−05−01
2008−09−30

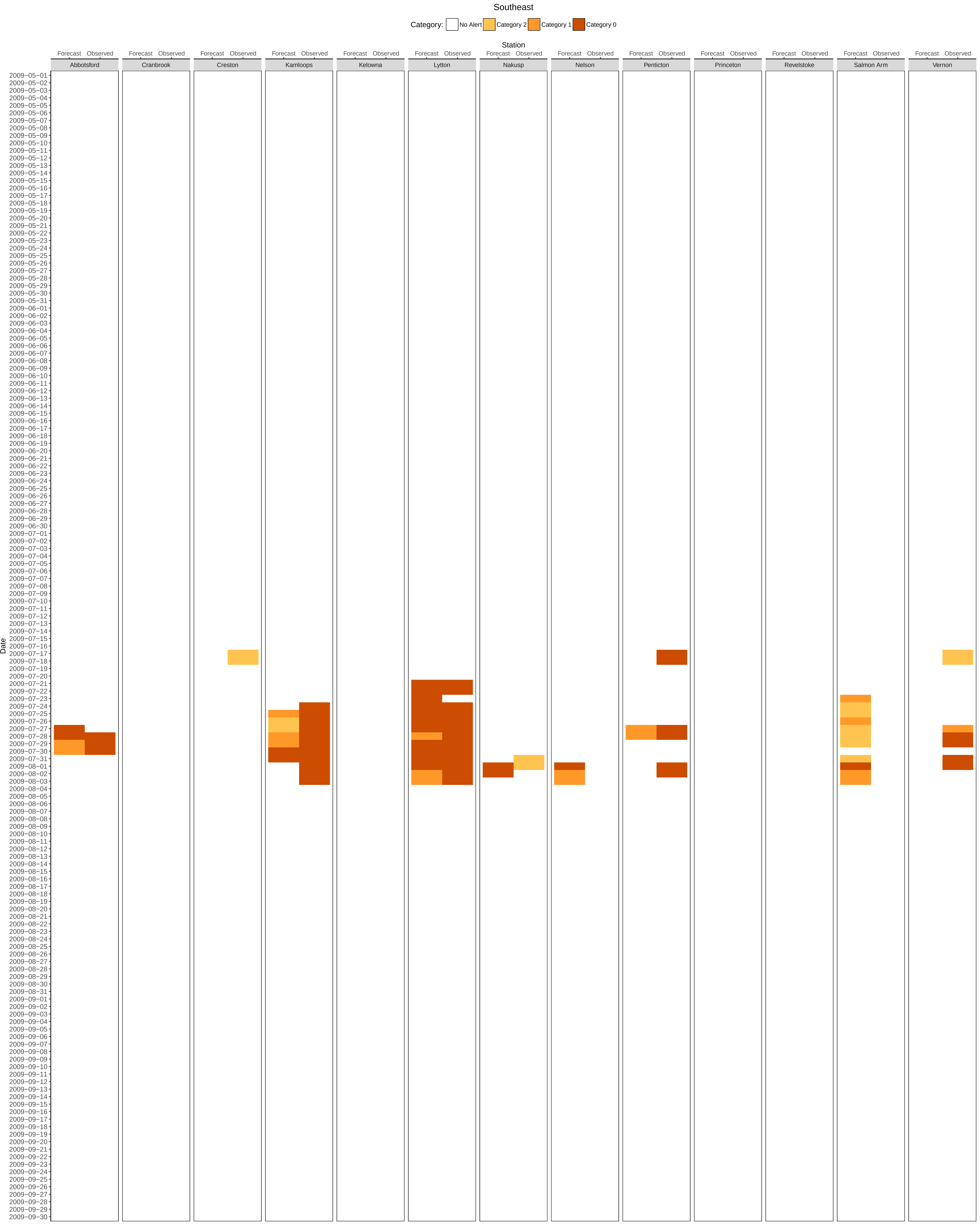
Southeast
Category:
No Alert
Category 2
Category 1
Category 0
Station
Forecast
Observed
Abbotsford
Cranbrook
Creston
Kamloops
Kelowna
Lytton
Nakusp
Nelson
Penticton
Princeton
Revelstoke
Salmon Arm
Vernon
Date
2009−05−01
2009−09−30

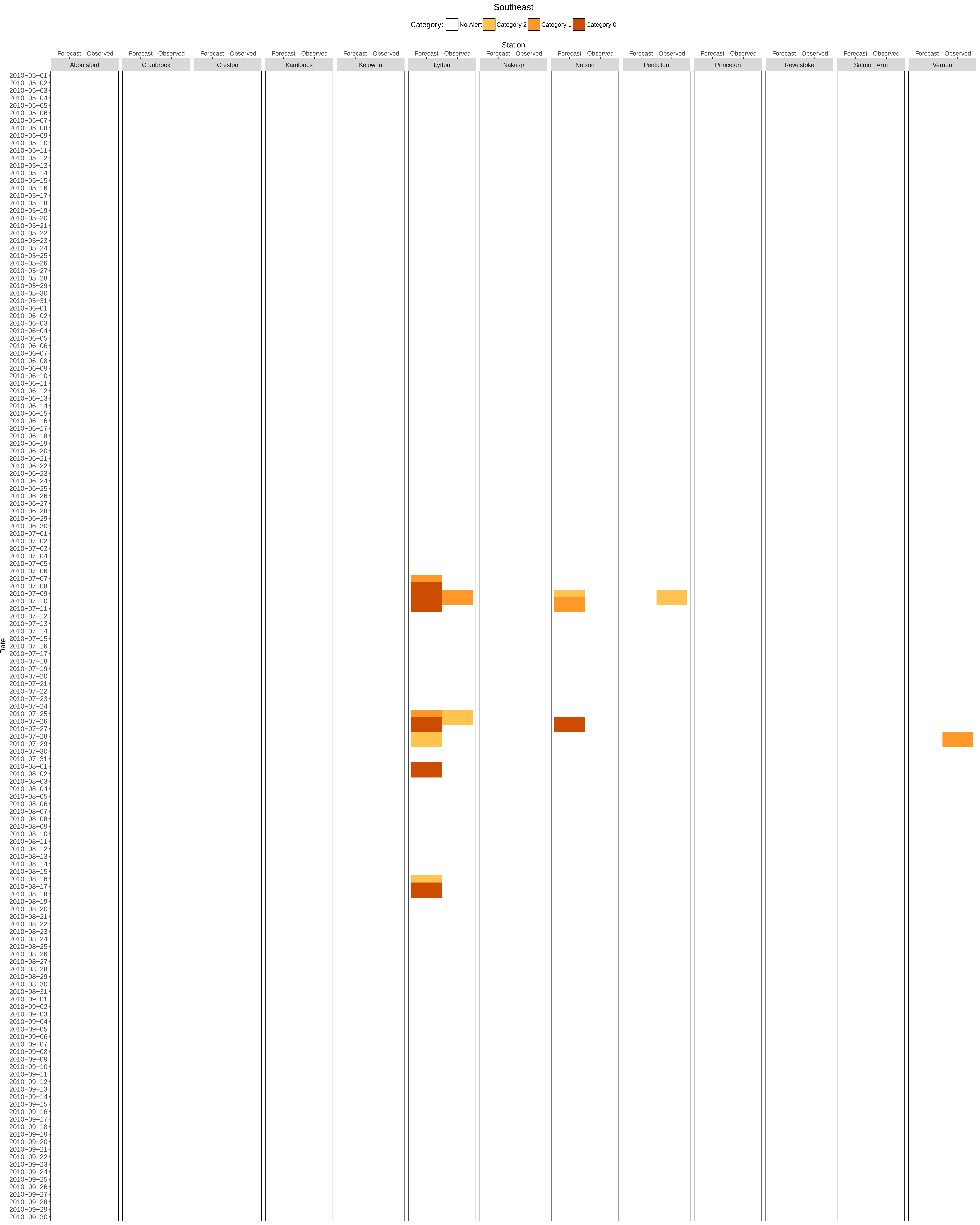
Southeast
Category:
No Alert
Category 2
Category 1
Category 0
Station
Forecast
Observed
Abbotsford
Cranbrook
Creston
Kamloops
Kelowna
Lytton
Nakusp
Nelson
Penticton
Princeton
Revelstoke
Salmon Arm
Vernon
Date
2010−05−01
2010−05−02
2010−05−03
2010−05−04
2010−05−05
2010−05−06
2010−05−07
2010−05−08
2010−05−09
2010−05−10
2010−05−11
2010−05−12
2010−05−13
2010−05−14
2010−05−15
2010−05−16
2010−05−17
2010−05−18
2010−05−19
2010−05−20
2010−05−21
2010−05−22
2010−05−23
2010−05−24
2010−05−25
2010−05−26
2010−05−27
2010−05−28
2010−05−29
2010−05−30
2010−05−31
2010−06−01
2010−06−02
2010−06−03
2010−06−04
2010−06−05
2010−06−06
2010−06−07
2010−06−08
2010−06−09
2010−06−10
2010−06−11
2010−06−12
2010−06−13
2010−06−14
2010−06−15
2010−06−16
2010−06−17
2010−06−18
2010−06−19
2010−06−20
2010−06−21
2010−06−22
2010−06−23
2010−06−24
2010−06−25
2010−06−26
2010−06−27
2010−06−28
2010−06−29
2010−06−30
2010−07−01
2010−07−02
2010−07−03
2010−07−04
2010−07−05
2010−07−06
2010−07−07
2010−07−08
2010−07−09
2010−07−10
2010−07−11
2010−07−12
2010−07−13
2010−07−14
2010−07−15
2010−07−16
2010−07−17
2010−07−18
2010−07−19
2010−07−20
2010−07−21
2010−07−22
2010−07−23
2010−07−24
2010−07−25
2010−07−26
2010−07−27
2010−07−28
2010−07−29
2010−07−30
2010−07−31
2010−08−01
2010−08−02
2010−08−03
2010−08−04
2010−08−05
2010−08−06
2010−08−07
2010−08−08
2010−08−09
2010−08−10
2010−08−11
2010−08−12
2010−08−13
2010−08−14
2010−08−15
2010−08−16
2010−08−17
2010−08−18
2010−08−19
2010−08−20
2010−08−21
2010−08−22
2010−08−23
2010−08−24
2010−08−25
2010−08−26
2010−08−27
2010−08−28
2010−08−29
2010−08−30
2010−08−31
2010−09−01
2010−09−02
2010−09−03
2010−09−04
2010−09−05
2010−09−06
2010−09−07
2010−09−08
2010−09−09
2010−09−10
2010−09−11
2010−09−12
2010−09−13
2010−09−14
2010−09−15
2010−09−16
2010−09−17
2010−09−18
2010−09−19
2010−09−20
2010−09−21
2010−09−22
2010−09−23
2010−09−24
2010−09−25
2010−09−26
2010−09−27
2010−09−28
2010−09−29
2010−09−30

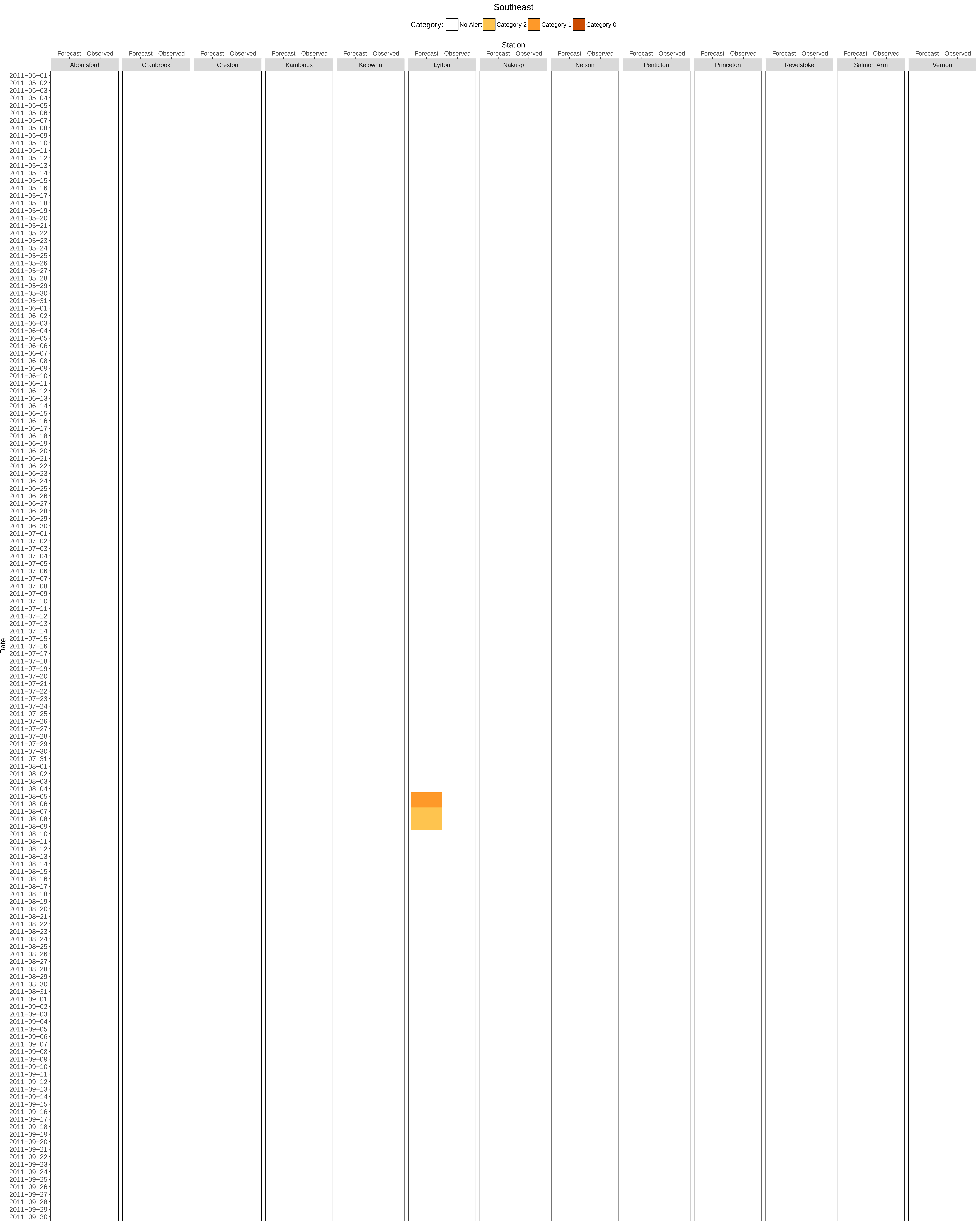

Southeast
Category:
No Alert
Category 2
Category 1
Category 0
Station
Forecast
Observed
Abbotsford
Cranbrook
Creston
Kamloops
Kelowna
Lytton
Nakusp
Nelson
Penticton
Princeton
Revelstoke
Salmon Arm
Vernon
Date
2011−05−01
2011−05−02
2011−05−03
2011−05−04
2011−05−05
2011−05−06
2011−05−07
2011−05−08
2011−05−09
2011−05−10
2011−05−11
2011−05−12
2011−05−13
2011−05−14
2011−05−15
2011−05−16
2011−05−17
2011−05−18
2011−05−19
2011−05−20
2011−05−21
2011−05−22
2011−05−23
2011−05−24
2011−05−25
2011−05−26
2011−05−27
2011−05−28
2011−05−29
2011−05−30
2011−05−31
2011−06−01
2011−06−02
2011−06−03
2011−06−04
2011−06−05
2011−06−06
2011−06−07
2011−06−08
2011−06−09
2011−06−10
2011−06−11
2011−06−12
2011−06−13
2011−06−14
2011−06−15
2011−06−16
2011−06−17
2011−06−18
2011−06−19
2011−06−20
2011−06−21
2011−06−22
2011−06−23
2011−06−24
2011−06−25
2011−06−26
2011−06−27
2011−06−28
2011−06−29
2011−06−30
2011−07−01
2011−07−02
2011−07−03
2011−07−04
2011−07−05
2011−07−06
2011−07−07
2011−07−08
2011−07−09
2011−07−10
2011−07−11
2011−07−12
2011−07−13
2011−07−14
2011−07−15
2011−07−16
2011−07−17
2011−07−18
2011−07−19
2011−07−20
2011−07−21
2011−07−22
2011−07−23
2011−07−24
2011−07−25
2011−07−26
2011−07−27
2011−07−28
2011−07−29
2011−07−30
2011−07−31
2011−08−01
2011−08−02
2011−08−03
2011−08−04
2011−08−05
2011−08−06
2011−08−07
2011−08−08
2011−08−09
2011−08−10
2011−08−11
2011−08−12
2011−08−13
2011−08−14
2011−08−15
2011−08−16
2011−08−17
2011−08−18
2011−08−19
2011−08−20
2011−08−21
2011−08−22
2011−08−23
2011−08−24
2011−08−25
2011−08−26
2011−08−27
2011−08−28
2011−08−29
2011−08−30
2011−08−31
2011−09−01
2011−09−02
2011−09−03
2011−09−04
2011−09−05
2011−09−06
2011−09−07
2011−09−08
2011−09−09
2011−09−10
2011−09−11
2011−09−12
2011−09−13
2011−09−14
2011−09−15
2011−09−16
2011−09−17
2011−09−18
2011−09−19
2011−09−20
2011−09−21
2011−09−22
2011−09−23
2011−09−24
2011−09−25
2011−09−26
2011−09−27
2011−09−28
2011−09−29
2011−09−30

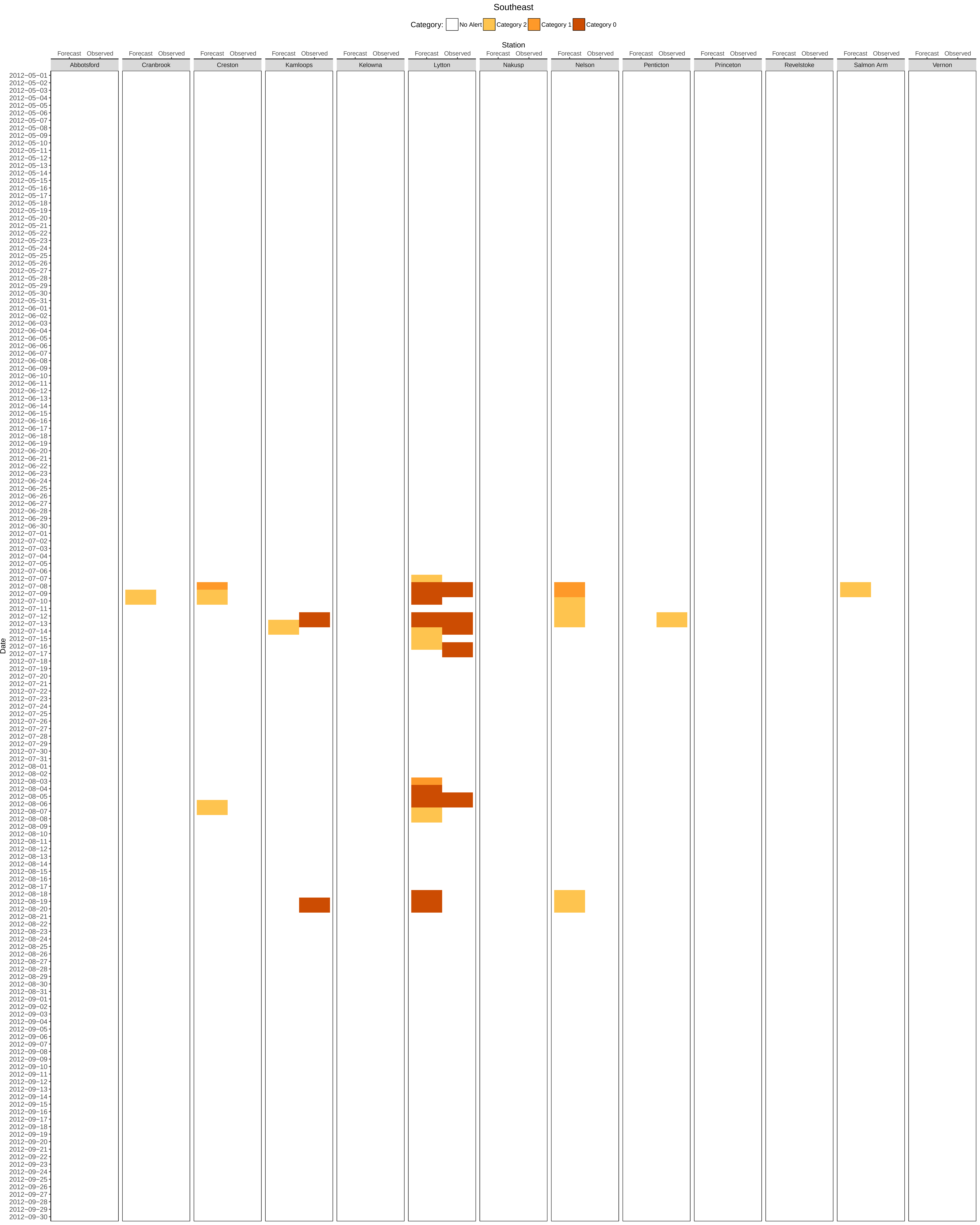
Southeast
Category: No Alert Category 2 Category 1 Category 0
Station
Forecast Observed
Abbotsford
Cranbrook
Creston
Kamloops
Kelowna
Lytton
Nakusp
Nelson
Penticton
Princeton
Revelstoke
Salmon Arm
Vernon
Date
2012−05−01
2012−09−30

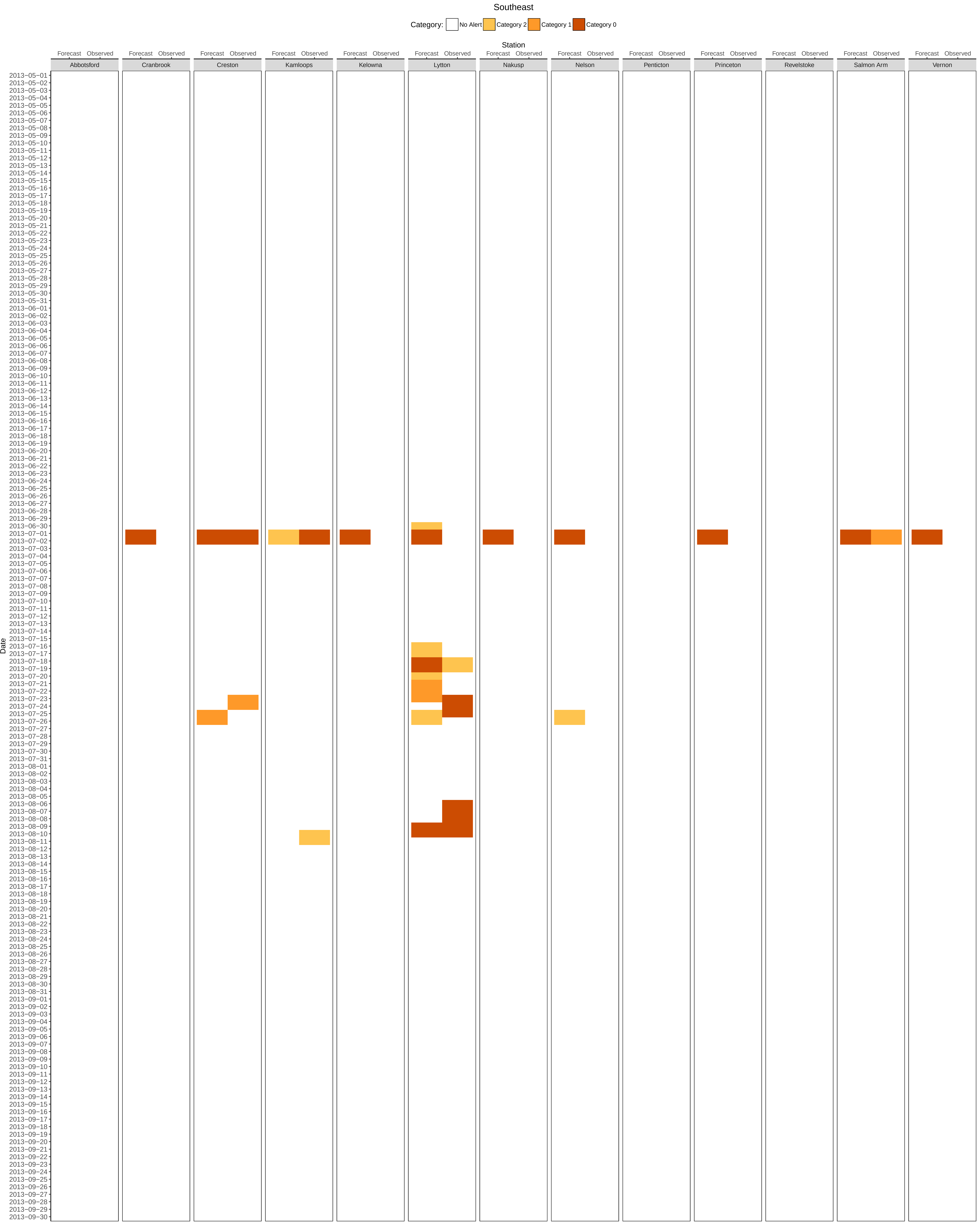
Southeast
Category: No Alert Category 2 Category 1 Category 0
Station
Forecast Observed
Abbotsford
Cranbrook
Creston
Kamloops
Kelowna
Lytton
Nakusp
Nelson
Penticton
Princeton
Revelstoke
Salmon Arm
Vernon
Date
2013−05−01
2013−09−30

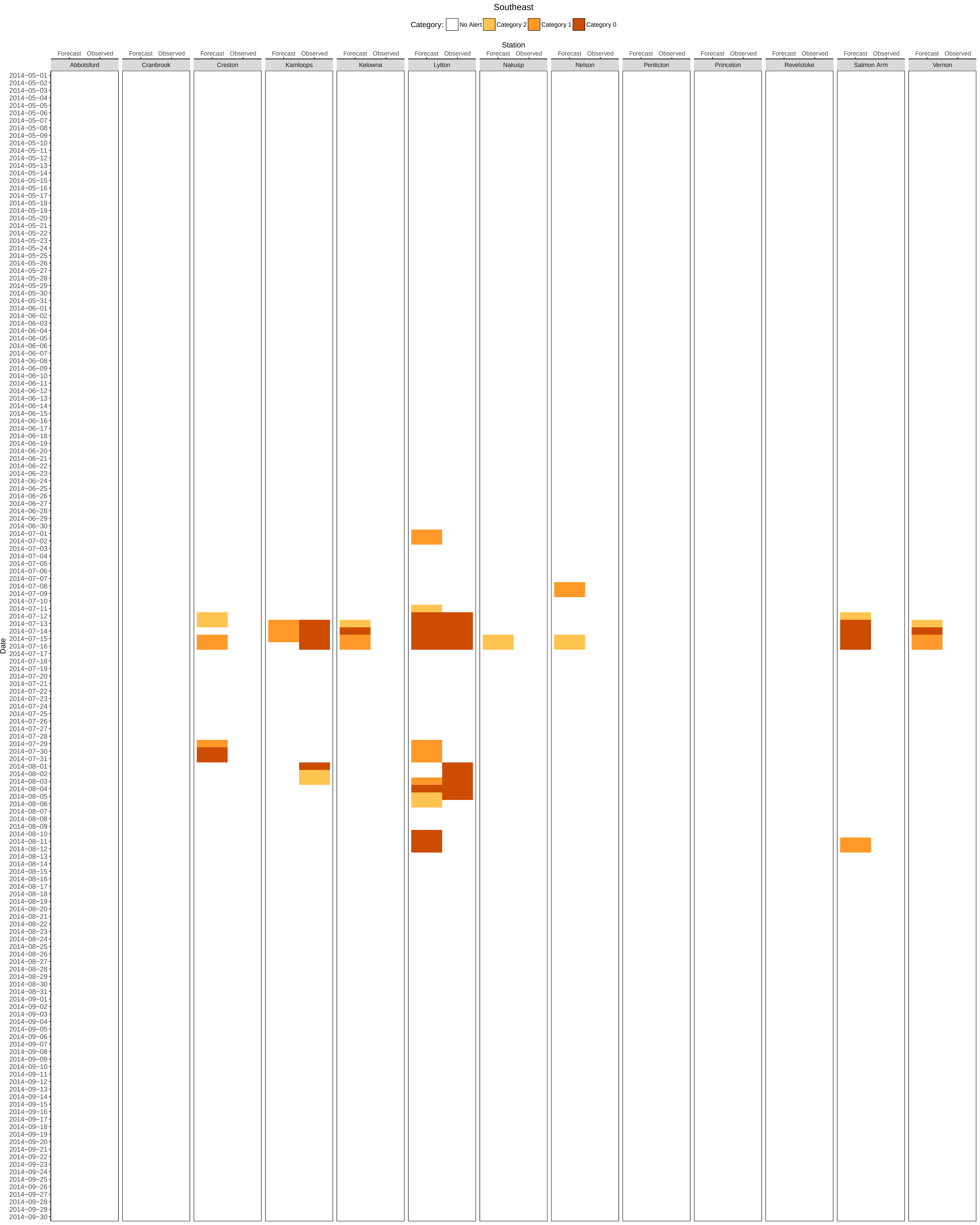

Southeast
Category: No Alert Category 2 Category 1 Category 0
Station
Forecast Observed
Abbotsford
Cranbrook
Creston
Kamloops
Kelowna
Lytton
Nakusp
Nelson
Penticton
Princeton
Revelstoke
Salmon Arm
Vernon
Date
2014−05−01
2014−09−30

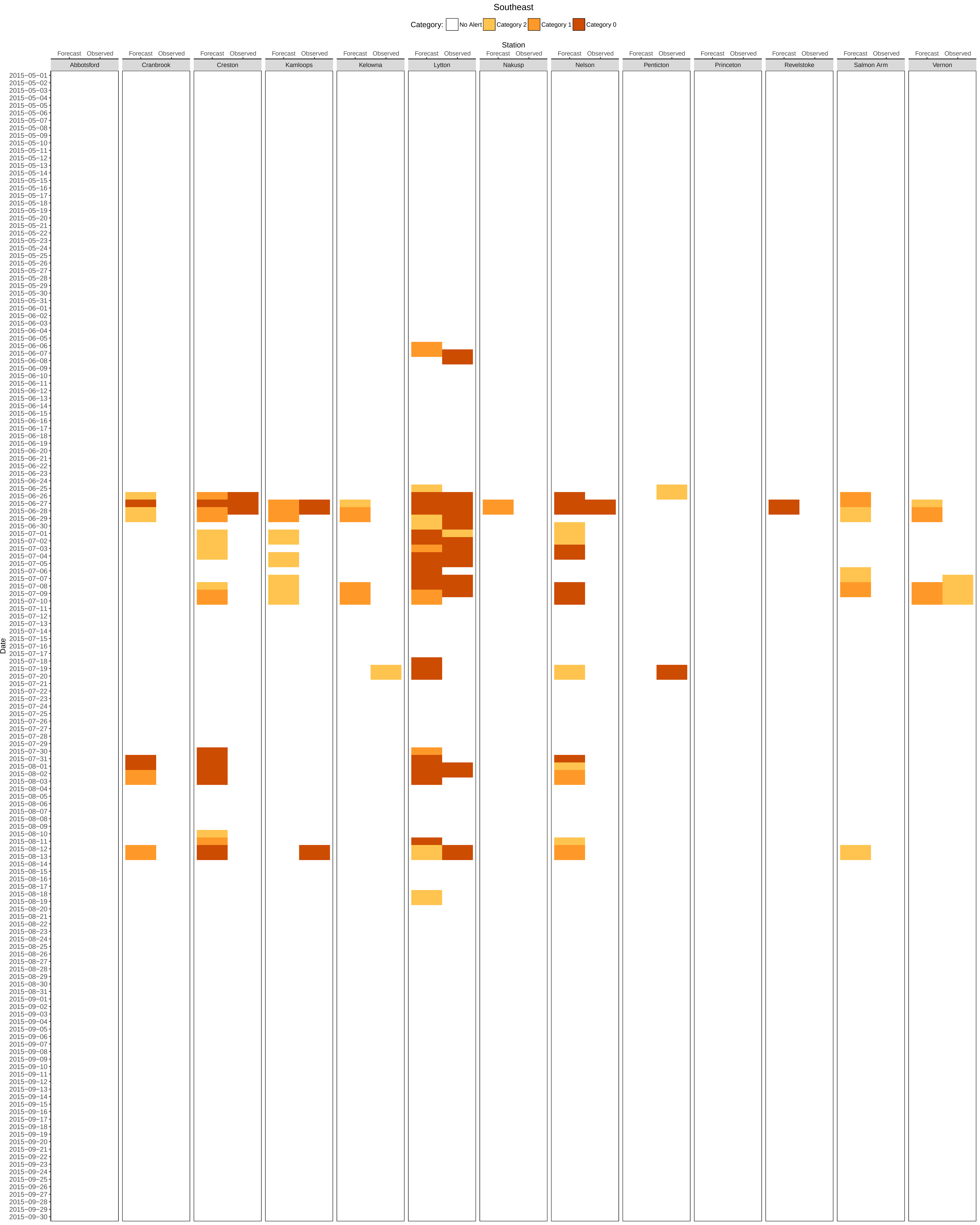
Southeast
Category:
No Alert
Category 2
Category 1
Category 0
Station
Forecast
Observed
Abbotsford
Cranbrook
Creston
Kamloops
Kelowna
Lytton
Nakusp
Nelson
Penticton
Princeton
Revelstoke
Salmon Arm
Vernon
Date
2015−05−01
2015−09−30

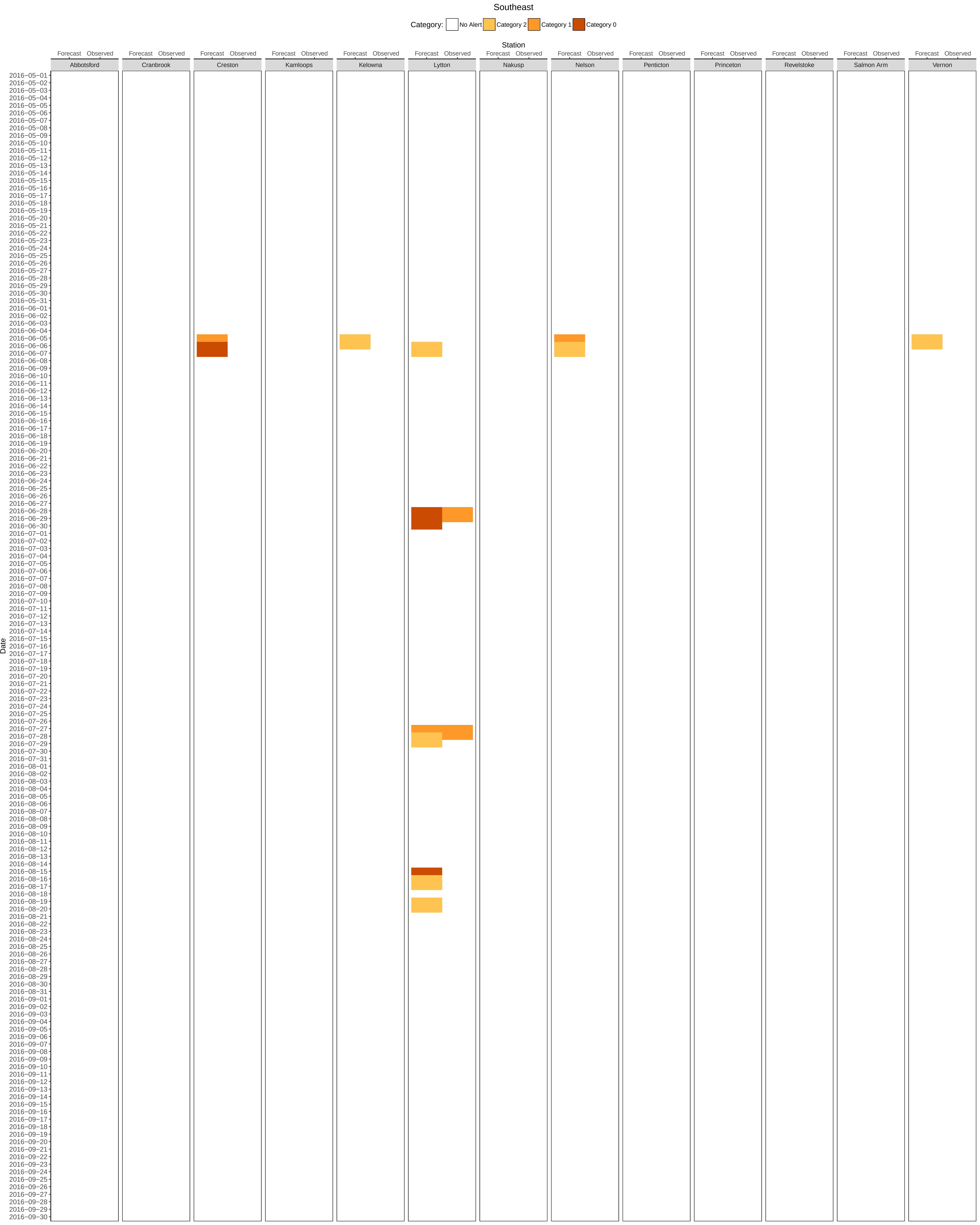
Southeast
Category:
No Alert
Category 2
Category 1
Category 0
Station
Forecast
Observed
Abbotsford
Cranbrook
Creston
Kamloops
Kelowna
Lytton
Nakusp
Nelson
Penticton
Princeton
Revelstoke
Salmon Arm
Vernon
Date
2016−05−01
2016−05−31
2016−06−30
2016−07−31
2016−08−31
2016−09−30
