# Supplementary material for: Establishing Heat Alert Thresholds for the Varied Climatic Regions of British Columbia, Canada
Source: Int J Environ Res Public Health. 2018 Sep 19;15(9):2048. doi: 10.3390/ijerph15092048 (PMC6163932; doi:10.3390/ijerph15092048)
Supplement: Supplementary file 1 [file ijerph-15-02048-s001.zip › ijerph-343571-Supplementary materials-proofreading/ijerph-343571-Supplementary materials-proofreading/S9_HeatAlertsPerYear_Northeast.pdf]

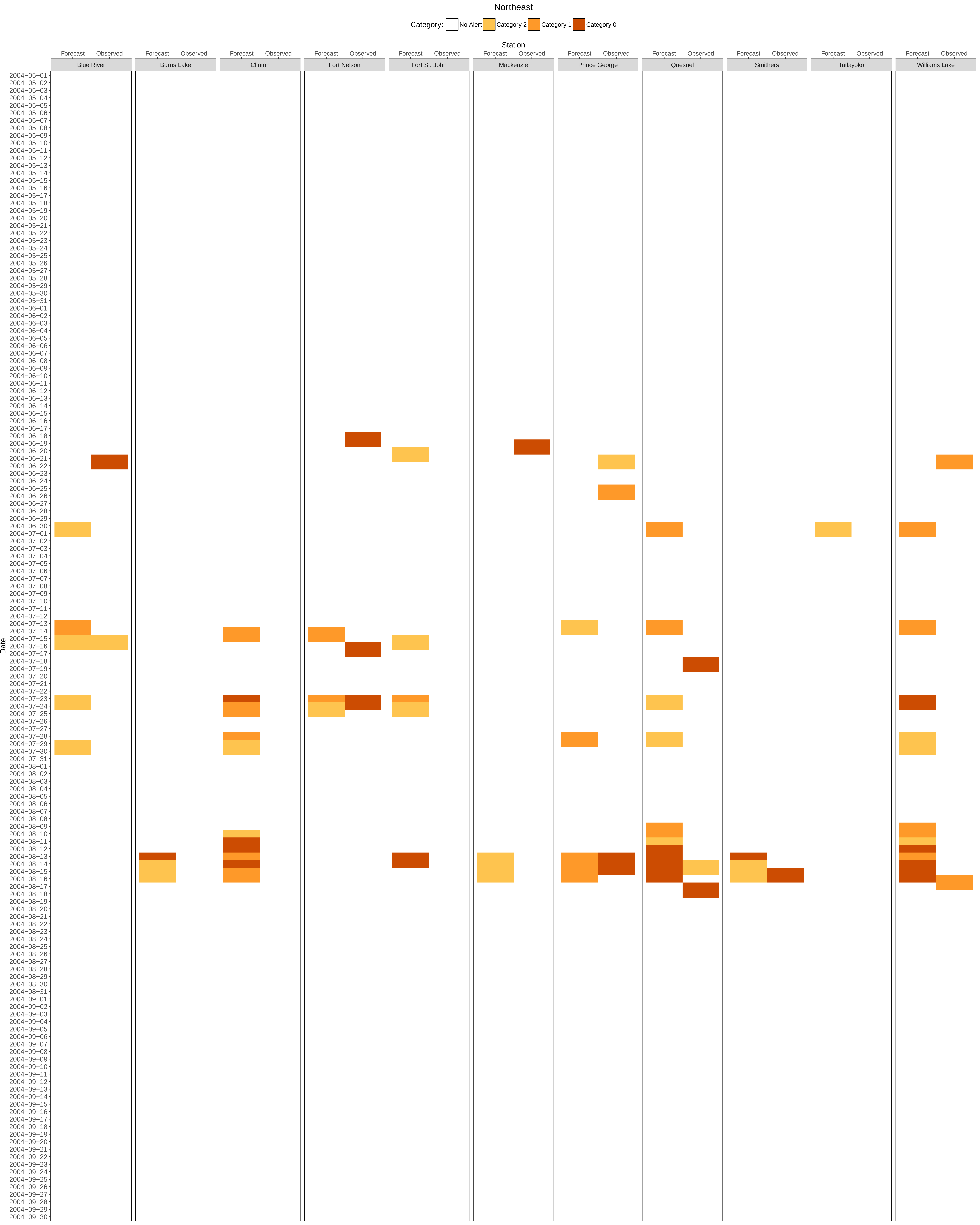

Northeast
Category: No Alert Category 2 Category 1 Category 0
Station
Forecast Observed
Blue River
Burns Lake
Clinton
Fort Nelson
Fort St. John
Mackenzie
Prince George
Quesnel
Smithers
Tatlayoko
Williams Lake
Date
2004−05−01
2004−09−30

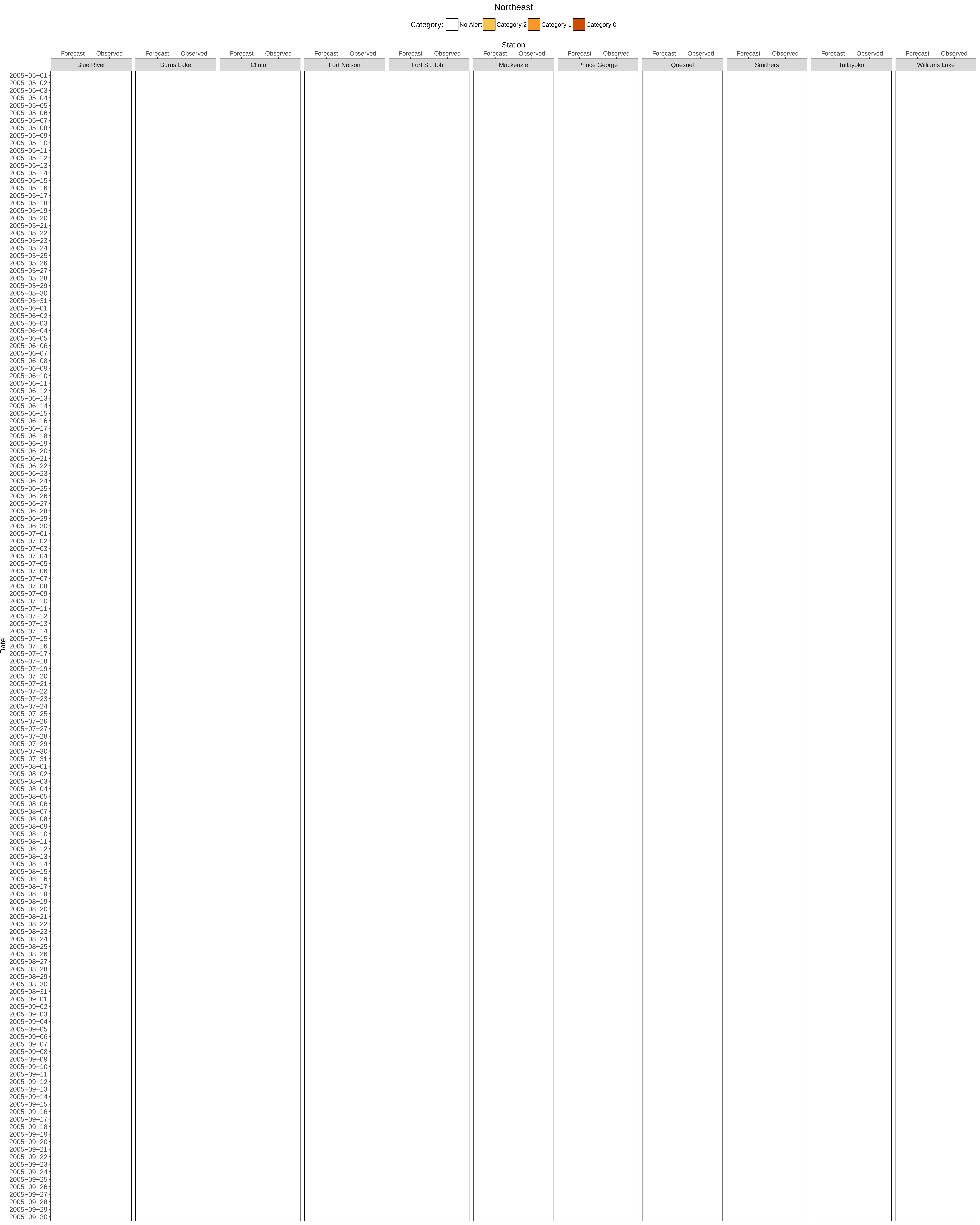
Northeast
Category:
No Alert
Category 2
Category 1
Category 0
Station
Forecast
Observed
Blue River
Burns Lake
Clinton
Fort Nelson
Fort St. John
Mackenzie
Prince George
Quesnel
Smithers
Tatlayoko
Williams Lake
Date
2005−05−01
2005−05−02
2005−05−03
2005−05−04
2005−05−05
2005−05−06
2005−05−07
2005−05−08
2005−05−09
2005−05−10
2005−05−11
2005−05−12
2005−05−13
2005−05−14
2005−05−15
2005−05−16
2005−05−17
2005−05−18
2005−05−19
2005−05−20
2005−05−21
2005−05−22
2005−05−23
2005−05−24
2005−05−25
2005−05−26
2005−05−27
2005−05−28
2005−05−29
2005−05−30
2005−05−31
2005−06−01
2005−06−02
2005−06−03
2005−06−04
2005−06−05
2005−06−06
2005−06−07
2005−06−08
2005−06−09
2005−06−10
2005−06−11
2005−06−12
2005−06−13
2005−06−14
2005−06−15
2005−06−16
2005−06−17
2005−06−18
2005−06−19
2005−06−20
2005−06−21
2005−06−22
2005−06−23
2005−06−24
2005−06−25
2005−06−26
2005−06−27
2005−06−28
2005−06−29
2005−06−30
2005−07−01
2005−07−02
2005−07−03
2005−07−04
2005−07−05
2005−07−06
2005−07−07
2005−07−08
2005−07−09
2005−07−10
2005−07−11
2005−07−12
2005−07−13
2005−07−14
2005−07−15
2005−07−16
2005−07−17
2005−07−18
2005−07−19
2005−07−20
2005−07−21
2005−07−22
2005−07−23
2005−07−24
2005−07−25
2005−07−26
2005−07−27
2005−07−28
2005−07−29
2005−07−30
2005−07−31
2005−08−01
2005−08−02
2005−08−03
2005−08−04
2005−08−05
2005−08−06
2005−08−07
2005−08−08
2005−08−09
2005−08−10
2005−08−11
2005−08−12
2005−08−13
2005−08−14
2005−08−15
2005−08−16
2005−08−17
2005−08−18
2005−08−19
2005−08−20
2005−08−21
2005−08−22
2005−08−23
2005−08−24
2005−08−25
2005−08−26
2005−08−27
2005−08−28
2005−08−29
2005−08−30
2005−08−31
2005−09−01
2005−09−02
2005−09−03
2005−09−04
2005−09−05
2005−09−06
2005−09−07
2005−09−08
2005−09−09
2005−09−10
2005−09−11
2005−09−12
2005−09−13
2005−09−14
2005−09−15
2005−09−16
2005−09−17
2005−09−18
2005−09−19
2005−09−20
2005−09−21
2005−09−22
2005−09−23
2005−09−24
2005−09−25
2005−09−26
2005−09−27
2005−09−28
2005−09−29
2005−09−30

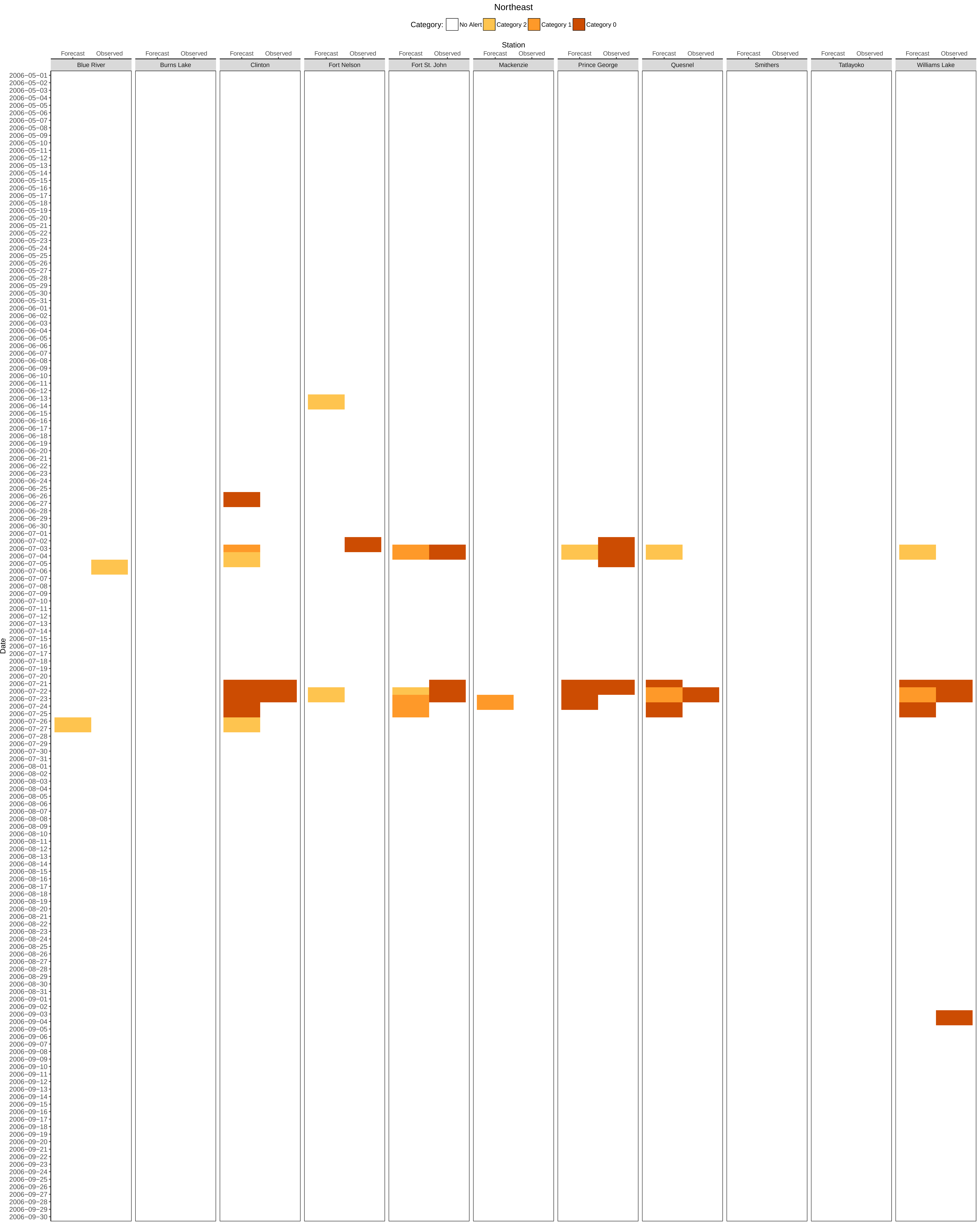

Northeast
Category:
No Alert
Category 2
Category 1
Category 0
Station
Forecast
Observed
Blue River
Burns Lake
Clinton
Fort Nelson
Fort St. John
Mackenzie
Prince George
Quesnel
Smithers
Tatlayoko
Williams Lake
Date
2006-05-01
2006-05-02
2006-05-03
2006-05-04
2006-05-05
2006-05-06
2006-05-07
2006-05-08
2006-05-09
2006-05-10
2006-05-11
2006-05-12
2006-05-13
2006-05-14
2006-05-15
2006-05-16
2006-05-17
2006-05-18
2006-05-19
2006-05-20
2006-05-21
2006-05-22
2006-05-23
2006-05-24
2006-05-25
2006-05-26
2006-05-27
2006-05-28
2006-05-29
2006-05-30
2006-05-31
2006-06-01
2006-06-02
2006-06-03
2006-06-04
2006-06-05
2006-06-06
2006-06-07
2006-06-08
2006-06-09
2006-06-10
2006-06-11
2006-06-12
2006-06-13
2006-06-14
2006-06-15
2006-06-16
2006-06-17
2006-06-18
2006-06-19
2006-06-20
2006-06-21
2006-06-22
2006-06-23
2006-06-24
2006-06-25
2006-06-26
2006-06-27
2006-06-28
2006-06-29
2006-06-30
2006-07-01
2006-07-02
2006-07-03
2006-07-04
2006-07-05
2006-07-06
2006-07-07
2006-07-08
2006-07-09
2006-07-10
2006-07-11
2006-07-12
2006-07-13
2006-07-14
2006-07-15
2006-07-16
2006-07-17
2006-07-18
2006-07-19
2006-07-20
2006-07-21
2006-07-22
2006-07-23
2006-07-24
2006-07-25
2006-07-26
2006-07-27
2006-07-28
2006-07-29
2006-07-30
2006-07-31
2006-08-01
2006-08-02
2006-08-03
2006-08-04
2006-08-05
2006-08-06
2006-08-07
2006-08-08
2006-08-09
2006-08-10
2006-08-11
2006-08-12
2006-08-13
2006-08-14
2006-08-15
2006-08-16
2006-08-17
2006-08-18
2006-08-19
2006-08-20
2006-08-21
2006-08-22
2006-08-23
2006-08-24
2006-08-25
2006-08-26
2006-08-27
2006-08-28
2006-08-29
2006-08-30
2006-08-31
2006-09-01
2006-09-02
2006-09-03
2006-09-04
2006-09-05
2006-09-06
2006-09-07
2006-09-08
2006-09-09
2006-09-10
2006-09-11
2006-09-12
2006-09-13
2006-09-14
2006-09-15
2006-09-16
2006-09-17
2006-09-18
2006-09-19
2006-09-20
2006-09-21
2006-09-22
2006-09-23
2006-09-24
2006-09-25
2006-09-26
2006-09-27
2006-09-28
2006-09-29
2006-09-30

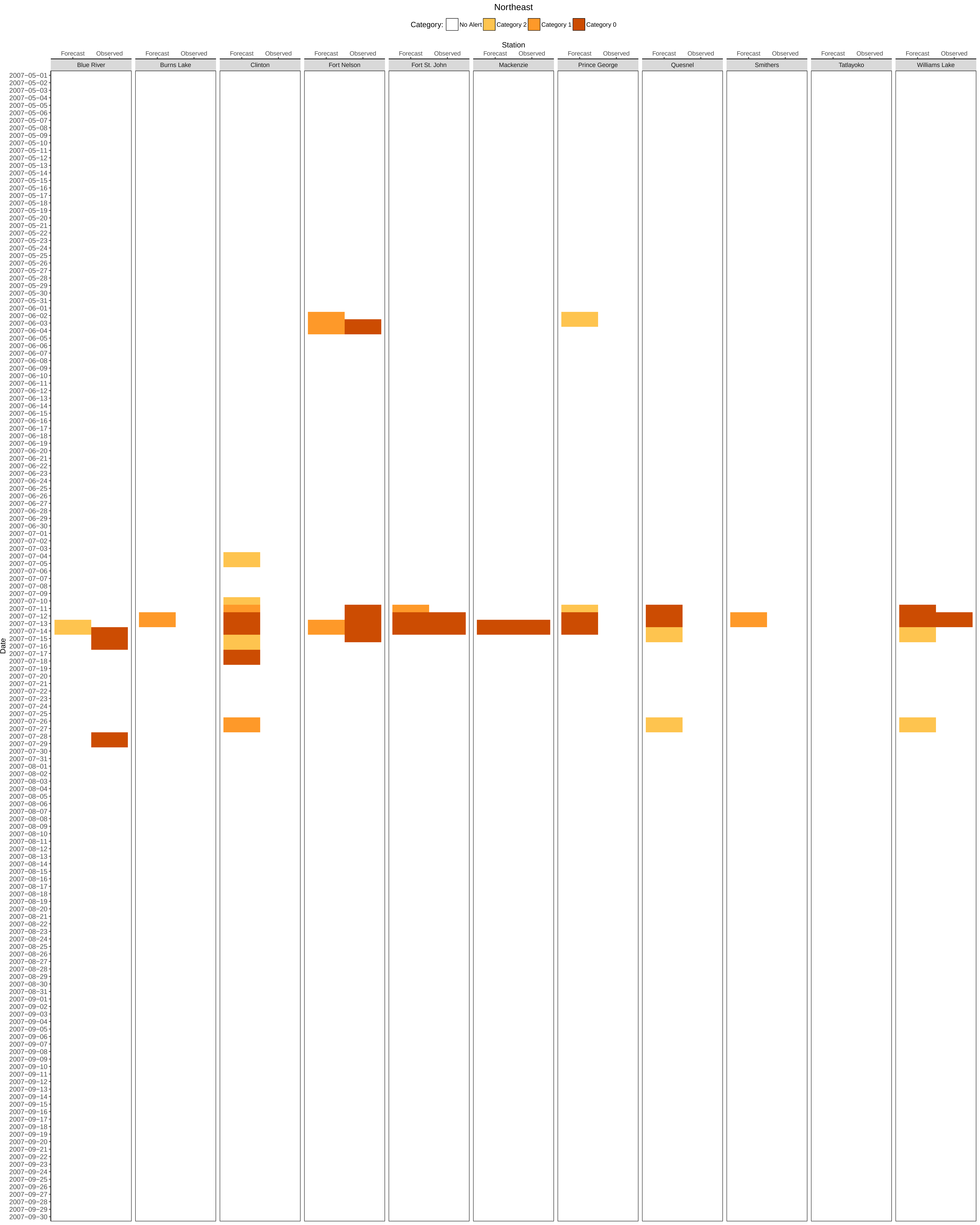
Northeast
Category:
No Alert
Category 2
Category 1
Category 0
Station
Forecast
Observed
Blue River
Burns Lake
Clinton
Fort Nelson
Fort St. John
Mackenzie
Prince George
Quesnel
Smithers
Tatlayoko
Williams Lake
Date
2007−05−01
2007−05−02
2007−05−03
2007−05−04
2007−05−05
2007−05−06
2007−05−07
2007−05−08
2007−05−09
2007−05−10
2007−05−11
2007−05−12
2007−05−13
2007−05−14
2007−05−15
2007−05−16
2007−05−17
2007−05−18
2007−05−19
2007−05−20
2007−05−21
2007−05−22
2007−05−23
2007−05−24
2007−05−25
2007−05−26
2007−05−27
2007−05−28
2007−05−29
2007−05−30
2007−05−31
2007−06−01
2007−06−02
2007−06−03
2007−06−04
2007−06−05
2007−06−06
2007−06−07
2007−06−08
2007−06−09
2007−06−10
2007−06−11
2007−06−12
2007−06−13
2007−06−14
2007−06−15
2007−06−16
2007−06−17
2007−06−18
2007−06−19
2007−06−20
2007−06−21
2007−06−22
2007−06−23
2007−06−24
2007−06−25
2007−06−26
2007−06−27
2007−06−28
2007−06−29
2007−06−30
2007−07−01
2007−07−02
2007−07−03
2007−07−04
2007−07−05
2007−07−06
2007−07−07
2007−07−08
2007−07−09
2007−07−10
2007−07−11
2007−07−12
2007−07−13
2007−07−14
2007−07−15
2007−07−16
2007−07−17
2007−07−18
2007−07−19
2007−07−20
2007−07−21
2007−07−22
2007−07−23
2007−07−24
2007−07−25
2007−07−26
2007−07−27
2007−07−28
2007−07−29
2007−07−30
2007−07−31
2007−08−01
2007−08−02
2007−08−03
2007−08−04
2007−08−05
2007−08−06
2007−08−07
2007−08−08
2007−08−09
2007−08−10
2007−08−11
2007−08−12
2007−08−13
2007−08−14
2007−08−15
2007−08−16
2007−08−17
2007−08−18
2007−08−19
2007−08−20
2007−08−21
2007−08−22
2007−08−23
2007−08−24
2007−08−25
2007−08−26
2007−08−27
2007−08−28
2007−08−29
2007−08−30
2007−08−31
2007−09−01
2007−09−02
2007−09−03
2007−09−04
2007−09−05
2007−09−06
2007−09−07
2007−09−08
2007−09−09
2007−09−10
2007−09−11
2007−09−12
2007−09−13
2007−09−14
2007−09−15
2007−09−16
2007−09−17
2007−09−18
2007−09−19
2007−09−20
2007−09−21
2007−09−22
2007−09−23
2007−09−24
2007−09−25
2007−09−26
2007−09−27
2007−09−28
2007−09−29
2007−09−30

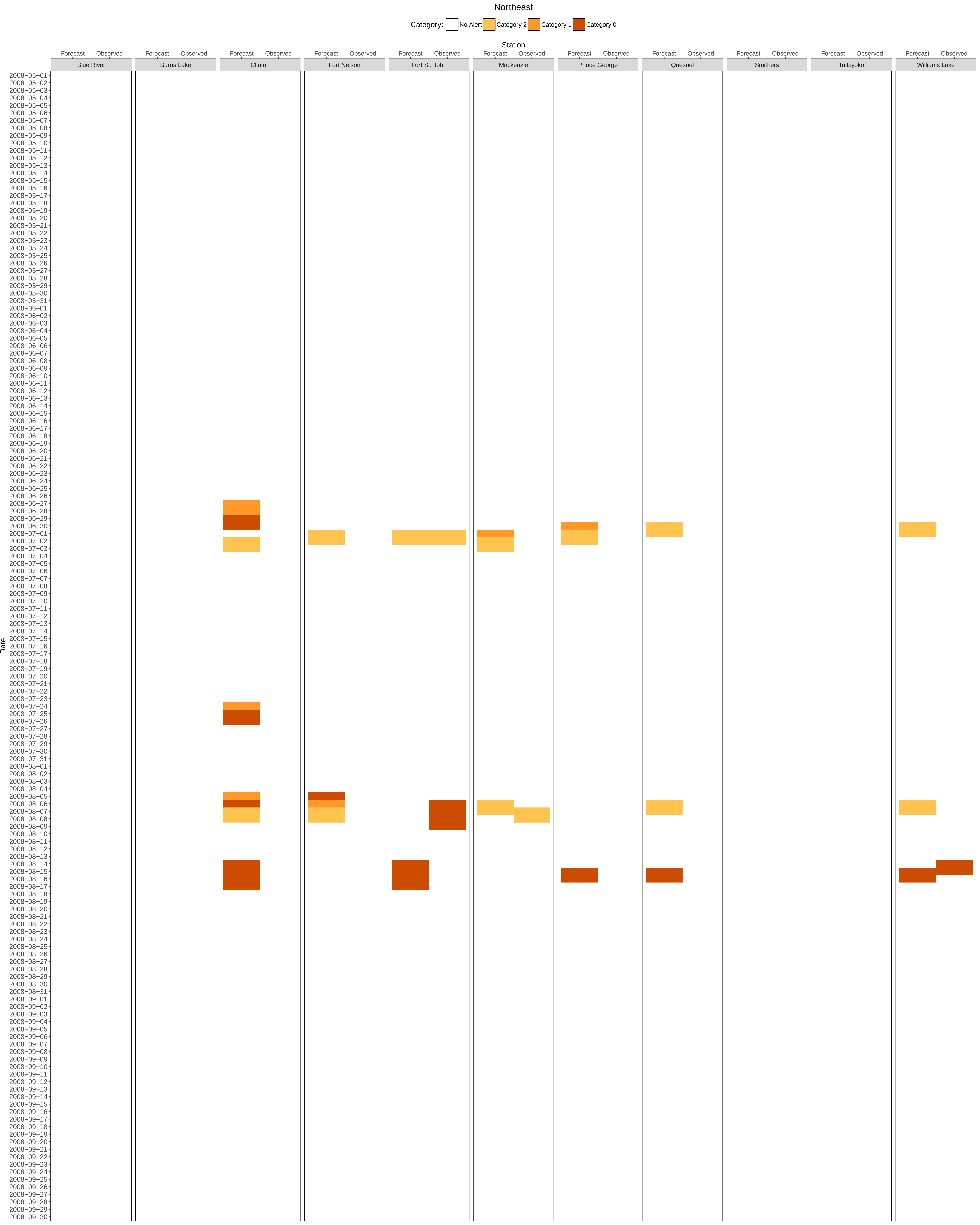
Northeast
Category: No Alert Category 2 Category 1 Category 0
Station
Blue River
Burns Lake
Clinton
Fort Nelson
Fort St. John
Mackenzie
Prince George
Quesnel
Smithers
Tatlayoko
Williams Lake
Forecast
Observed
Date
2008−05−01
2008−09−30

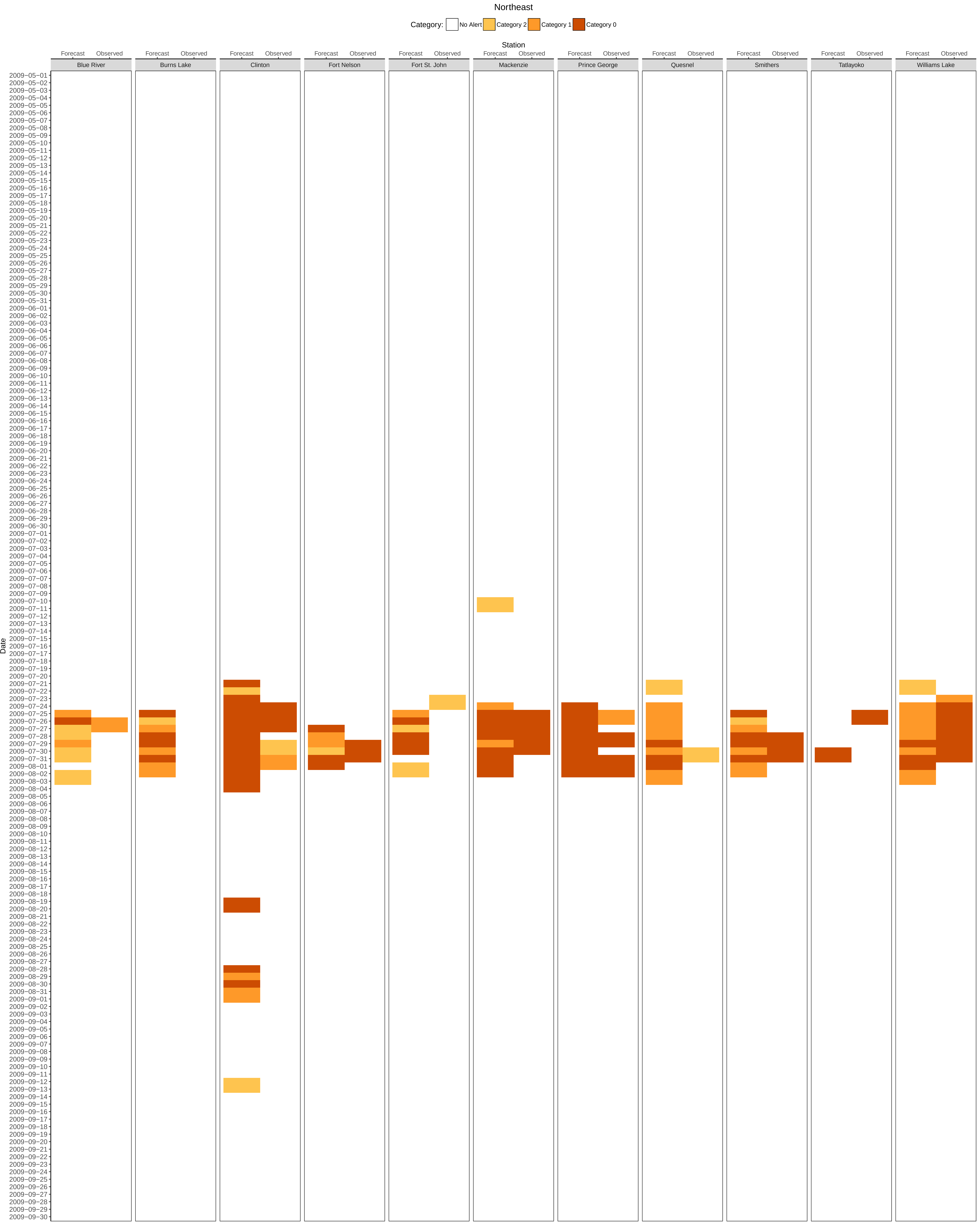

Northeast
Category: No Alert Category 2 Category 1 Category 0
Station
Forecast Observed
Blue River
Burns Lake
Clinton
Fort Nelson
Fort St. John
Mackenzie
Prince George
Quesnel
Smithers
Tatlayoko
Williams Lake
Date
2009-05-01
2009-05-02
2009-05-03
2009-05-04
2009-05-05
2009-05-06
2009-05-07
2009-05-08
2009-05-09
2009-05-10
2009-05-11
2009-05-12
2009-05-13
2009-05-14
2009-05-15
2009-05-16
2009-05-17
2009-05-18
2009-05-19
2009-05-20
2009-05-21
2009-05-22
2009-05-23
2009-05-24
2009-05-25
2009-05-26
2009-05-27
2009-05-28
2009-05-29
2009-05-30
2009-05-31
2009-06-01
2009-06-02
2009-06-03
2009-06-04
2009-06-05
2009-06-06
2009-06-07
2009-06-08
2009-06-09
2009-06-10
2009-06-11
2009-06-12
2009-06-13
2009-06-14
2009-06-15
2009-06-16
2009-06-17
2009-06-18
2009-06-19
2009-06-20
2009-06-21
2009-06-22
2009-06-23
2009-06-24
2009-06-25
2009-06-26
2009-06-27
2009-06-28
2009-06-29
2009-06-30
2009-07-01
2009-07-02
2009-07-03
2009-07-04
2009-07-05
2009-07-06
2009-07-07
2009-07-08
2009-07-09
2009-07-10
2009-07-11
2009-07-12
2009-07-13
2009-07-14
2009-07-15
2009-07-16
2009-07-17
2009-07-18
2009-07-19
2009-07-20
2009-07-21
2009-07-22
2009-07-23
2009-07-24
2009-07-25
2009-07-26
2009-07-27
2009-07-28
2009-07-29
2009-07-30
2009-07-31
2009-08-01
2009-08-02
2009-08-03
2009-08-04
2009-08-05
2009-08-06
2009-08-07
2009-08-08
2009-08-09
2009-08-10
2009-08-11
2009-08-12
2009-08-13
2009-08-14
2009-08-15
2009-08-16
2009-08-17
2009-08-18
2009-08-19
2009-08-20
2009-08-21
2009-08-22
2009-08-23
2009-08-24
2009-08-25
2009-08-26
2009-08-27
2009-08-28
2009-08-29
2009-08-30
2009-08-31
2009-09-01
2009-09-02
2009-09-03
2009-09-04
2009-09-05
2009-09-06
2009-09-07
2009-09-08
2009-09-09
2009-09-10
2009-09-11
2009-09-12
2009-09-13
2009-09-14
2009-09-15
2009-09-16
2009-09-17
2009-09-18
2009-09-19
2009-09-20
2009-09-21
2009-09-22
2009-09-23
2009-09-24
2009-09-25
2009-09-26
2009-09-27
2009-09-28
2009-09-29
2009-09-30

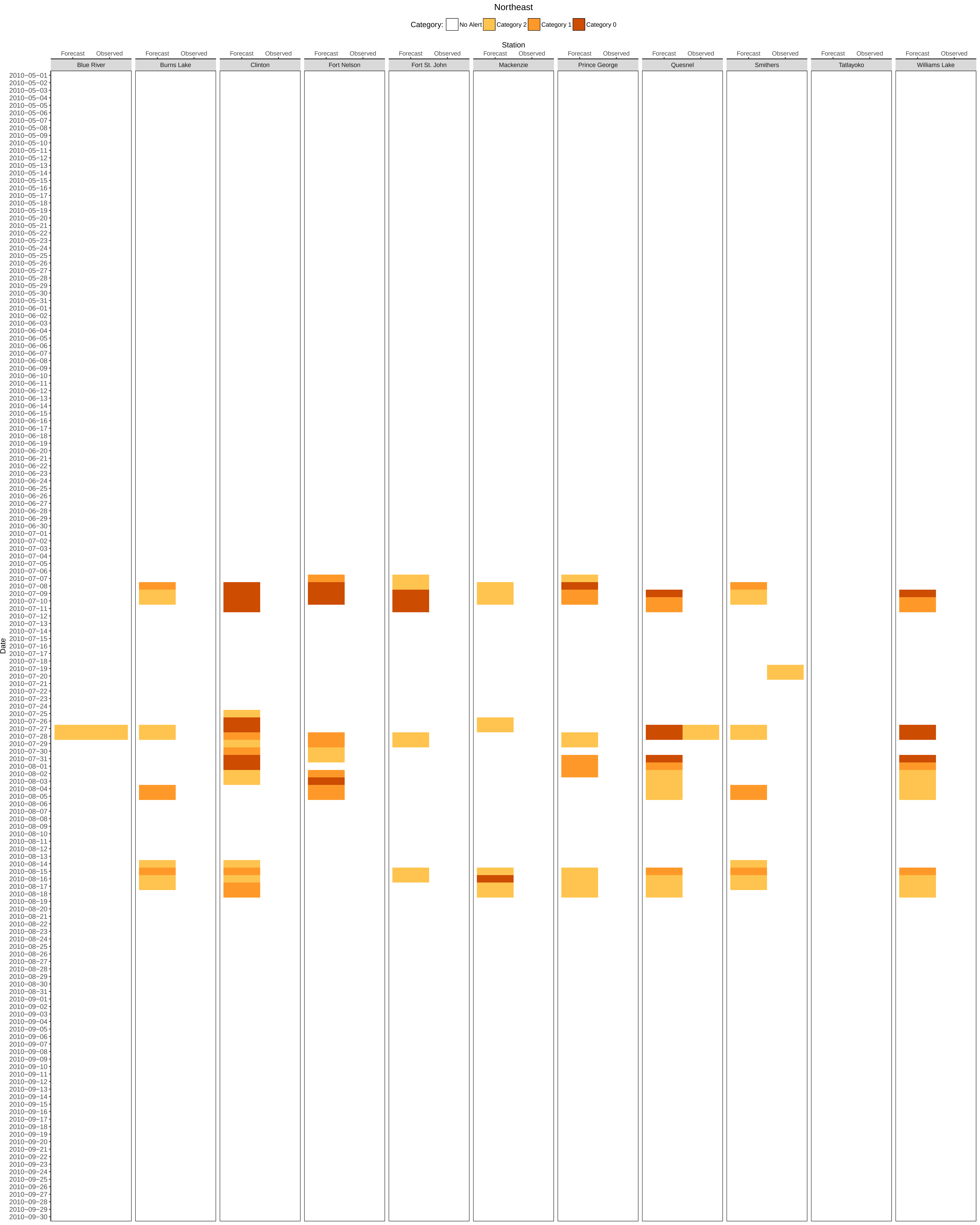
Northeast
Category: No Alert Category 2 Category 1 Category 0
Station
Forecast Observed
Blue River
Burns Lake
Clinton
Fort Nelson
Fort St. John
Mackenzie
Prince George
Quesnel
Smithers
Tatlayoko
Williams Lake
Date
2010−05−01
2010−09−30

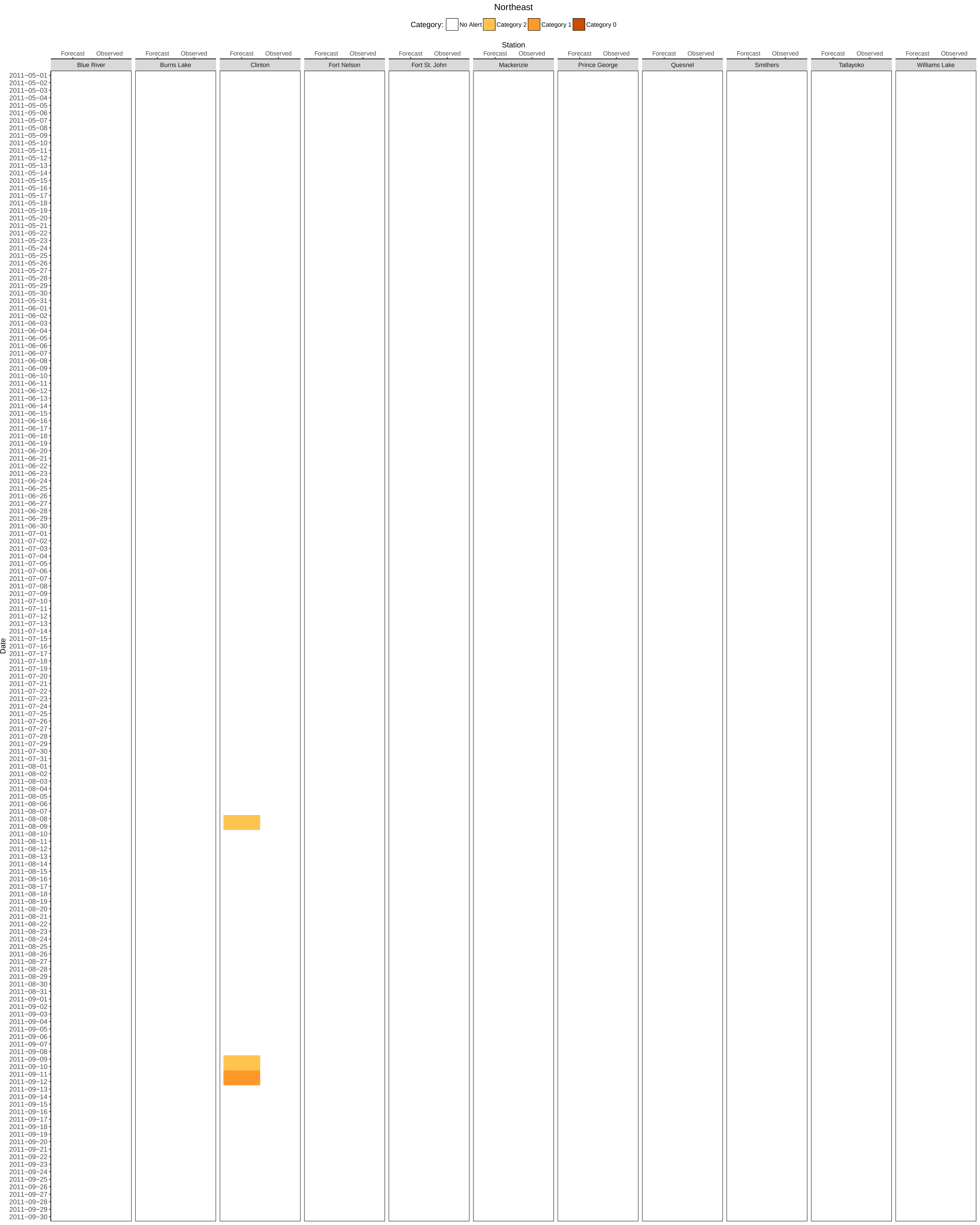
Northeast
Category: No Alert Category 2 Category 1 Category 0
Station
Forecast Observed
Blue River
Burns Lake
Clinton
Fort Nelson
Fort St. John
Mackenzie
Prince George
Quesnel
Smithers
Tatlayoko
Williams Lake
Date
2011−05−01
2011−09−30

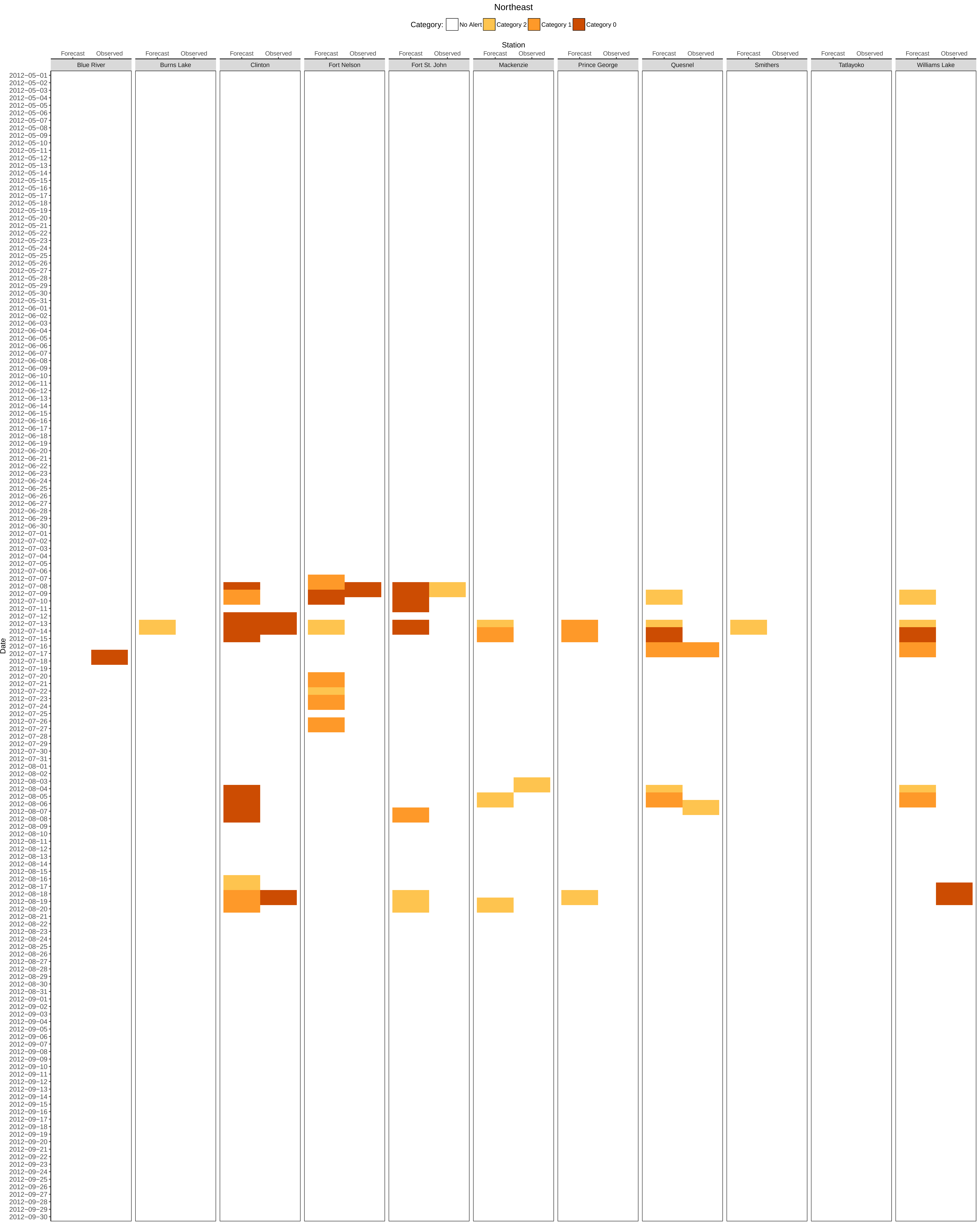

Northeast
Category: No Alert Category 2 Category 1 Category 0
Station
Forecast Observed
Blue River
Burns Lake
Clinton
Fort Nelson
Fort St. John
Mackenzie
Prince George
Quesnel
Smithers
Tatlayoko
Williams Lake
Date
2012−05−01
2012−09−30

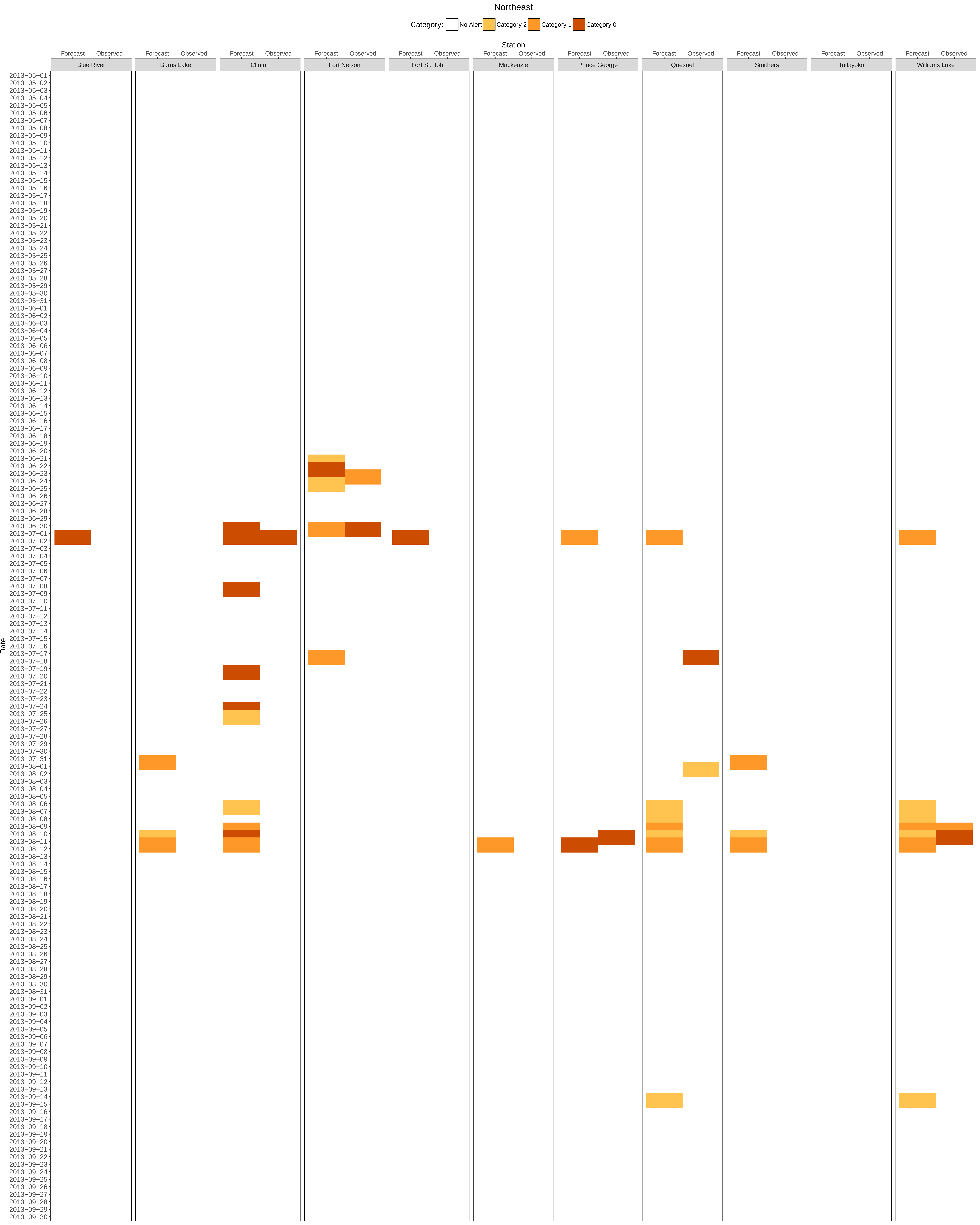

Northeast
Category:
No Alert
Category 2
Category 1
Category 0
Station
Forecast
Observed
Blue River
Burns Lake
Clinton
Fort Nelson
Fort St. John
Mackenzie
Prince George
Quesnel
Smithers
Tatlayoko
Williams Lake
Date
2013-05-01
2013-05-02
2013-05-03
2013-05-04
2013-05-05
2013-05-06
2013-05-07
2013-05-08
2013-05-09
2013-05-10
2013-05-11
2013-05-12
2013-05-13
2013-05-14
2013-05-15
2013-05-16
2013-05-17
2013-05-18
2013-05-19
2013-05-20
2013-05-21
2013-05-22
2013-05-23
2013-05-24
2013-05-25
2013-05-26
2013-05-27
2013-05-28
2013-05-29
2013-05-30
2013-05-31
2013-06-01
2013-06-02
2013-06-03
2013-06-04
2013-06-05
2013-06-06
2013-06-07
2013-06-08
2013-06-09
2013-06-10
2013-06-11
2013-06-12
2013-06-13
2013-06-14
2013-06-15
2013-06-16
2013-06-17
2013-06-18
2013-06-19
2013-06-20
2013-06-21
2013-06-22
2013-06-23
2013-06-24
2013-06-25
2013-06-26
2013-06-27
2013-06-28
2013-06-29
2013-06-30
2013-07-01
2013-07-02
2013-07-03
2013-07-04
2013-07-05
2013-07-06
2013-07-07
2013-07-08
2013-07-09
2013-07-10
2013-07-11
2013-07-12
2013-07-13
2013-07-14
2013-07-15
2013-07-16
2013-07-17
2013-07-18
2013-07-19
2013-07-20
2013-07-21
2013-07-22
2013-07-23
2013-07-24
2013-07-25
2013-07-26
2013-07-27
2013-07-28
2013-07-29
2013-07-30
2013-07-31
2013-08-01
2013-08-02
2013-08-03
2013-08-04
2013-08-05
2013-08-06
2013-08-07
2013-08-08
2013-08-09
2013-08-10
2013-08-11
2013-08-12
2013-08-13
2013-08-14
2013-08-15
2013-08-16
2013-08-17
2013-08-18
2013-08-19
2013-08-20
2013-08-21
2013-08-22
2013-08-23
2013-08-24
2013-08-25
2013-08-26
2013-08-27
2013-08-28
2013-08-29
2013-08-30
2013-08-31
2013-09-01
2013-09-02
2013-09-03
2013-09-04
2013-09-05
2013-09-06
2013-09-07
2013-09-08
2013-09-09
2013-09-10
2013-09-11
2013-09-12
2013-09-13
2013-09-14
2013-09-15
2013-09-16
2013-09-17
2013-09-18
2013-09-19
2013-09-20
2013-09-21
2013-09-22
2013-09-23
2013-09-24
2013-09-25
2013-09-26
2013-09-27
2013-09-28
2013-09-29
2013-09-30

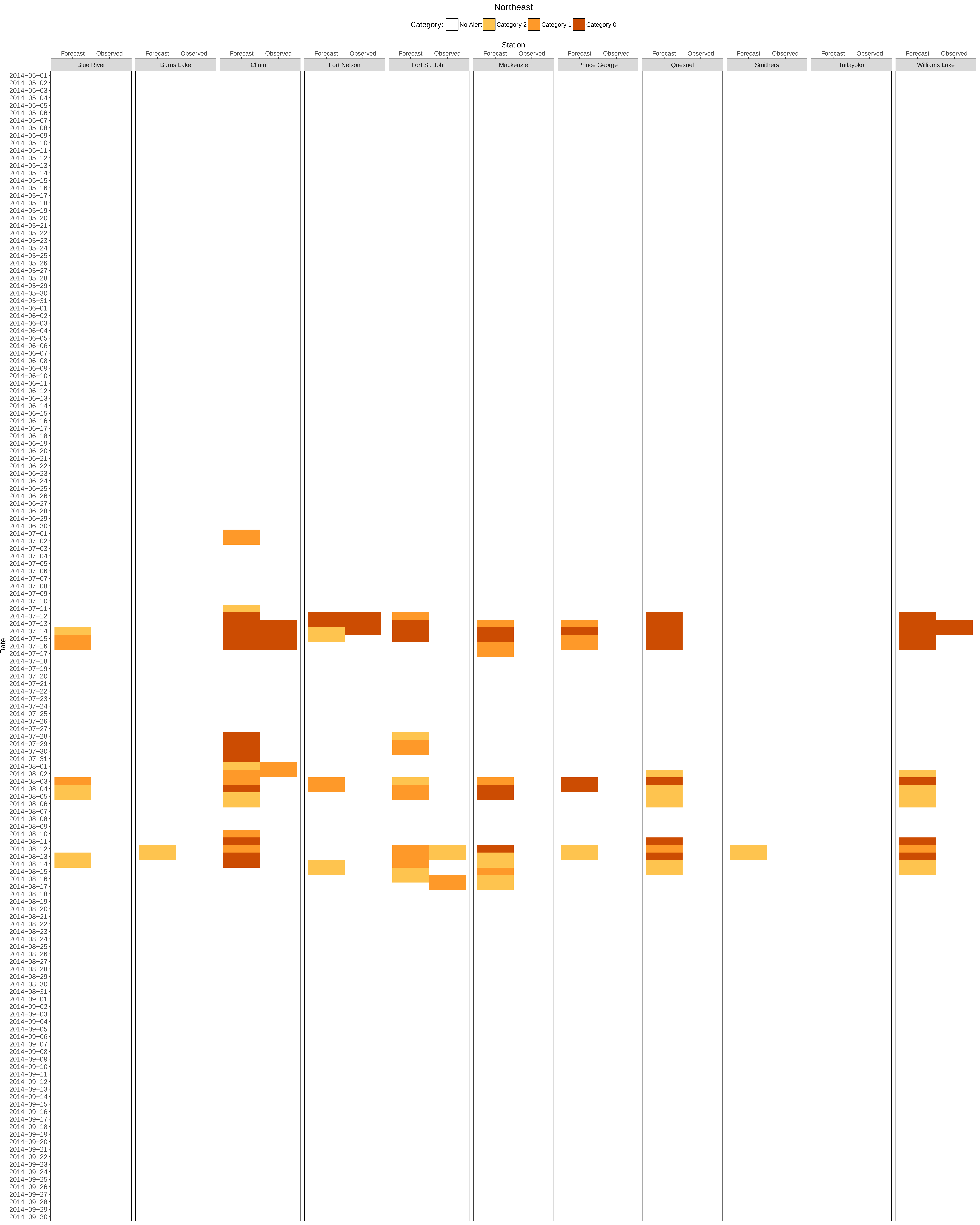
Northeast
Category:
No Alert
Category 2
Category 1
Category 0
Station
Forecast
Observed
Blue River
Burns Lake
Clinton
Fort Nelson
Fort St. John
Mackenzie
Prince George
Quesnel
Smithers
Tatlayoko
Williams Lake
Date
2014-05-01
2014-05-02
2014-05-03
2014-05-04
2014-05-05
2014-05-06
2014-05-07
2014-05-08
2014-05-09
2014-05-10
2014-05-11
2014-05-12
2014-05-13
2014-05-14
2014-05-15
2014-05-16
2014-05-17
2014-05-18
2014-05-19
2014-05-20
2014-05-21
2014-05-22
2014-05-23
2014-05-24
2014-05-25
2014-05-26
2014-05-27
2014-05-28
2014-05-29
2014-05-30
2014-05-31
2014-06-01
2014-06-02
2014-06-03
2014-06-04
2014-06-05
2014-06-06
2014-06-07
2014-06-08
2014-06-09
2014-06-10
2014-06-11
2014-06-12
2014-06-13
2014-06-14
2014-06-15
2014-06-16
2014-06-17
2014-06-18
2014-06-19
2014-06-20
2014-06-21
2014-06-22
2014-06-23
2014-06-24
2014-06-25
2014-06-26
2014-06-27
2014-06-28
2014-06-29
2014-06-30
2014-07-01
2014-07-02
2014-07-03
2014-07-04
2014-07-05
2014-07-06
2014-07-07
2014-07-08
2014-07-09
2014-07-10
2014-07-11
2014-07-12
2014-07-13
2014-07-14
2014-07-15
2014-07-16
2014-07-17
2014-07-18
2014-07-19
2014-07-20
2014-07-21
2014-07-22
2014-07-23
2014-07-24
2014-07-25
2014-07-26
2014-07-27
2014-07-28
2014-07-29
2014-07-30
2014-07-31
2014-08-01
2014-08-02
2014-08-03
2014-08-04
2014-08-05
2014-08-06
2014-08-07
2014-08-08
2014-08-09
2014-08-10
2014-08-11
2014-08-12
2014-08-13
2014-08-14
2014-08-15
2014-08-16
2014-08-17
2014-08-18
2014-08-19
2014-08-20
2014-08-21
2014-08-22
2014-08-23
2014-08-24
2014-08-25
2014-08-26
2014-08-27
2014-08-28
2014-08-29
2014-08-30
2014-08-31
2014-09-01
2014-09-02
2014-09-03
2014-09-04
2014-09-05
2014-09-06
2014-09-07
2014-09-08
2014-09-09
2014-09-10
2014-09-11
2014-09-12
2014-09-13
2014-09-14
2014-09-15
2014-09-16
2014-09-17
2014-09-18
2014-09-19
2014-09-20
2014-09-21
2014-09-22
2014-09-23
2014-09-24
2014-09-25
2014-09-26
2014-09-27
2014-09-28
2014-09-29
2014-09-30

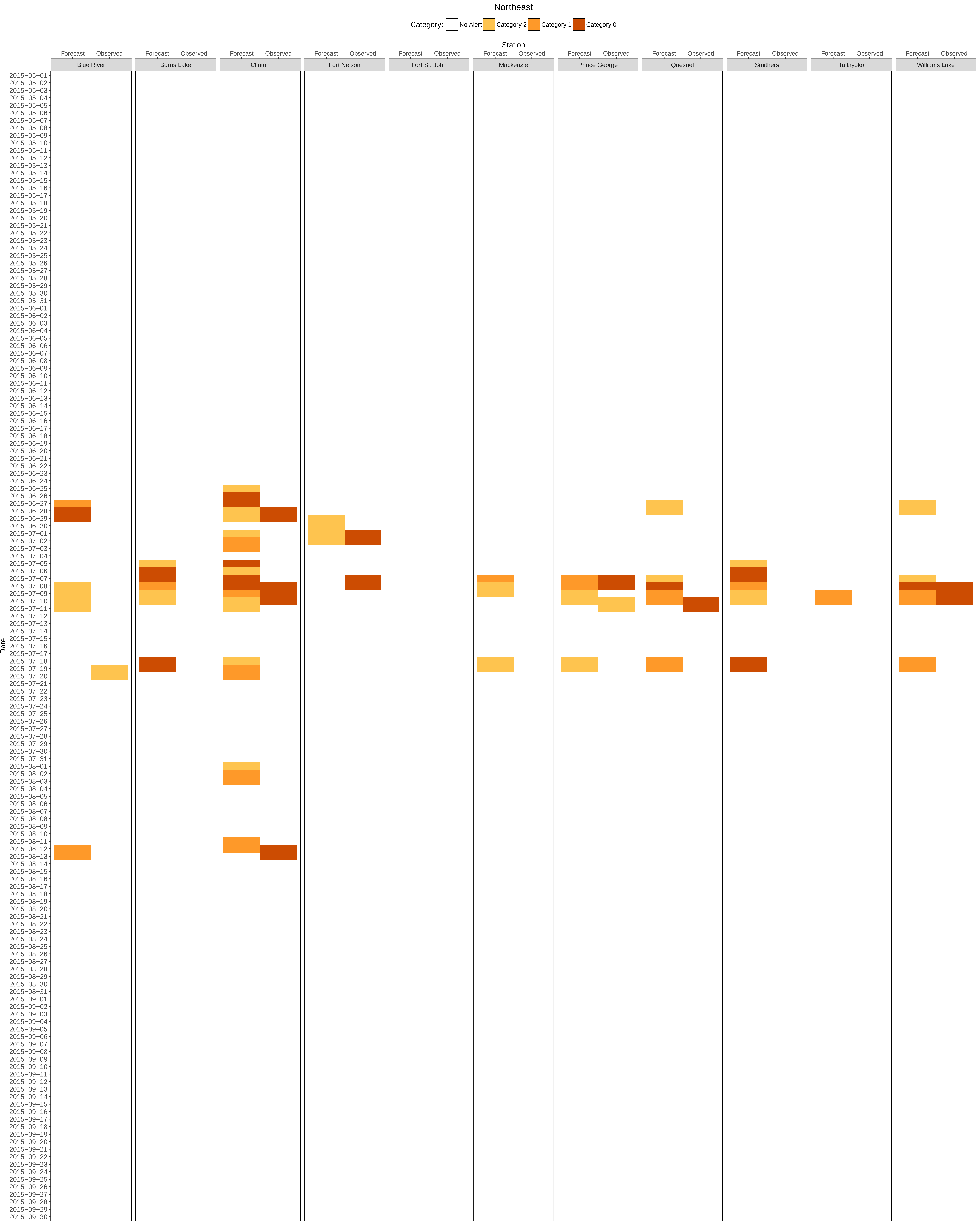
Northeast
Category: No Alert Category 2 Category 1 Category 0
Station
Forecast Observed
Blue River
Burns Lake
Clinton
Fort Nelson
Fort St. John
Mackenzie
Prince George
Quesnel
Smithers
Tatlayoko
Williams Lake
Date
2015−05−01
2015−06−01
2015−07−01
2015−08−01
2015−09−01
2015−09−30

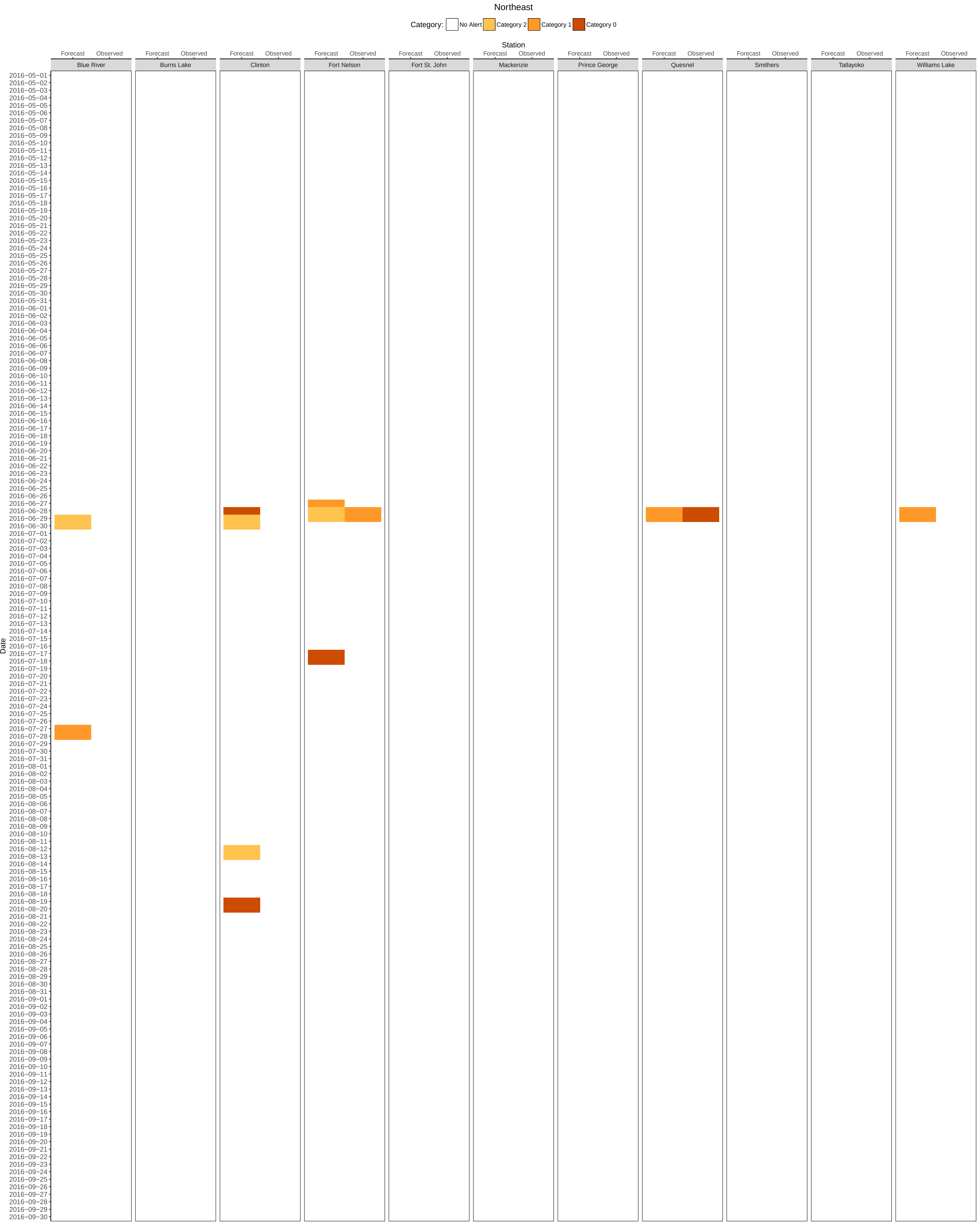
Northeast
Category: No Alert Category 2 Category 1 Category 0
Station
Forecast Observed
Blue River
Burns Lake
Clinton
Fort Nelson
Fort St. John
Mackenzie
Prince George
Quesnel
Smithers
Tatlayoko
Williams Lake
Date
2016−05−01
2016−09−30
