# Supplementary figures and images for: Establishing Heat Alert Thresholds for the Varied Climatic Regions of British Columbia, Canada
Source: Int J Environ Res Public Health. 2018 Sep 19;15(9):2048. doi: 10.3390/ijerph15092048 (PMC6163932; doi:10.3390/ijerph15092048)

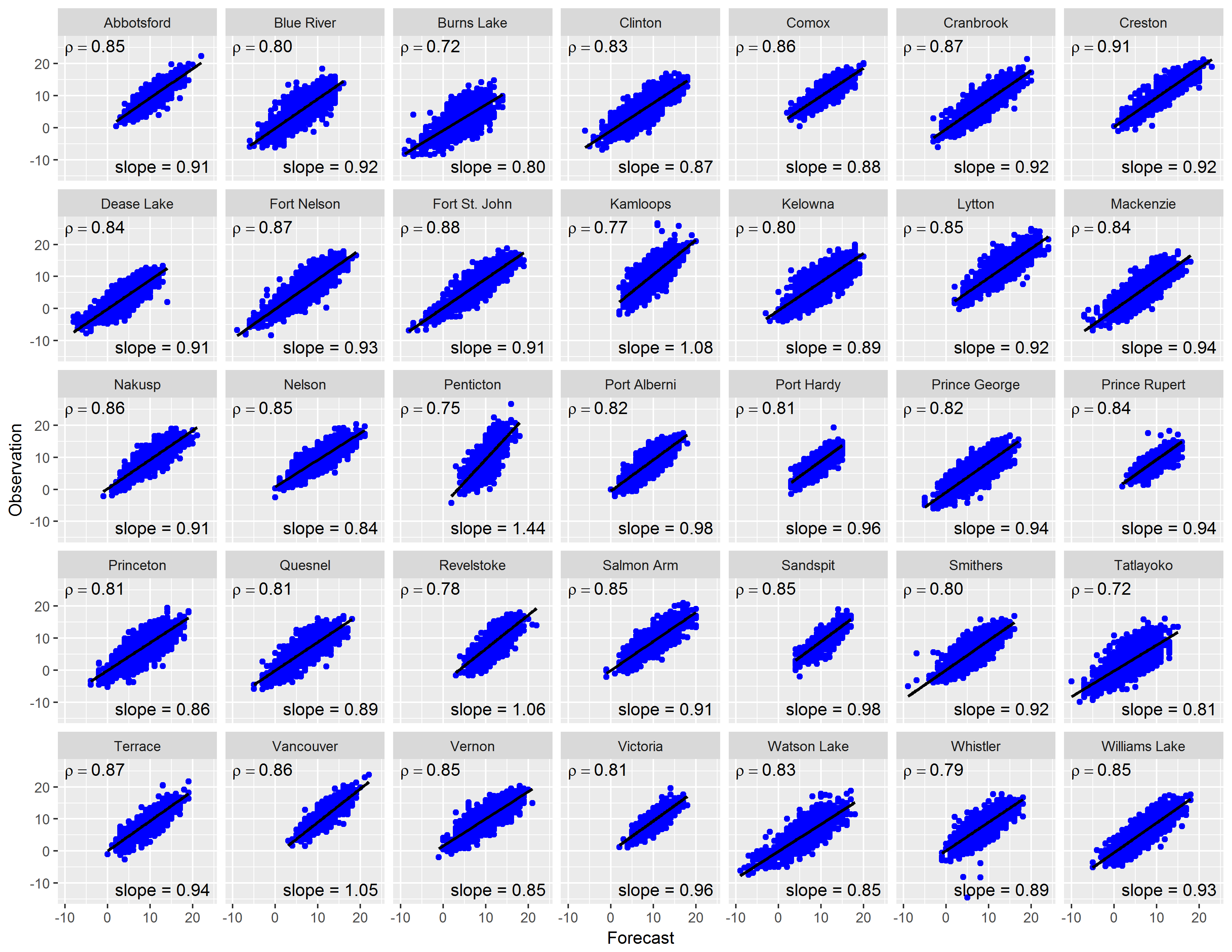

Supplement: Supplementary file 1 [file ijerph-15-02048-s001.zip › ijerph-343571-Supplementary materials-proofreading/ijerph-343571-Supplementary materials-proofreading/S1_Figure_Observed_vs_Forecasted_Min.png]

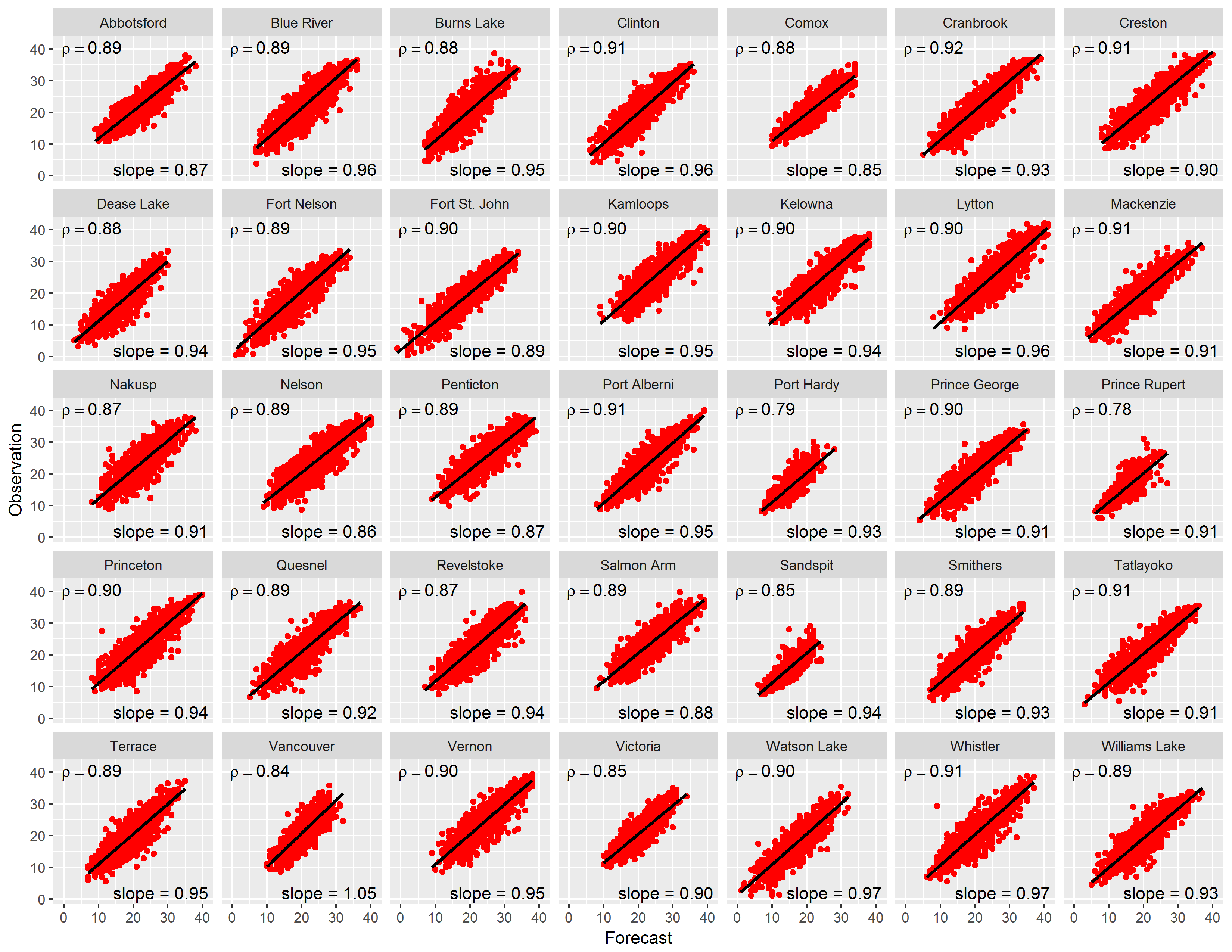

Supplement: Supplementary file 1 [file ijerph-15-02048-s001.zip › ijerph-343571-Supplementary materials-proofreading/ijerph-343571-Supplementary materials-proofreading/S2_Figure_Observed_vs_Forecasted_Max.png]

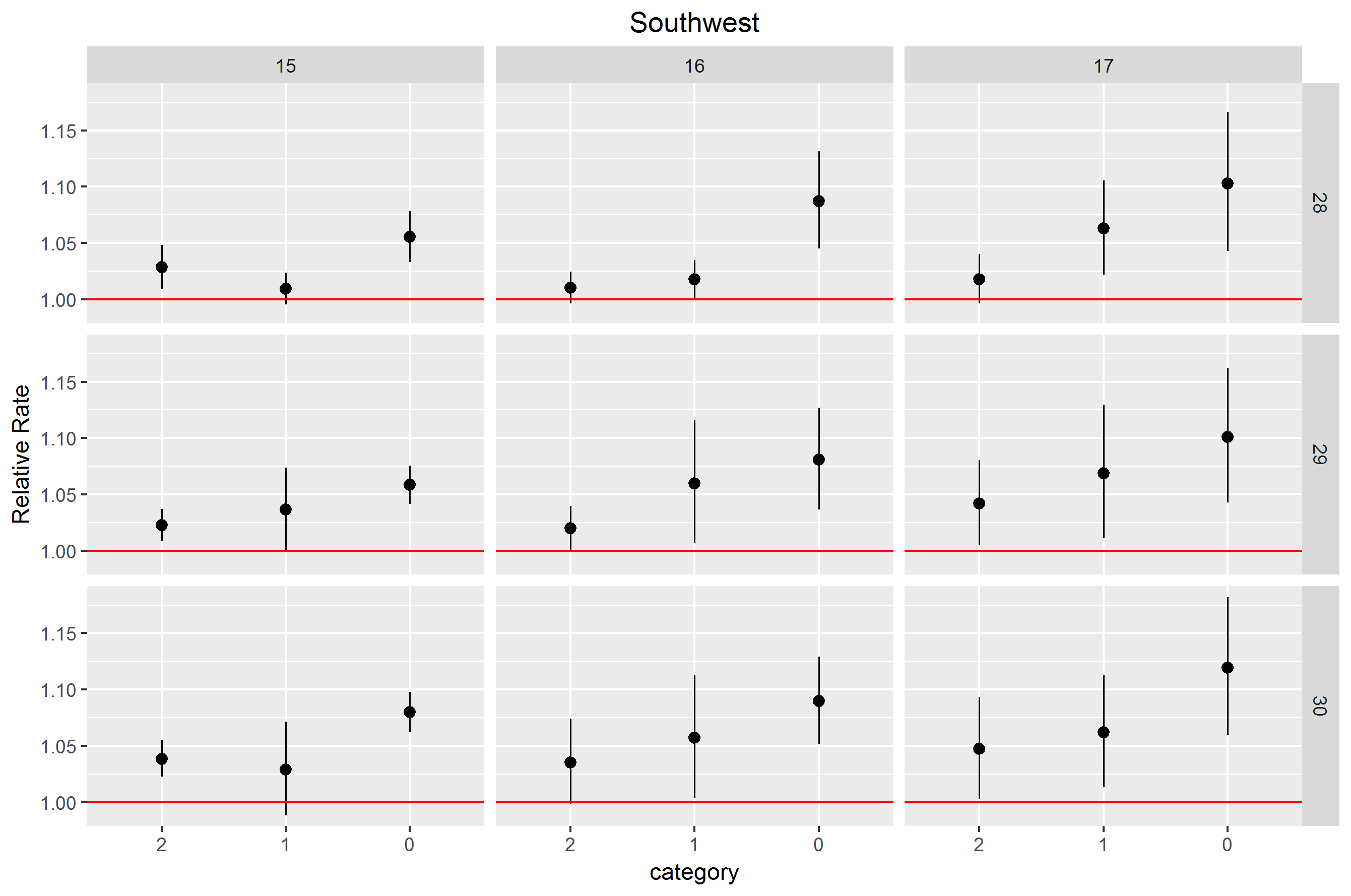

Supplement: Supplementary file 1 [file ijerph-15-02048-s001.zip › ijerph-343571-Supplementary materials-proofreading/ijerph-343571-Supplementary materials-proofreading/S3_Southwest_threshold_combinations.png]

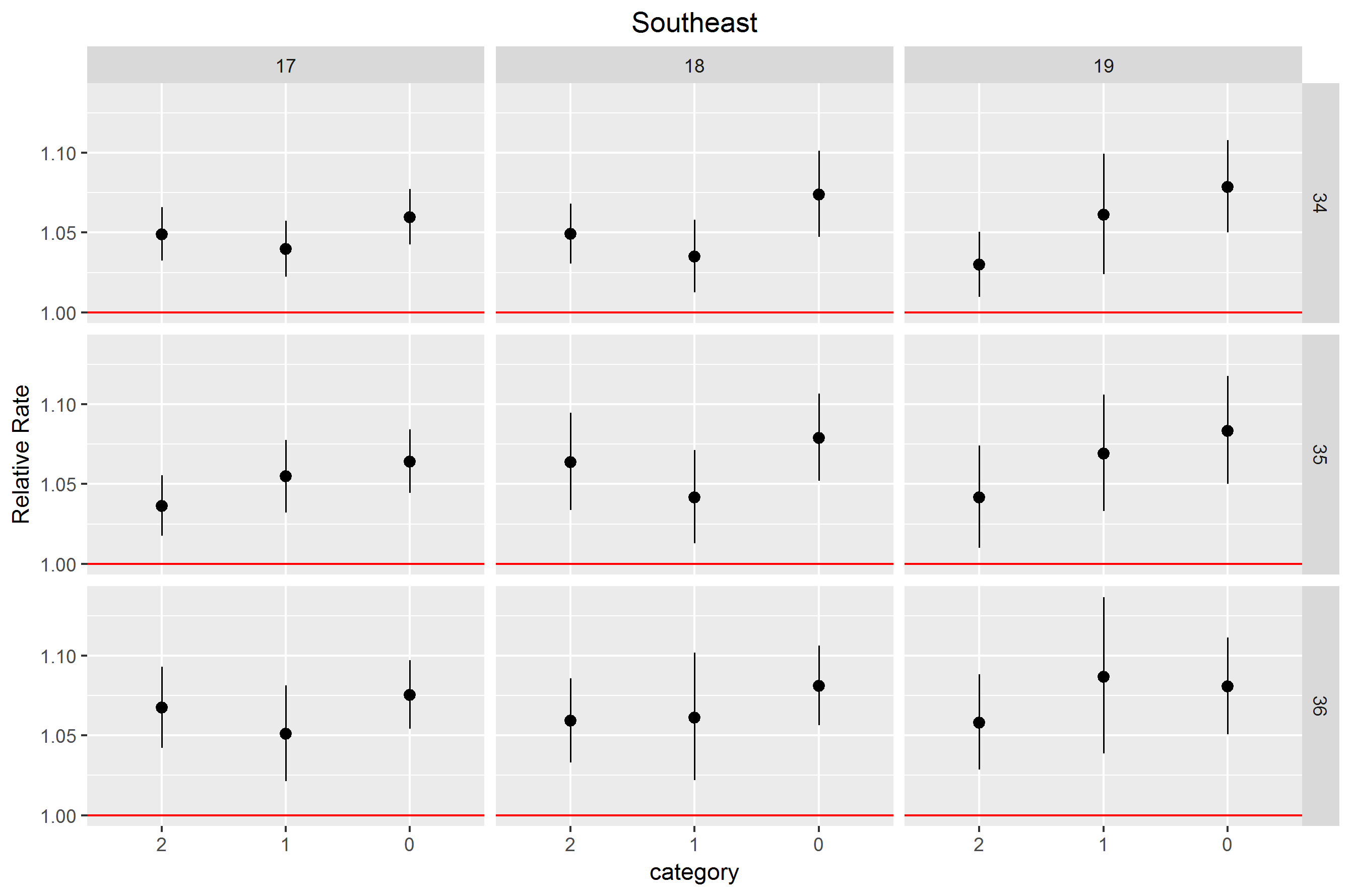

Supplement: Supplementary file 1 [file ijerph-15-02048-s001.zip › ijerph-343571-Supplementary materials-proofreading/ijerph-343571-Supplementary materials-proofreading/S4_Southeast_threshold_combinations.png]

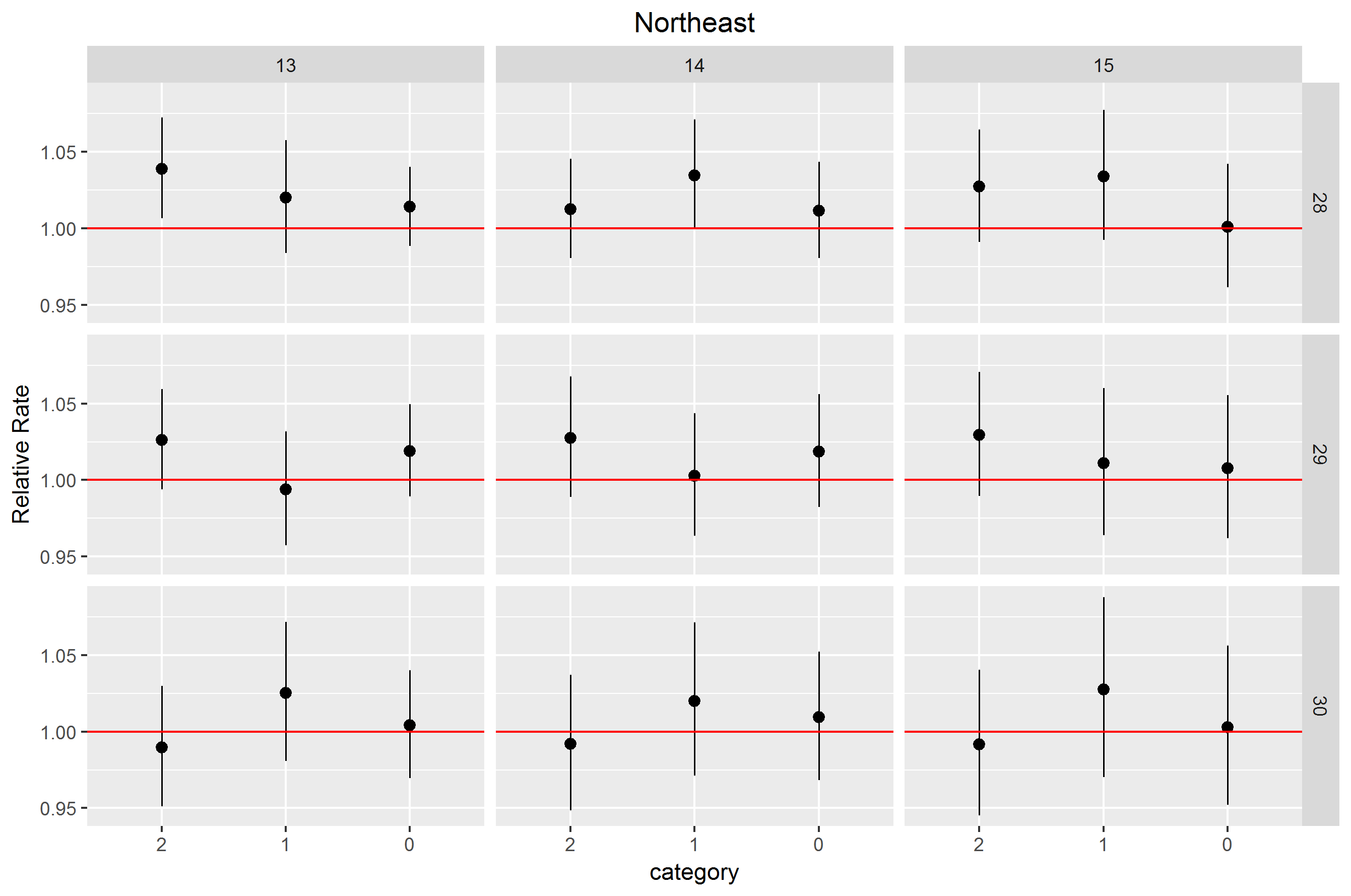

Supplement: Supplementary file 1 [file ijerph-15-02048-s001.zip › ijerph-343571-Supplementary materials-proofreading/ijerph-343571-Supplementary materials-proofreading/S5_Northeast_threshold_combinations.png]

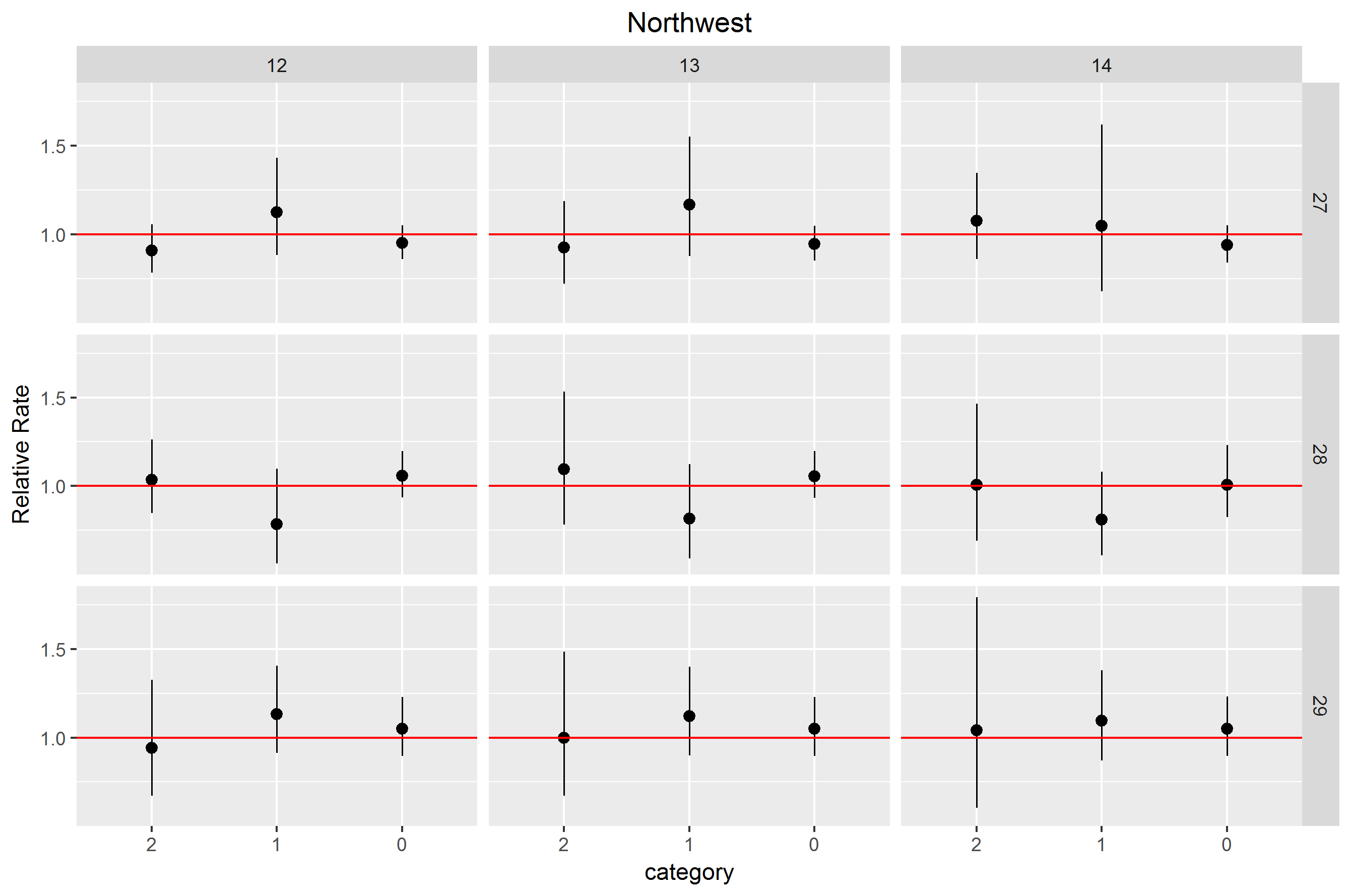

Supplement: Supplementary file 1 [file ijerph-15-02048-s001.zip › ijerph-343571-Supplementary materials-proofreading/ijerph-343571-Supplementary materials-proofreading/S6_Northwest_threshold_combinations.png]

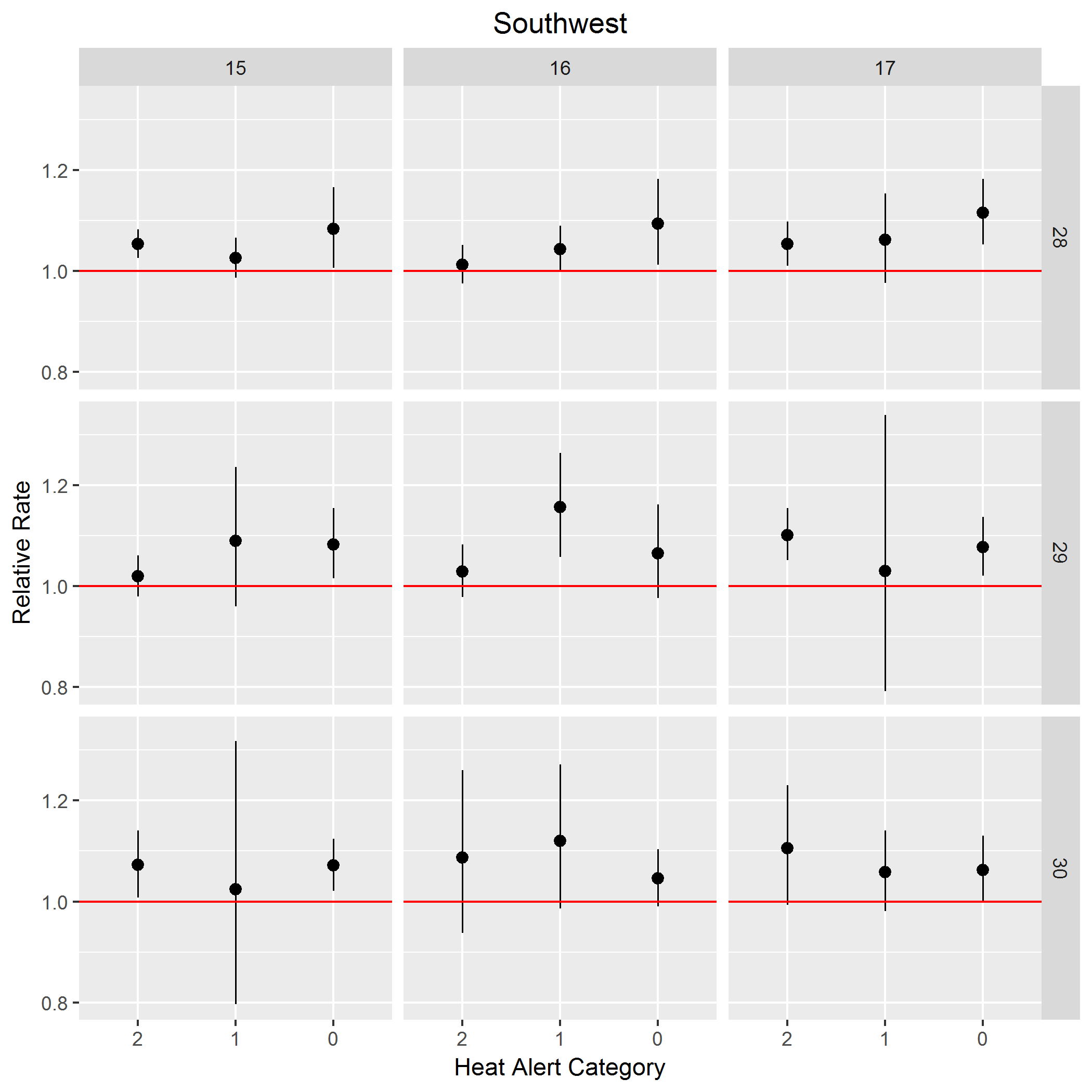

Supplement: Supplementary file 1 [file ijerph-15-02048-s001.zip › ijerph-343571-Supplementary materials-proofreading/ijerph-343571-Supplementary materials-proofreading/sample_code/output/Southwest.png]

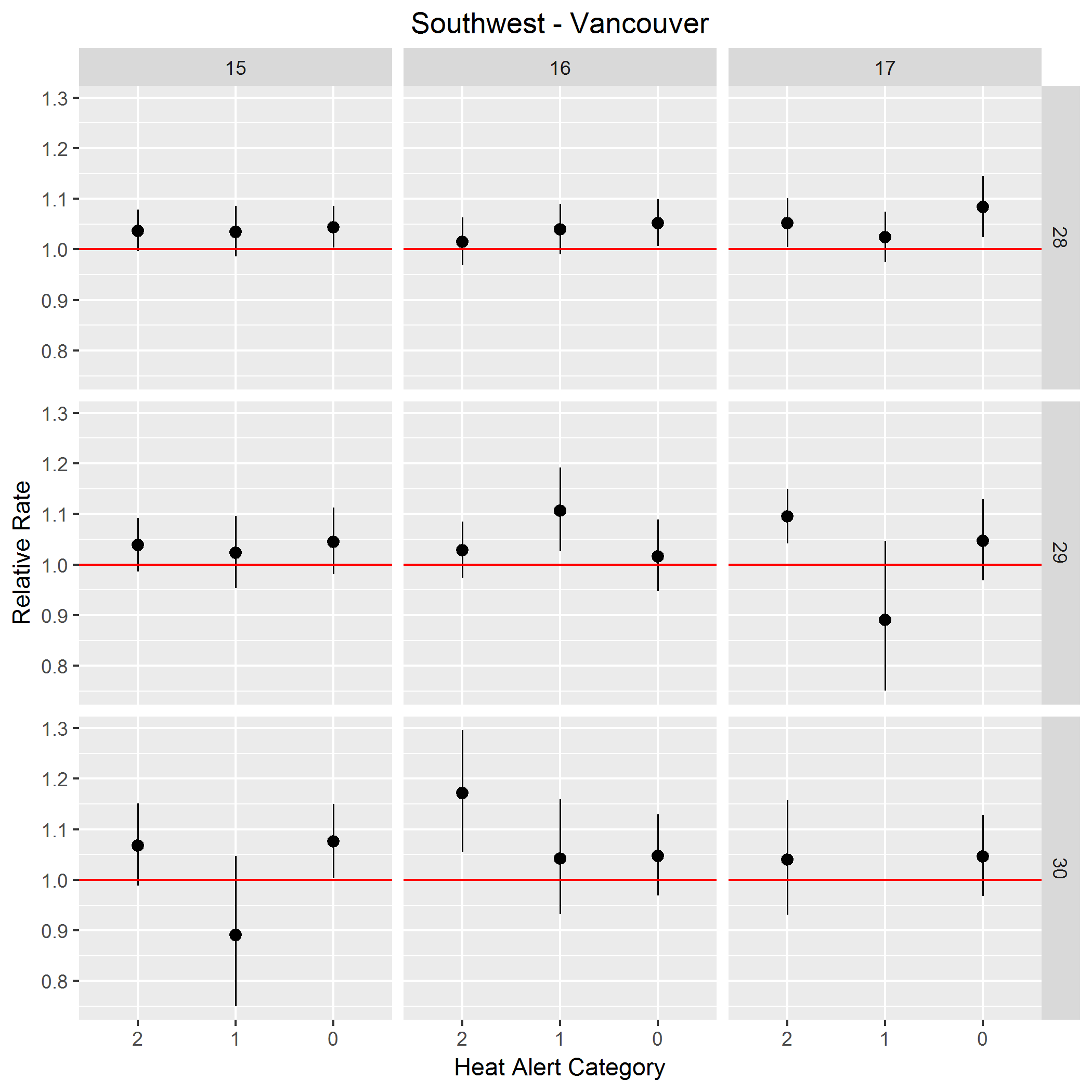

Supplement: Supplementary file 1 [file ijerph-15-02048-s001.zip › ijerph-343571-Supplementary materials-proofreading/ijerph-343571-Supplementary materials-proofreading/sample_code/output/Vancouver.png]

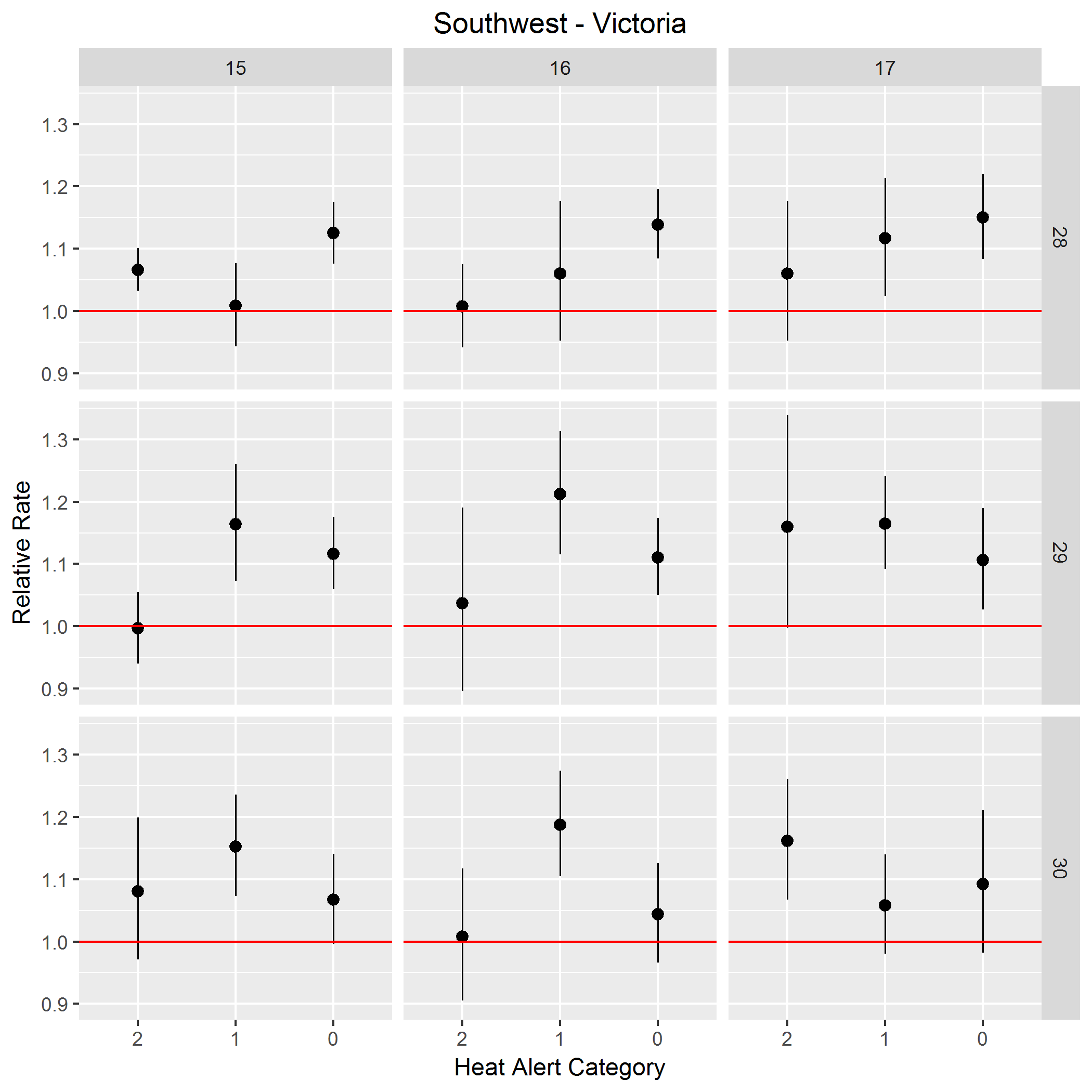

Supplement: Supplementary file 1 [file ijerph-15-02048-s001.zip › ijerph-343571-Supplementary materials-proofreading/ijerph-343571-Supplementary materials-proofreading/sample_code/output/Victoria.png]
